# Metal ions and sugar puckering balance single-molecule kinetic heterogeneity in RNA and DNA tertiary contacts

**DOI:** 10.1038/s41467-019-13683-4

**Published:** 2020-01-08

**Authors:** Fabio D. Steffen, Mokrane Khier, Danny Kowerko, Richard A. Cunha, Richard Börner, Roland K. O. Sigel

**Affiliations:** 1 Department of Chemistry, University of Zurich, Winterthurerstrasse 190, 8057 Zurich, Switzerland; 2 Present Address: Department of Informatics, Technical University Chemnitz, Straße der Nationen 62, 09111 Chemnitz, Germany; 3 Present Address: Laserinstitut Hochschule Mittweida, University of Applied Sciences Mittweida, Technikumplatz 17, 09648 Mittweida, Germany

**Keywords:** Bioinorganic chemistry, Biophysical chemistry, Magnesium, Potassium, RNA

## Abstract

The fidelity of group II intron self-splicing and retrohoming relies on long-range tertiary interactions between the intron and its flanking exons. By single-molecule FRET, we explore the binding kinetics of the most important, structurally conserved contact, the exon and intron binding site 1 (EBS1/IBS1). A comparison of RNA-RNA and RNA-DNA hybrid contacts identifies transient metal ion binding as a major source of kinetic heterogeneity which typically appears in the form of degenerate FRET states. Molecular dynamics simulations suggest a structural link between heterogeneity and the sugar conformation at the exon-intron binding interface. While Mg^2+^ ions lock the exon in place and give rise to long dwell times in the exon bound FRET state, sugar puckering alleviates this structural rigidity and likely promotes exon release. The interplay of sugar puckering and metal ion coordination may be an important mechanism to balance binding affinities of RNA and DNA interactions in general.

## Abstract (web summary)

Mobile group II introns function as ribozymes to splice and reinsert themselves into DNA, thereby colonizing new genomic regions. Here the authors use single-molecule FRET and molecular dynamics simulations to reveal a structural link between metal ion induced kinetic heterogeneity and the sugar puckers at the exon-intron binding interface.

## Introduction

**Fig. 1:**
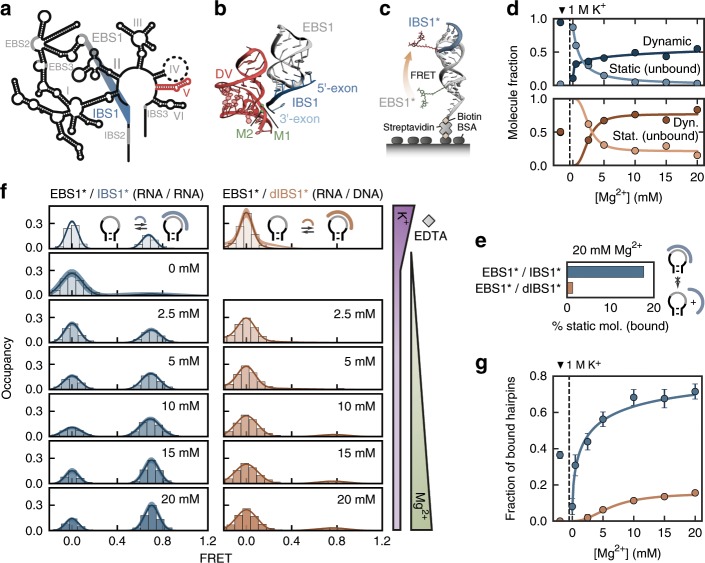
Thermodynamics of Mg^2+^-induced tertiary contact formation from single-molecule FRET. **a** Secondary structure of the group IIB intron Sc.*ai5γ* with the exon–intron binding site 1 (EBS1/IBS1, gray/blue) and the catalytic domain V (red). **b** Crystal structure of the active site of the P.li. *LSUI2* intron (PDB: 4R0D^8^) featuring domain V (DV, red), EBS1/IBS1 (gray/blue), and two catalytic metal ions (M1/M2, green). **c** Surface-immobilized FRET model system EBS1*/IBS1* labeled with Cy3 and Cy5 (BSA, streptavidin, and biotin are not drawn to scale). **d** Relative abundance of dynamic and statically unbound IBS1* (blue) or dIBS1* (orange) molecules as a function of Mg^2+^. **e** Percentage of long-lived, statically bound molecules at 20 mM Mg^2+^ and 100 mM K^+^. **f** FRET histograms of dynamic molecules showing a rising population of the high FRET state with increasing Mg^2+^ concentration. **g** Fraction of EBS1* hairpins bound to (**d**) IBS1* as a function of the Mg^2+^ concentration calculated from the integrals of the FRET states. Error bars correspond to the mean ± 2 s.d. of 100 bootstrap samples. Source data are provided as a Source Data file.

In the course of RNA maturation, self-splicing ribozymes catalyze two consecutive reactions, that is, the excision of the intron and the ligation of its flanking exons^1^. To precisely locate the cleavage site, group I and II introns engage specific RNA recognition elements that base pair with complementary stretches on the exons (Fig. 1a)^2–4^. In group II introns, these sequences are not conserved, instead the RNA identifies the structural transition between single- and double-stranded bases as the 5′-splice site^5^. The long-range tertiary interactions embed the 5′-exon in the active core of the ribozyme (Fig. 1b)^6–8^, where a hydrogen-bond network and several coordinating metal ions convey stability to the tertiary contacts^9,10^. Two Mg^2+^ directly participate in catalysis by activating the scissile bond for cleavage^11^. Ideally, the metal ions fine-tune the interaction such that the 5′-exon is held in place until the second transesterification has occurred and the two exons are ligated. If the interaction between intron and exon is too weak, splicing either does not occur at all (intron retention) or stops after the first step, with the 5′-exon leaving the active site without being ligated. Conversely, if the association of intron and exon is too strong, the ribozyme no longer discriminates between correct and mismatched targets, which may lead to gene disruption and disease if reverse splicing occurs in tumor suppressor genes like p53^12,13^. To minimize such errors, most group II introns use two independent exon recognition sites to keep hold of the 5′-exon: exon binding site 1 (EBS1) contributes the most to thermodynamic stability, while the surface-exposed EBS2 is responsible for target selectivity^13,14^. Mono- and divalent ions play their part in strengthening the intron–exon interaction both through non-specific charge screening as well as site-directed coordination. A number of such specific binding sites have been identified near active site elements^9,10^. Like a padlock, the metal ion packs the strands together, thereby retaining the exon in the active site for a longer time than if no gatekeeping ion was present. As ion coordination is transient though, designated binding pockets are often only partially occupied and exon dissociation is thus kinetically heterogeneous^15^. Particularly, Mg^2+^ is known to induce such kinetic partitioning by interacting with RNA directly (inner-sphere coordination) or via a water molecule (outer-sphere coordination)^16–18^.

Single-molecule techniques are well suited to detect subpopulations of molecules in different conformational states^15,19–21^. This involves ion-induced collapse of RNA secondary structure elements as well as formation of more distant tertiary contacts^17^. Spectroscopic rulers such as Förster resonance energy transfer (FRET) capture these rearrangements based on a set of predefined distance coordinates. Yet, in some cases, be it due to the choice of the dye positions or the local geometry of the coordination environment, metal ion binding yields no detectable change in resonance energy transfer and results in a degenerate FRET state that comprises multiple kinetic states. Their existence is only inferred from the multiexponential decay rates from the one FRET state.

As metal ions direct folding and catalysis, heterogeneity is inherent to many RNAs^16,22^. RNA folds hierarchically into a set of interconnected topological modules. The kinetics of their tertiary assembly is often rate determining in the folding process^17^. Pseudoknots are a recurrent motif in riboswitches, ribozymes, and the ribosome where they interface secondary and tertiary structure. They are often involved in the formation of the catalytic core or the aptamer domain^23–26^. In its simplest form, an H-type pseudoknot consists of a hairpin that base pairs intramolecularly with a stretch of nucleotides outside the stem^27^. In group II introns such a hairpin reaches out to the flanking exon and forms the recognition site for splicing and retrohoming^2,28–30^. Here we use single-molecule FRET in combination with hidden Markov modeling to probe the pairing of EBS1 in the prototypical group II intron Sc.*ai5γ* with its cognate intron binding site 1 (IBS1). To facilitate the interpretation of the binding free energy landscape, we have isolated the tertiary contact and tagged both interaction partner covalently with a fluorescent dye. In this way, we establish FRET as a reaction coordinate, which directly reports on the binding of exon and intron. Formation of this contact is a prerequisite for subsequent catalysis. While structures of the exon–intron complex before the first step of forward and reverse splicing have been solved recently^7,31,32^, dynamic information on the association and dissociation reaction is still largely missing to date. By comparing how RNA and DNA exons bind to the isolated EBS1 hairpin, we can dissect the kinetics of the recognition step, which precedes forward and reverse splicing.

We find that RNA–DNA hybrids not only dissociate much faster than their pure RNA counterparts, but they do so in a more kinetically homogeneous manner. Our molecular dynamics simulations show that the origin lies in the conformational fit between the binding partners. Fast switching of the sugar puckers in the DNA exon compromises tertiary contact stability and likely impairs metal ion binding. This smooths out the rugged energy landscape and homogenizes the kinetics^33^. Hence, sugar puckering is an effective way to counteract ion-induced heterogeneity in exon recognition and is a potentially widespread mechanism to modulate macromolecular interactions.

## Results

### DNA target recognition requires Mg^2+^

We monitor the binding and unbinding of two seven nucleotides long exon strands, IBS1* (RNA) and dIBS1* (DNA), to surface-immobilized EBS1* hairpins by following the anti-correlated Cy3 and Cy5 emission over several minutes (Fig. 1c and Supplementary Fig. 1). The two intensity signals are converted into transfer efficiencies that fluctuate between a zero FRET state, corresponding to the unbound hairpin, and a high FRET state around 0.75 of the formed tertiary contact. We classified the molecules according to their interconversion frequency into (i) static zero (no binding event within observation time), (ii) static high (no unbinding event), (iii) one transition and (iv) dynamic (at least two transitions). The relative abundance of all four classes across different Mg^2+^ concentrations is summarized in Supplementary Fig. 2. Both RNA–RNA and RNA–DNA contacts are remarkably sensitive to Mg^2+^, as indicated by the strong depletion of static zero FRET molecules in favor of dynamic molecules when Mg^2+^ is added (Fig. 1d). Static traces in the high FRET state only appear in the presence of IBS1*, but not dIBS1* (Fig. 1e). These persistent contacts (>400 s) are unique to the RNA–RNA contact and are strictly dependent on Mg^2+^ (Supplementary Fig. 2). This suggests that Mg^2+^ not only condenses around the RNA but binds site specifically to the tertiary contact. Further evidence for such a binding site comes from nuclear magnetic resonance (NMR) chemical shift mappings with Mg^2+^ and [Co(NH_3_)_6_]^3+^, as well as Mn^2+^-induced line broadening^34,35^. The affected nucleotides coincide with a patch of negative surface potential near the 5′-splice site. Upon docking of IBS1* or dIBS1*, a cavity is formed, which readily accommodates a Mg^2+^ ion^34,35^.

Divalent ions are therefore expected to promote the interaction of exon and intron by a combination of non-specific charge compensation and site-specific binding^18,36^. The fraction of formed contacts are deduced from FRET efficiency histograms of all dynamic molecules (Fig. 1f). The relative occupancy of the high FRET state increases as a function of the Mg^2+^ concentration (Fig. 1g). The normalized binding isotherm saturates at around 70% of bound EBS1*/IBS1* molecules. The fraction of bound RNA–DNA on the contrary does not exceed 20% even at 20 mM Mg^2+^. Complete saturation of the hairpin would require exon concentrations in the high micromolar to millimolar range, which is inaccessible to single-molecule total internal reflection fluorescence (TIRF) imaging because of the high background signal (Supplementary Fig. 3). The dissociation constant calculated from the equilibrium population of the zero and high FRET states shows that EBS1*/dIBS1* is about an order of magnitude less stable than EBS1*/IBS1* (Supplementary Fig. 4a). This observation is consistent with previous reports of RNA–DNA hybrid duplexes being more labile than their canonical RNA–RNA counterparts^37–39^. Notably, no EBS1*/dIBS1* formation at all is observed in the absence of Mg^2+^, highlighting the importance of divalent ions in stabilizing tertiary structure motifs. The free energy contribution of Mg^2+^ binding to tertiary contact formation is given by the difference in Mg^2+^ binding to the hairpin alone and to the formed contact, involving both diffuse and site-specific components (Supplementary Information and Supplementary Fig. 4b). Diffuse binding is expected to be similar for RNA and DNA exons, thus the shift of the midpoint of the binding curve (RNA–RNA: [Mg^2+^]_mid_ = 3.7 ± 0.9 mM versus RNA–DNA: [Mg^2+^]_mid_ = 7.9 ± 1.9 mM) suggests a tighter inner- and/or outer-sphere coordination of Mg^2+^ at the RNA–RNA interface in line with previous binding affinities calculated from NMR chemical shifts^15,40^.

### Mg^2+^ induces heterogeneity by slowing down exon dissociation

**Fig. 2:**
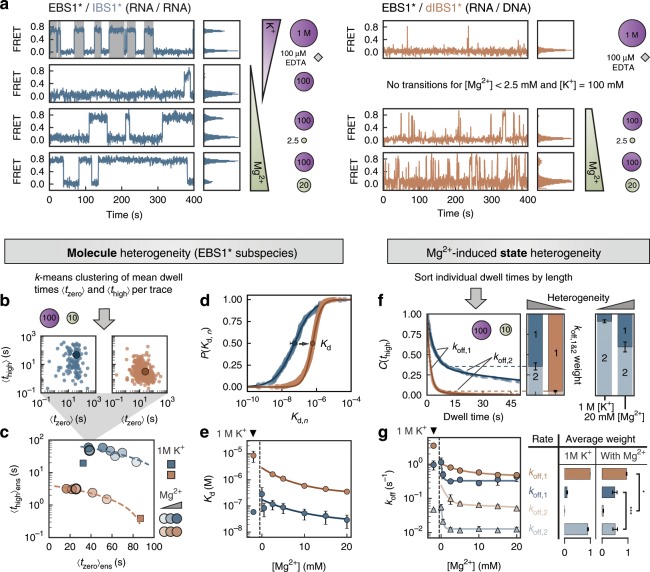
Kinetic heterogeneity is tuned by Mg^2+^ and the nucleic acid strand type. **a** Representative single-molecule traces of EBS1*/IBS1* (blue) and EBS1*/dIBS1* (orange) displaying different residence times in the zero and high FRET state in response to gradual changes in the composition of the ionic environment. The traces are selected to reflect the overall histogram shown in Fig. 1f. The discretized state sequence is depicted by alternating backgrounds (white: zero FRET; gray: high FRET) for the top left trace and omitted elsewhere for clarity. Only dynamic molecules were used for the analysis. **b** The molecule-to-molecule variability is visualized by correlating the mean dwell times in the bound, 〈*t*_high_〉, and unbound state, 〈*t*_zero_〉, for every time trace. There are no distinct kinetic subspecies, but rather a single stretched cluster. Data is shown for 100 mM K^+^ and 10 mM Mg^2+^. **c** The cluster center is calculated by *k*-means and depicted as a circle in different shades of blue or orange for every Mg^2+^ concentration (10 mM Mg^2+^ is highlighted with a thick, black edge). Contact formation under high ionic strength (1 M K^+^) in the absence of Mg^2+^ (100 μM EDTA) is indicated by a blue or orange square. **d** Cumulative *K*_d,*n*_ histograms (10 mM Mg^2+^) are fitted to a logistic function (Supplementary Eq. 13). **e** Stability constants of EBS1*/IBS1* and EBS1/dIBS1 as a function of Mg^2+^ are quantified by the transition point, *K*_d_, from fits in **c**. Error bars are drawn as mean ± 2 s.d. of 100 bootstrap samples. **f** Heterogeneity within the bound state is evaluated by computing cumulative dwell time histograms of the bound state. The presence of multiple decays is a characteristic of kinetic heterogeneity. The weighting factors (mean ± s.d.) of the dissociation rate coefficients *k*_off,1_ and *k*_off,2_ are indicated alongside the decay. The ion dependence of the prefactor is displayed on the right for EBS1*/IBS1*. **g** Rate coefficients and average weighting factors (mean ± s.d.) over the whole Mg^2+^ titration. The star (*) indicates the degree of heterogeneity. The latter is largest if the rate coefficients are far apart and equally populated (weighting factor ≈0.5). Source data are provided as a Source Data file.

Among the four molecular classes defined above, dynamic molecules are the most informative ones as they all represent functional RNA tertiary contacts that associate and dissociate within the observation window. To explore the kinetics of the interaction between the exon and intron binding sites, we discretized the FRET trajectories of the dynamic molecules into bound and unbound segments as shown in Fig. 2a. A visual inspection of the traces reveals both longer dwell times and more frequent transitions to the high FRET state, the more Mg^2+^ is present. By computing a mean dwell time 〈*t*_zero_〉 and 〈*t*_high_〉 for each trace, we can assess how uniformly the immobilized EBS1* hairpins react to IBS1* and dIBS1* binding (Fig. 2b). If there are kinetic subpopulations of EBS1* molecules, some of which bind IBS1* stronger than others, we would expect them to separate into individual clusters in the dwell time scatter plot as suggested by simulations (Supplementary Fig. 5a). On the other hand, if short and long binding events occur within the same trace, they will average out into a single stretched cluster. The width of the distribution is further influenced by the number of dwell times and thus by the limited observation time (Supplementary Fig. 5b, c). There is indeed no clear partitioning of the molecules into separate clusters (low molecule/subspecies heterogeneity, Fig. 2b). We observe, however, a positive correlation between the mean dwell time and the variability within a trace, measured by the difference between the shortest and the longest dwell time (Δ*t*_zero_ or Δ*t*_high_, Supplementary Fig. 6). It follows that the most heterogeneous molecules (large Δ*t*) also feature long mean dwell times.

To test whether these long dwell times are reinforced by divalent metal ions, we monitor the center of the mean dwell time ensemble across different Mg^2+^ concentrations (Fig. 2c and Supplementary Table 1). In the absence of Mg^2+^, diffuse K^+^ ions stimulate strand association mainly through charge compensation, but keep the excursions to the bound state relatively short. On average, IBS1* falls off the hairpin after about 20 s, and dIBS1* unbinds almost immediately after docking (0.4 s). In contrast, addition of 20 mM Mg^2+^ ion triples the average residence time of the IBS1* exon and prolongates the dwell time of dIBS1* by a factor of 10. This goes along with an overall broadening of the distribution of mean dwell times (Supplementary Fig. 7). Hence, we propose that kinetic heterogeneity is induced predominantly by specific binding of Mg^2+^ to preformed tertiary contacts, which slows down the exon dissociation rate. Because Mg^2+^ coordination occurs on a similar timescale as exon unbinding, as shown by previous Mg^2+^ pulse experiments^15^, but is usually faster than the observation time, short- and long-lived dwell times coexist within the same trace.

### Off-rates determine tertiary contact stability

A kinetic comparison of the relatively stronger RNA–RNA and the weaker RNA–DNA interaction is useful in identifying the stability determining rate of tertiary contact formation. It can be viewed as a global ϕ-analysis, where instead of specific mutations, RNA is substituted with the same sequence of DNA to perturb the energetic levels of the bound native state, the transition state, or both^41,42^. We found that the RNA–RNA and RNA–DNA differ mainly in the average time IBS1* or dIBS1* spends bound to the hairpin, that is, 〈*t*_high_〉. In contrast, the average unbound dwell 〈*t*_zero_〉 time is largely independent of the strand type. The ratio of unbound and bound mean dwell times translates into a dissociation constant, *K*_d,*n*_, for each trace. The distribution of all *K*_d,*n*_ is represented in a cumulative histogram and fitted to a logistic function (Supplementary Eq. 13, Fig. 2d, and Supplementary Fig. 8). The *K*_d_ at the inflection point is the expected value of the distribution and a measure for the stability of the tertiary contact. Over the Mg^2+^ titration (0–20 mM) the *K*_d_ of EBS1*/IBS1* decreases by a factor of 10, resulting in an affinity of 29 ± 8 nM at 20 mM Mg^2+^ (Fig. 2e). The *K*_d_ of EBS1*/dIBS1* is about one order of magnitude higher under the same salt conditions (352 ± 8 nM at 20 mM Mg^2+^), reflecting the lower stability of the RNA–DNA hybrid compared to the RNA–RNA, consistent with previous surface plasmon resonance (SPR) experiments (Supplementary Table 2)^35^.

The similar on-rates of the two contacts suggest a reactant-like transition state where the exon and intron are in proximity, but base pairs are not yet fully formed. The energetic barrier originates mostly from the entropic cost of freezing backbone motions as well as uptake and localization of metal ions in the transition state^20,43^. Because the energetic level of the transition state is invariant to the type of exon, the stability of the tertiary contact is dictated by the off-rates. These observations are in line with the emerging paradigm of early transition states being a hallmark of RNA folding^20,24,39,41^. On a rugged energy landscape, it is the rate at which wrongly formed base pairs can be broken again, which limits the overall speed of folding^17^. Mg^2+^ ions provide both shortcuts along the folding route but also trap molecules in misfolded conformations. It is this dual role that leads to the stretched folding times characteristic for many ribozymes^22,44^.

### RNA–DNA is less kinetically heterogeneous

So far, we have looked at the average dwell time of (d)IBS1* in its bound and unbound state. This analysis gives us a robust estimate of the tertiary contact stability from the ensemble of single molecules. On the downside, variations in dwell time length within one trace are averaged out. Mg^2+^ titrations suggest that these variations are caused by ion coordination. Mg^2+^ binding is subtle such that it does not alter the inter-fluorophore distance and consequently ion bound and unbound populations display the very same FRET efficiency. In other words, a single FRET state degenerates into two interconverting kinetic states, each with its own off-rate. We refer to this scenario as state heterogeneity.

To determine the different rates, we computed complementary cumulative dwell time distributions of the bound and unbound state (Fig. 2f, Supplementary Fig. 9, and Supplementary Table 3). Binding kinetics are well described by a single exponential and a stretching factor *β* to account for small deviations at longer times. Unbinding, on the other hand, is not homogeneous, but partitions into two exponential terms in the presence of low millimolar amounts of Mg^2+^. The relative weight of these two components differs considerably depending on the nature of the complementary strand. At 20 mM Mg^2+^, the fast off-rate, *k*_off,1_, accounts for 40 ± 6% of the RNA–RNA contact decay, whereas its amplitude rises to 96 ± 1% in the RNA–DNA contact, meaning the decay is practically homogeneous for the hybrid interaction, even though a small percentage of longer-lived contacts persists (Supplementary Fig. 9).

In either case, off-rates are most sensitive to low millimolar Mg^2+^ concentrations (0–2.5 mM), which coincides with the *K*_d_ of Mg^2+^ to the tertiary contact (Mg^2+^ binding in the loop: 1.78 ± 0.01 mM, binding at the stem-loop transition: 0.87 ± 0.01, Fig. 2g and Supplementary Fig. 10)^15^. A comparison of the IBS1* and dIBS1* dissociation rates reveals that the higher stability of EBS1*/IBS1* over EBS1*/dIBS1* is conveyed by the slower and more prominent *k*_off,2_ of the RNA–RNA contact. The fast *k*_off,1_ is likely to originate from an RNA conformation where no Mg^2+^ is bound, while the slower *k*_off,2_ links to a contact, which is stabilized by one or multiple site-bound Mg^2+^ ions. Since Mg^2+^ binding is transient, these states are exchanging on the timescale of imaging.

### Degenerate FRET state is kinetically resolvable

**Fig. 3:**
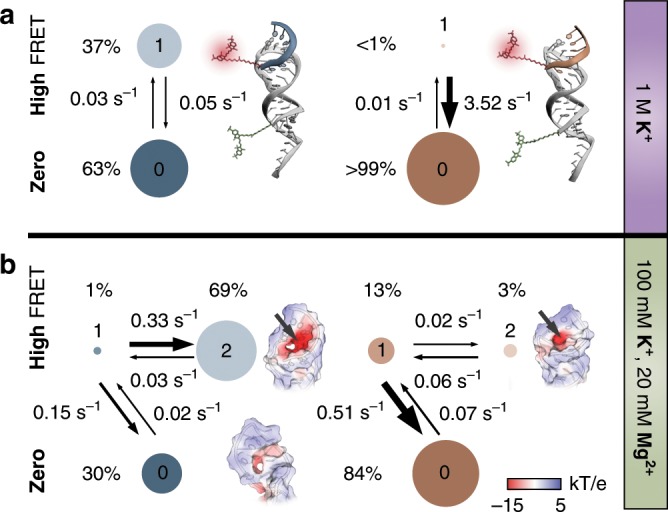
Kinetic model of metal ion-induced IBS1* and dIBS1* recognition. **a** Two-state binding of IBS1* (blue) and dIBS1* (orange) to the EBS1* hairpin under high monovalent ionic strength (0: exon unbound; 1: exon bound). The area of the circle is proportional to the state population (specified in percentage) and the arrow width reflects the relative rate of the transition. Binding rates, *k*′_on,01_, are indicated as pseudo-first-order rates coefficients calculated from the trained HMM. **b** Three-state model with two degenerate high FRET states reflecting the coordination of Mg^2+^ to the formed tertiary contact (0: exon unbound, Mg^2+^ unbound; 1: exon bound, Mg^2+^ unbound; 2: exon bound, Mg^2+^ bound). Insets show surface representations of the RNA–RNA and RNA–DNA interaction, color coded by the electrostatic potential (red: −15 kT e^−1^; blue 5 kT e^−1^). Arrows indicate coordination sites for Mg^2+^. Source data are provided as a Source Data file.

**Fig. 4:**
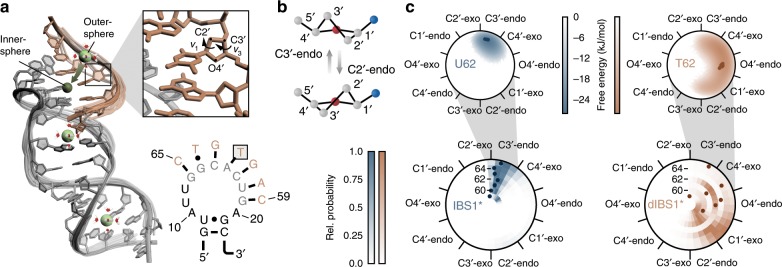
Sugar pucker distributions from all-atom MD simulations. **a** NMR structure of EBS1*/dIBS1* (PDB: 2m1v^34^) with overlaid backbones of the 18 lowest energy structures. Outer-sphere binding sites based on NOE restraints from dIBS1* protons (i.e., H3 and CH_3_ of T62/64 and H5/6 of C65) to [Co(NH_3_)_6_]^3+^ (large green spheres; H_2_O molecules are added for clarity) and a potential inner-sphere coordination mode where Mg^2+^ binds further inside the tunnel (smaller dark green sphere)^35^. **b** Switch between energetically favored sugar pucker conformations C3′-endo and C2′-endo. **c** Circular histogram of pseudorotation angles along the seven nucleotides of IBS1* or dIBS1* (C59–C65) from MD simulations with 100 mM K^+^ and 20 mM Mg^2+^. Insets show distribution of pucker phases and amplitudes of U62 and T62. Puckers of the lowest energy NMR structures and their medians are indicated as dots for each residue. Source data are provided as a Source Data file.

To identify all interconversion rates between bound and unbound species, we used a maximum-likelihood approach that trains a global hidden Markov model (HMM) on all dynamic FRET traces^45^. We tested various kinetic networks and found a sequential three-state model with one zero and a twofold degenerate, high FRET state describing the data best whenever Mg^2+^ is present (Fig. 3b, Table 1, and Supplementary Fig. 11 and Supplementary Table 4, see also Supplementary Discussion). The most appropriate model has been selected based on the Bayesian information criterion (BIC, Supplementary Fig. 12) and prior biochemical knowledge about the system as outlined below. Furthermore, it is critically evaluated by re-simulating the obtained rate system and by reproducing experimental dwell time distributions (Supplementary Figs. 13 and 14 and Supplementary Table 5). Biochemically, the kinetic model couples IBS1* or dIBS1* binding to Mg^2+^ coordination and can be interpreted in the following way: docking of the complement to the hairpin involves a detectable change in FRET efficiency, followed by a state transition, which is insensitive to FRET, thus degenerate, but kinetically resolvable. We attribute this second transition to one or multiple Mg^2+^ ions that specifically coordinate to the exon bound hairpin. Two key observations support this hypothesis: (i) in the absence of Mg^2+^, the mechanism simplifies to a two-state system with one on- and one off-rate (Fig. 3a and Table 1); (ii) as seen from the NMR structure, a patch of negative electrostatic surface potential is exposed once IBS1* or dIBS1* binds to EBS1*, thereby forming a cavity where Mg^2+^ can coordinate. This pocket is particularly pronounced in the RNA–RNA interaction and absent when no exon is bound. There are two alternative metal ion-binding modes at the exon–intron interface between EBS1*/dIBS1* (Fig. 4a): an outer-sphere bound ion has been proposed based on nuclear Overhauser enhancement (NOE) restraints from the nucleic acids to the NMR-active [Co(NH_3_)_6_]^3+^ (green sphere)^35^. Alternatively, a Mg^2+^ may exchange one or more of its coordinated water molecules with RNA atoms and bind deeper within the pocket (dark green sphere). It seems likely that inner-sphere binding of Mg^2+^ in that tunnel locks the tertiary contact in a rigid and stable conformation, which prevents fast dissociation of IBS1*. In the HMM model, the corresponding exchange rate *k*_ex,12_, which describes the Mg^2+^-induced stabilization of IBS1*, increases at the expense of the dissociation rate *k*_off,10_ over the course of the Mg^2+^ titration (Supplementary Fig. 11). In the RNA–DNA hybrid, on the other hand, the off-rate outweighs the exchange between different EBS1*/dIBS1* conformations, thereby attenuating the degree of heterogeneity in dIBS1* unbinding.

**Table 1:** Kinetic parameters determined with a hidden Markov model on dynamic single molecules.

Exon	Metal ions	*k*’_on,01_ (10^−2^ s^−1^)^a^	*k*_on,01_ (10^5^ M^−1^ s^−1^)^b^	*k*_off,10_ (s^−1^)	*k*_12_ (10^−2^ s^−1^)	*k*_21_ (10^−2^ s^−1^)	*K*_d_ (nM)^c^	Δ*G*_bind_ (kJ mol^−1^)	Δ*G*^‡^ (kJ mol^−1^)
IBS1*	1 M K^+ d^	2.96 ± 0.04	8.45 ± 0.11	0.051 ± 0.001	–	–	59.0	−41.2	39.2 ± 0.1
IBS1*	20 mM Mg^2+^	2.14 ± 0.07	6.11 ± 0.20	0.146 ± 0.006	33.1 ± 1.4	2.56 ± 0.10	15.2	−44.6	40.0 ± 0.1
dIBS1*	1 M K^+ d^	0.86 ± 0.03	1.72 ± 0.06	3.52 ± 0.11	–	–	17,500	−27.2	43.1 ± 0.1
dIBS1*	20 mM Mg^2+^	7.07 ± 0.03	14.1 ± 0.1	0.511 ± 0.002	2.35 ± 0.07	6.33 ± 0.16	255	−37.6	37.9 ± 0.1

Errors are standard deviations computed from likelihood ratio tests^76^

^a^Pseudo-first-order association rate coefficients, *k*′_on,01_, are extracted from the HMM

^b^Second-order rate coefficients, *k*_on,01_, are calculated from the pseudo-first-order association rate coefficient and the total IBS1* concentration in solution (35 nM IBS1* or 50 nM dIBS1*). For IBS1* this is *k*_on,01_ = *k*′_on_*× c*_IBS1*_^−1^

^c^Dissociation constants are calculated from the relative state population *S*_bound_ and *S*_unbound_ and the ligand concentration. For IBS1* the *K*_d_ is thus given by *K*_d_ = *S*_unbound_ × *c*_IBS1*_ × *S*_bound_^−1^

^d^EDTA (100 µM) are added to chelate any traces of divalent metal ions

### Sugar puckering increases dynamics at the RNA–DNA interface

What is the molecular origin of the lower stability of the RNA–DNA contact and the reduction in kinetic heterogeneity? Structurally RNA and DNA are set apart by a methyl group on thymine and the lack of a 2′-OH on the DNA sugar. Differences in duplex stability have been attributed to both features^46^. The 2′-hydroxyl group contributes to the structural diversity of RNA by forming hydrogen bonds that make up tertiary structure elements such as the ribose zipper or the A-minor motif^47,48^. In helical regions, the puckering mode of the sugar is critically influenced by the presence or absence of the 2′-OH group. The most common helical topologies in nature are the A and B forms. While DNA mostly adopts the B-form (C2′-endo), RNA prefers the A-form (C3′-endo), in which the 2′-OH is in a sterically and electronically more favorable axial position (Fig. 4b)^49^. The type of sugar pucker can be inferred from TOCSY experiments, where ^3^*J* coupling between H1′ and H2′ protons is related to the dihedral angle via the Karplus equation^49,50^. Coupling between the two nuclei is strong if the sugar is in a C2′-endo conformation with the protons in axial alignment. A switch to C3′-endo rotates the protons into an equatorial position where they are oriented perpendicularly to each other and coupling is weak^34,35^. Puckering dynamics have been observed on different timescales from picoseconds up to milliseconds^51–53^.

The seven nucleotides of IBS1* and their base-paired complements on the EBS1* hairpin show no crosspeaks between H1′ and H2′ as opposed to the unpaired loop residues A10, U11, and U12, which display a sharp signal (Supplementary Fig. 15). In previous NMR structure calculations, the torsion angles have therefore been restrained to either a C3′-endo (IBS1* and paired nucleotides of EBS1*) or a C2′-endo (A10, U11, and U12) conformation^34,35^. In dIBS1*, correlations between H1′ and H2′/H2″ are observable for all seven residues, yet the linewidths of these peaks are broader compared to U11 and U12. Quantification of the peak volumes yields intermediary values, suggesting a fast exchange between puckering modes on the NMR timescale. Consequently, no restraints have been applied to these residues and the resulting lowest energy NMR structures show puckers from C2′-exo to C2′-endo (Fig. 4c).

To assess whether the intermediary ring puckers (C4′-exo, O4′-endo, C1′-exo) are a result of interconverting conformations averaged out in the NMR measurement, we run molecular dynamics simulations for both tertiary contacts in their docked form. We first simulated the two contacts in the presence of K^+^ only, and in a second set of runs, we added Mg^2+^ at the positions expected from NMR chemical shifts, NOE restraints, and electrostatic calculations (Supplementary Fig. 16). In both cases, the ribose of the IBS1* residues is predominantly in the canonical C3′-endo conformation as expected from the absence of IBS1* crosspeaks in the TOCSY spectrum (Fig. 4c and Supplementary Figs. 17–20). Excursions to a C2′-endo occur mostly at the flexible 3′-terminus (C65). Overall, the canonical A-form helix of EBS1*/IBS1* is relatively rigid^49,52^. In contrast, residues in dIBS1* undergo frequent repuckerings with correlation times in the mid picosecond range both in the presence and absence of Mg^2+^ (Supplementary Figs. 17 and 19). Their pucker profiles are very broad and cover most of the Eastern half of the pseudorotation cycle. The simulations show no significant energy barrier separating north and south puckers and thus a variety of intermediary deoxyribose conformations are explored (Supplementary Figs. 18 and 20). Interestingly, the unpaired loop residues A10-U12 of the EBS1* hairpin also switch between C2′-endo and C3′-endo conformations, but on a timescale of several nanoseconds (Supplementary Figs. 21 and 22). Unlike the residues in dIBS1*, intermediary puckers in A10-U12 are unfavored due to the presence of a distinct energy barrier.

Because the dihedral angles of the sugar ring are all interdependent, the pucker dynamics propagate onto the backbone where they alter the distance between neighboring phosphates and the shape of the helix^49^. As a result, the RNA/DNA hybrid adopts neither a pure A-form nor a B-form geometry^34,54,55^. The conformational exchange at the RNA–DNA interface probably weakens the binding, thus contributing to the lower stability of hybrid contacts in general and in our case to faster dissociation rates of dIBS1*^37,38^.

## Discussion

Group II introns have developed a highly specific recognition mechanism to cut and paste themselves from one part of the genome to another. Each of the three classes, IIA, IIB, and IIC, relies on a slightly different set of tertiary interactions to position the target strand in the active site for cleavage^1,2^. However, common to all is the EBS1, which forms a duplex directly adjacent to the 5′-splice site. Here, we have investigated the thermodynamics and kinetics governing the stability of this tertiary contact using single-molecule FRET in combination with molecular dynamic simulations.

**Fig. 5:**
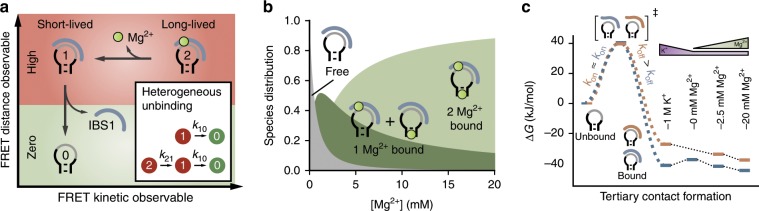
Tertiary contact formation monitored by FRET in space and time. **a** Exon release is described by two FRET observables: dye distance and state dwell times. Intron–exon dissociation can occur from state 1 (Mg^2+^ free) or state 2 (Mg^2+^ bound) and is thus kinetically heterogeneous. **b** Occupation of Mg^2+^ binding sites as a function of the ion concentration using Mg^2+^ binding constants from NMR chemical shift perturbations (Eq. 5)^15^. Cartoons depict possible species with no (gray), one (dark green), or two Mg^2+^ bound (light green). The Mg^2+^ free species corresponds to the high FRET state 1 in **a**, while the other species together make up state 2. Diffuse Mg^2+^ ions are omitted for clarity. **c** Free energy diagram of tertiary contact formation featuring a reactant-like, unstructured transition state. Off-rates determine the stability of the interactions. Dotted black lines connect levels of the bound state at different metal ion concentrations. Source data are provided as a Source Data file.

Our FRET trajectories capture binding and unbinding events across various K^+^ and Mg^2+^ concentrations. Consistent with previous experiments, IBS1 (un)docking and Mg^2+^ coordination are coupled, resulting in Mg^2+^ exchange rates on a timescale of milliseconds to seconds, thus accessible to camera-based detection^15,16^. We find that Mg^2+^ association and dissociation from the EBS1*/IBS1* contact are linked to very subtle conformational changes within the duplex, indiscernible by FRET. As a spectroscopic ruler, the FRET efficiency fails to resolve these kinetic states. In the time dimension, however, the states can be distinguished with regard to their dwell times (Fig. 5). Hence, exon unbinding is characterized by multiexponential decays from one degenerate FRET state, conceptually known as kinetic heterogeneity. Heterogeneity is common to many nucleic acid interactions and increases the complexity of the kinetic analysis^16^. Here, we have shown how to dissect such a state degeneracy and elucidate its molecular origin. For this purpose, we have drawn a state network of EBS1*/(d)IBS1* using a combination of dwell time analysis and hidden Markov modeling on an ensemble of FRET trajectories.

Based on our model system, we propose that the 5′-exon dissociates at different rates depending on whether the Mg^2+^ binding sites are occupied or not. While outer-sphere Mg^2+^ ions are typically thought to exchange on a millisecond timescale^56^, our previous single-molecule Mg^2+^ pulse experiments suggest that some ions remain associated with the RNA for seconds or even minutes^15^. These ions are probably chelated by the RNA^18^ and buried in a binding pocket formed upon docking of the 5′-exon^34^. The said cavity is shallower in the hybrid EBS1*/dIBS1* structure and Mg^2+^ binding to RNA–DNA contacts is generally weaker^35^. Yet, Mg^2+^ is required to stabilize the intrinsically labile RNA–DNA contact. Loss of a coordinated Mg^2+^ ion is therefore likely to be followed by immediate strand dissociation. Such a concerted mechanism of a Mg^2+^ and exon dissociation is consistent with the observed reduction in kinetic heterogeneity from IBS1* to dIBS1*.

How does ribozyme catalysis benefit from heterogeneity? Group II introns have evolved to find just the right balance of keeping a correct substrate in the active site long enough for the two transesterifications to occur, while releasing mismatched sequences before the first chemical step can take place^13^. To achieve such high selectivity, ribozymes make use of kinetic partitioning. Mg^2+^ prolongates the residence time of the exon given that there is a suitable binding site. Selective stabilization of the structurally and catalytically important exon–intron tertiary interaction forms the basis of its heterogeneous kinetics. A depletion of Mg^2+^ homogenizes the rates. Furthermore, splicing is downregulated, because K^+^ alone at physiological concentrations is neither able to stabilize the exon–intron contact sufficiently nor activate the phosphodiester bond for cleavage^11,57^.

In the cell, where Mg^2+^ is present in very low millimolar concentrations, Ca^2+^ is tightly regulated, and other divalent metal ions are generally scarce, intron-encoded or host supplied proteins are recruited to stabilize the weak interactions and promote catalysis. The group IIB intron Sc.*ai5γ* from which the EBS1*/IBS1* model interaction is derived, is a splicing-only intron, and resides in the housekeeping gene *COX1* of yeast mitochondria, where it is assisted by splicing factors such as the ATP-dependent helicase Mss116^58–60^. Mobile group II introns, in contrast, still have their own open reading frame encoding a maturase that stabilizes the exon–intron contact^61,62^. Only recently, cryo-EM structures of a maturase–intron complex from *Lactococcus lactis* and *Thermosynechococcus elongatus* have revealed how the exon is sandwiched between the protein’s thumb domain and EBS1/2^31,32^. This ternary complex between intron, exon, and protein might be of particular importance to convey stability to the labile RNA–DNA interaction during reverse splicing. Notably, the intron-encoded protein (IEP) has been found to bind more tightly to an intron lariat with the 5′-exon added in *trans* than to a spliced intron lacking the EBS/IBS interactions^61^. The exon–intron recognition complex and the IEP thus seem to cooperate to resolve instabilities in DNA target binding.

Our molecular simulations provide an atomic view on the exon–intron binding interaction and suggest that fast sugar puckering dynamics may be responsible for the lower affinity of the intron towards DNA exons. The simulations show that sugar puckers of dIBS1* are dispersed across the Eastern half of the pseudorotation cycle. In this energy basin the sugars are free to adopt different conformations. Does this switching have any regulatory function? On a microsecond to millisecond timescale, exchange between 3′-endo and 2′-endo sugars was found to be associated with helical to non-helical transitions, leading to alternative secondary structures, a feature common in riboswitches^51,63^. These rather slow puckering modes are further reminiscent of the *cis*/*trans* isomerization in peptide bonds containing proline residues, which were recently discovered to be a source of heterogeneity in intrinsically disordered protein interactions^64^. Unlike proline isomerization and secondary structure rearrangements, sugar puckering in dIBS1* happens on a timescale of pico- to nanoseconds and is thus much faster than exon release. Consequently, the intron is prone to let go of its DNA substrate unless the contact is properly stabilized by Mg^2+^ and/or a protein. We see this facile reversibility of EBS1/dIBS1 binding as a sort of quality control mechanism that ensures correct integration site selection as the ribonucleoprotein (RNP) scans along the exon^65^.

To test this hypothesis, the EBS1/IBS1 interaction needs to be put back into the context of the full intron. Such large and architecturally complex RNAs and RNPs have long eluded a detailed single-molecule kinetic analysis mainly because the site-specific introduction of suitable reporters, like fluorophores, has proven to be challenging^66^. For instance, to probe the same reaction coordinate as in our EBS1*/(d)IBS1* model construct, a dye needs to be placed adjacent to EBS1, which is located around nucleotide position 330 in Sc.*ai5γ*. Making such a FRET construct would require multiple fragments to be ligated enzymatically. Recent advances in co- and post-transcriptional nucleic acid labeling now provide more flexibility in choosing dye positions, which are close to functionally important elements, and thus provide a basis for studying the kinetics of large RNA–protein assemblies on a single-molecule level^67,68^.

Here, we have used instead a minimal construct, which is chemically synthesized and covalently labeled, giving us a more straightforward access to studying its binding kinetics. We have shown that ion binding and sugar puckering are two interacting processes that shape kinetic heterogeneity. While site specifically coordinated Mg^2+^ ions delay strand dissociation, sugar switching effectively promotes it. Off-rates are therefore the determining factor for contact stability, in line with previous studies on RNA kissing loops and various kinds of RNA, DNA, and hybrid duplexes^39,69^. A question that remains is whether sugar puckering attenuates heterogeneity by directly interfering with metal ion coordination. To explore the relative binding affinities of Mg^2+^ to pure RNA and RNA–DNA hybrid contacts, MD simulations with enhanced sampling may prove useful^43,70^. Such simulation might also detect small structural changes induced upon Mg^2+^ coordination that FRET struggles to resolve. Altering the degree of residual dynamics at binding interfaces is a common type regulation in biomolecules. By balancing flexibility and rigidity, sugar puckering and metal ions control RNA dynamics and form the molecular basis of kinetic heterogeneity.

## Methods

### Construct design

Oligos were purchased from IBA Lifesciences (Göttingen, DE) or Microsynth (Balgach, Switzerland) with cyanine labels at the 5′ end (Cy3-EBS1* and Cy5-IBS1* or Cy5-dIBS1*) and a biotin at the 3′ end. Photophysical parameters (fluorescence lifetime, time-resolved anisotropy) of the labeled oligos have been previously characterized in detail^71^. Sequences are derived from the group IIB intron Sc.*ai5γ* and feature a slightly elongated stem as well as two A-to-C transversions in the loop. These were previously introduced at residues 15 and 17 to stabilize the exon–intron interaction (see Supplementary Methods). For surface immobilization on the microscopy slides, a single-stranded overhang was added that contains four uracils and a biotin at the 3′ terminus^15^. We refer to these mutated and labeled constructs as EBS1*/IBS1* (RNA–RNA) and EBS1*/dIBS1* (RNA–DNA) to differentiate them from the original sequence in the group II intron.

### Single-molecule FRET experiments and trace processing

RNA hairpins and their RNA or DNA complements were buffered in 50 mM 3-(*N*-morpholino)propanesulfonic acid (MOPS) at pH 6.9. The imaging solution further contained 100 mM KCl, 1% glucose (w/v), 1 mM Trolox, an oxygen-scavenging system (2170 U/mL catalase, 165 U/mL glucose oxidase), and varying amounts of MgCl_2_ (0–20 mM) or alternatively 1 M KCl with 100 µM EDTA to remove any traces of divalent ions. The biotinylated EBS1* RNA was immobilized on a quartz surface via a streptavidin linkage^72^. For this purpose, the microfluidic channel was first coated with biotinylated bovine serum albumin (5 min), treated with streptavidin (10 min), and finally incubated with biotinylated EBS1* RNA (10 pM, 5 min). IBS1* or dIBS1* strands were then flushed into the chamber at concentrations of 35 or 50 nM, respectively. Molecules were imaged on a custom-built TIRF microscope using a water-immersion objective (UPlanSApo 60×/1.2-W, Olympus) at a camera frame rate of 5 or 10 Hz (Andor iXon DU-897). Single molecules with anti-correlated donor/acceptor emission were selected and corrected for background and donor bleedthrough into the acceptor channel using the freely available software package MASH-FRET (https://github.com/RNA-FRETools/MASH-FRET.git)^16,73^. The FRET efficiency was then calculated from the corrected donor and acceptor intensities after donor excitation according to

1 $${\mathrm{FRET}} = \frac{{I_{\mathrm{A}}}}{{I_{\mathrm{A}} + I_{\mathrm{D}}}}.$$

FRET histograms were fitted to a Gaussian mixture model and uncertainties were estimated by bootstrapping^74^.

### Single-molecule kinetic analysis

Individual single-molecule traces were discretized based on a threshold criterion to generate sets of dwell times in the well-separated zero and high FRET state. A mean dwell time, 〈*t*_zero,*n*_〉 and 〈*t*_high,*n*_〉 was calculated for each trace *n*. On the other hand, cumulative dwell time histograms were computed by sorting dwell times from all dynamic molecules. Only molecules showing at least two transitions between the two states were considered as dynamic. Characteristic decay constants, *τ*_zero,m_ and *τ*_high,m_, were obtained by fitting a biexponential (Supplementary Eq. 2) or a stretched exponential (Supplementary Eq. 3) to the distribution. For the binding reaction, second-order rate coefficents, *k*_on_, were calculated by dividing the pseudo-first-order on-rate, *k*′_on_, by the total IBS1* or dIBS1* concentration in solution: *k*_on_ = *k*′_on_ × *c*_(d)IBS1*_^−1^_._ Dissociation constants, *K*_d_, were computed from the on- and off-rates for each decay component *m* or alternatively for each individual molecule *n*. In case of the latter, cumulative distributions of *K*_d,*n*_ were built and fitted to a normalized logistic function

2 $$P( {K_{{\mathrm{d}},n}} ) = \frac{1}{{1 + (K_{\mathrm{d}}/K_{{\mathrm{d}},n})^p}}.$$

Error estimates on all variables were computed from a set of 100 bootstrap samples. For a more detailed mathematical description of the kinetic analysis, we refer the reader to the Supplementary Information. The Gibbs free energy of the bound state with respect to the free hairpin was determined using

3 $$\Delta G = RT\ln \left( {K_{\mathrm{d}}} \right),$$

with the gas constant *R* and temperature *T*. The energy barrier separating the bound and unbound state was computed according to transition state theory as

4 $${\mathrm{\Delta }}G^\ddagger = - RT\, {\mathrm{ln}}\left( {\frac{{k_{{\mathrm{on}}}h}}{{\kappa k_{\mathrm{B}}T}}} \right),$$

with the binding rate *k*_on_, Planck’s constant *h*, and Boltzmann’s constant *k*_B._ Our calculations of ${\mathrm{\Delta }}G^\ddagger$ assume a transmission coefficient *κ* = 1, thus providing a lower limit for the difference in free energy between the reactants and the transition state^20,75^.

The fraction of Mg^2+-^ bound RNA *Θ* is computed using association constants, *K*_1_ and *K*_2_, derived from NMR chemical shift perturbations^15^

5 $$\Theta = \frac{{K_1\left[ {{\mathrm{Mg}}^{2 + }} \right] + K_2\left[ {{\mathrm{Mg}}^{2 + }} \right] + K_1K_2\left[ {{\mathrm{Mg}}^{2 + }} \right]^2}}{{1 + K_1\left[ {{\mathrm{Mg}}^{2 + }} \right] + K_2\left[ {{\mathrm{Mg}}^{2 + }} \right] + K_1K_2\left[ {{\mathrm{Mg}}^{2 + }} \right]^2}}.$$

### Global HMM and FRET trace simulation

For each salt condition, a HMM was first trained on a trace-by-trace basis as implemented in the software package SMACKS^45,76^. In a second step, the predetermined emission probabilities were fixed and the start and transition probabilities of a global HMM were optimized collectively on the entire ensemble of dynamic FRET traces. Kinetic models with different connectivities and degeneracies were tested and evaluated based on a BIC and biochemical significance (see Supplementary Discussion). To validate the obtained rate system, degenerate FRET traces were re-simulated in a subroutine of MASH-FRET^77^ from the underlying rate matrix

6 $${\mathbf{K}} = \left( {\begin{array}{*{20}{c}} { - k_{01}} & {k_{01}} & 0 \\ {k_{10}} & { - (k_{10} + k_{12})} & {k_{12}} \\ 0 & {k_{12}} & { - k_{12}} \end{array}} \right).$$

Dwell time histograms were built analogously to the experimental traces.

### Poisson–Boltzmann continuum electrostatics

The electrostatic surface potential of EBS1* alone and EBS1* in complex with either IBS1* or dIBS1* were computed with the APBS Electrostatics plugin^78^ for PyMOL. Partial charges and VdW radii were assigned with PDB2PQR^79^ using parameters from AMBER-ff99. Calculations were run at 300 K with 100 mM monovalent and 20 mM divalent ions.

### Molecular dynamics simulation

NMR structures of the tertiary contact formed between EBS1* and IBS1* or dIBS1* (PDB: 2M23 and 2M1V) were used as starting points for molecular dynamics simulations with GROMACS 5.1^80^. The simulations use the AMBER-ff99 force field^81^ with parmbsc0^82^ and χOL3^83,84^ corrections (corresponds to AMBER-ff14). Monovalent ions were modeled with parameters from Joung and Cheatham^85^, while those for Mg^2+^ were taken from Li et al.^86^. The molecules were solvated in a dodecahedral box filled with TIP3P water and randomly interspersed with K^+^ and Cl^−^ ions to neutralize the net charge and reach a concentration of about 100 mM. In a second set of simulations, Mg^2+^ ions were placed at specific locations suggested by NMR chemical shifts and NOE restraints to [Co(NH_3_)_6_]^3+^. The nucleic acids were then charge neutralized and the K^+^ and Mg^2+^ concentrations set to 100 mM and 20 mM, respectively (concentrations are estimates because of the limited box size). In an equilibration phase (2 × 5 ns, NVT and the NPT ensembles), the temperature was adjusted to 300 K using the velocity rescale thermostat and the pressure was kept constant at 1 bar by the Parrinello–Rahman barostat. Bonds were constrained by the LINCS algorithm with an integration time step of 2 fs. Nonbonded interactions use the Verlet scheme with a cut-off of 1.4 nm, while long-range electrostatics were treated with the particle mesh Ewald algorithm. Simulations of EBS1*/IBS1* and EBS1*/dIBS1* (both with and without Mg^2+^) were run for 2 μs each. Convergence was assessed by computing the root mean square deviation over the nucleic acids and time binned histograms of the sugar puckers, showing that the repuckering time is much shorter than the overall simulation length. The pseudorotation angles of the riboses were calculated with PLUMED 2.5.2^87^ and assigned to one of ten puckering modes (phase angle increments of 36°)^49^ according to the definition by Huang et al.^88^ (Supplementary Eqs. 15–18), which are related to the one introduced originally by Altona and Sundaralingam^89^. The spatial distribution of Mg^2+^ ions, starting from a set of experimentally observed positions, is visualized using a Gaussian kernel density estimate. The point cloud is normalized by the ion concentration, color coded by the density, and mapped onto the NMR structure (Supplementary Fig. 16).

### NMR linewidth analysis

Linewidths and peak volumes in [^1^H-^1^H]TOCSY spectra were calculated using NMRFAM-Sparky^90^ by fitting a set of two-dimensional Gaussians to the assigned peaks.

### Reporting summary

Further information on research design is available in the Nature Research Reporting Summary linked to this Article.

## Supplementary information

Supplementary Information

Peer Review File

Reporting Summary

## Data Availability

The data that support the findings of this study are available from the corresponding authors upon reasonable request. The source data underlying Figs. 1d–g, 2a–g, 3a, b, 4c, 5b, c and Supplementary Figs. 1, 2, 7, 9, 15, 17–22 are provided as a Source Data file.

## Source data

Source Data

## Code availability

A Matlab-based software package for video processing, evaluation of single-molecule trajectories, histogram, and dwell time distribution analysis is freely available at https://github.com/RNA-FRETools/MASH-FRET.git. Documentation of the entire workflow of MASH-FRET can be found at https://rna-fretools.github.io/MASH-FRET/.
