# Supplementary material for: Metal ions and sugar puckering balance single-molecule kinetic heterogeneity in RNA and DNA tertiary contacts
Source: Nat Commun. 2020 Jan 8;11:104. doi: 10.1038/s41467-019-13683-4 (PMC6949254; doi:10.1038/s41467-019-13683-4)
Supplement: Supplementary file 1 — Supplementary Information [file 41467_2019_13683_MOESM1_ESM.pdf]

SUPPLEMENTARY INFORMATION

Metal ions and sugar puckering balance single-molecule kinetic heterogeneity in RNA and DNA tertiary contacts

Steffen et al.

Author information

Fabio D. Steffen[1]*, Mokrane Khier[1]*, Danny Kowerko[1,2], Richard A. Cunha[1], Richard Börner[1,3†], Roland K.O. Sigel[1†]

[1] Department of Chemistry University of Zurich Winterthurerstrasse 190, 8057 Zurich, Switzerland

[2] Department of Informatics, TU Chemnitz, Straße der Nationen 62, 09111 Chemnitz, Germany

[3] current address: Laserinstitut Hochschule Mittweida, University of Applied Sciences Mittweida, Technikumplatz 17, 09648 Mittweida, Germany

* These authors contributed equally

† Corresponding authors: Richard Börner, richard.boerner@chem.uzh.ch, Roland K.O. Sigel roland.sigel@chem.uzh.ch

Table of Contents

Supplementary Methods 3

Construct design 3

Single-molecule dwell time analysis 3

Sugar pucker pseudorotation cycle 5

Supplementary Discussion 6

Coupling of tertiary contact formation and Mg^{2+} binding and selection of an appropriate kinetic model 6

Analytical solution of a 1:1 ligand-receptor interaction 7

Supplementary Tables 8

Supplementary Figures 11

Suppementary References 33

Supplementary Methods

Construct design

The exon and intron binding site 1 (EBS1/IBS1) sequences used herein are derived from the group IIB intron Sc.ai5γ. Compared to the wild type, the stem was elongated by four nucleotides and a 3'-overhang was introduced for surface immobilization (underlined residues). Furthermore, two point mutations in the loop were introduced to stabilize the tertiary interaction *in vitro* (bold residues) without affecting the cleavage rate.[1] A star is appended to the name in order to differentiate the constructs from their native counterpart in the group IIB intron.

EBS1* Cy3 5'-<u>GGAG</u>UAUGUAUUGG**C**A**C**UGAGCAUA<u>CUCCUUUU</u>-3' – biotin
IBS1* Cy5 – 5'-CA**G**U**G**UC-3'
dIBS1* Cy5 – 5'-CA**G**T**G**TC-3'

Single-molecule dwell time analysis

In the following we outline the procedure used to extract dwell times in the zero and high FRET state, their corresponding binding and unbinding rates as well as distributions of dissociation constants. The nomenclature used herein follows in most parts the one introduced by Kowerko *et al.,* PNAS (2015).[2]

Thermodynamic parameters such as dissociation constants, K_d, or binding free energies ΔG can be calculated directly from the relative occupation of the two FRET states in the FRET histogram and the total ligand concentration (**Supplementary Fig. 4a**). Below, we decided to use a shot noise free approach to get both kinetic and thermodynamic parameters from the state dwell times. For this purpose, single-molecule time traces were discretized using a thresholding criterion corresponding to the midpoint between the centers of the zero and high FRET distribution. Complementary cumulative dwell time distributions $C(t_{zero})$ and $C(t_{high})$ were computed by first sorting all dwell times of all molecules recorded under equal buffer conditions in ascending order and then calculating the relative occurrence of dwell time $i \in \{1,\ldots,i_{max,n}\}$ of molecule $n \in \{1,\ldots,n_{max}\}$. This gives a set of probabilities $p(t) = \{p_1,\ldots p_N\}$ for the unique dwell times $t=\{t_1\ldots t_N\}$ from which the complementary cumulative dwell time probability is computed as

$$C(t_j) = 1 - \sum_{l=1}^{j} p(t_l), \quad \text{for } j = 1,2,\ldots,N \tag{1}$$

where t_j is the jth element in the dwell time sets t_{zero} or t_{high}.

The dwell time distribution $C(t_{zero})$ and $C(t_{high})$ can be described as a series of exponentials, i.e. for the high FRET state

$$C(t_{\text{high}}) = \sum_{m=1}^{m_{\max}} a_{\text{high},m}\, e^{t_{\text{high}}/\tau_{\text{high},m}} \quad \text{with } \sum_{m=1}^{m_{\max}} a_{\text{high},m} = 1 \ . \tag{2}$$

Alternatively, a single-exponential is modified by a stretching factor β

$$C(t_{\text{zero}}) = e^{(t_{\text{zero}}/\tau_{\text{zero}})^{\beta}} \tag{3}$$

Binding k_{on} and unbinding rates k_{off} were calculated from time constants t_{zero} and t_{high} for each decay component m according to

$$k_{\text{on},m} = \frac{1}{\tau_{\text{zero},m}\, c_{\text{total}}(\text{IBS1*})} \tag{4}$$

$$k_{\text{off},m} = \frac{1}{\tau_{\text{high},m}} \tag{5}$$

Dissociation constants of the respective decay components $K_{d,m}$ were calculated from the on- and off-rates as follows

$$K_{d,m} = \frac{k_{off,m}}{k_{on,m}} = \frac{\tau_{zero,m}\, c_{total}(IBS1^*)}{\tau_{high}} \tag{6}$$

Dissociation constants may also be calculated for an individual molecule n by averaging over i_{max} and j_{max} dwell times in the zero and high FRET state respectively

$$K_{d,n} = \frac{\langle t_{zero,n}\rangle\, c_{total}(IBS1^*)}{\langle t_{high,n}\rangle} \tag{7}$$

$$\text{with } \langle t_{zero,n}\rangle = \frac{\sum_{i=1}^{i_{max}} t_{zero,i,n}}{i_{max,n}} \text{ and } \langle t_{high,n}\rangle = \frac{\sum_{j=1}^{j_{max}} t_{high,j,n}}{j_{max,n}} \tag{8}$$

The number of dwell times i_{max} and j_{max} can vary between different molecules and $|i_{max}-j_{max}| = 0$ or 1. In the limiting case where $i_{max,n} = j_{max,n} = 1$ the average dwell times $\langle t_{zero,n}\rangle$ and $\langle t_{high,n}\rangle$ would be exponentially distributed according to

$$p(\langle t_{zero,n}\rangle) = e^{-\langle t_{zero,n}\rangle/\tau_{zero}} \text{ and } p(\langle t_{high,n}\rangle) = e^{-\langle t_{high,n}\rangle/\tau_{high}} \tag{9}$$

yet for most molecules $\langle t_{zero,n}\rangle$ and $\langle t_{high,n}\rangle$ are averaged over $i_{max,n}$ and $j_{max,n} > 1$ dwell times which is described by a gamma distribution [3]

$$p_\Gamma(\langle t_{zero,i_{max},n}\rangle) = A\, \frac{k^{i_{max}} \langle t_{zero,i_{max},n}\rangle^{i_{max}-1}}{\Gamma(i_{max})}\, e^{-k\langle t_{zero,i_{max},n}\rangle} \tag{10}$$

with amplitude A and rate $k = \tau_{zero}^{-1}$. Γ is the gamma function and $i_{max,n}$, the number of dwell times over which is being averaged. The higher the number of dwell times per trace, the narrower the distribution will get.

Hence, the distribution of $K_{d,n}$ values can be expressed as a ratio of two gamma distributions

$$p(K_{d,n}) = \frac{p_\Gamma(\langle t_{zero,i_{max},n}\rangle) c_{total}(IBS1^*)}{p_\Gamma(\langle t_{high,j_{max},n}\rangle)} = A\, \frac{K_d^{-N}}{B(N,N)} \left(1 + \frac{K_{d,n}}{K_d}\right)^{-2N} K_{d,n}^{N-1} \tag{11}$$

with $N := i_{max} = j_{max}$, as described previously by Coelho and Mexia [4]. Here, $B(i_{max}, j_{max})$ refers to the beta function and A is an amplitude to account for the frequency of the $K_{d,n}$ values.

Expressing supplementary equation 11 in terms of $p(\log K_{d,n})$ gives

$$p(\log K_{d,n}) = A\, \frac{(10^{\log K_d})^{-N}}{B(N,N)} \left(1 + \frac{10^{\log K_{d,n}}}{10^{\log K_d}}\right)^{-2N} (10^{\log K_{d,n}})^{N-1} 10^{\log K_{d,n}} \ln(10) \tag{12}$$

In order to make the distribution independent of the bin size, cumulative distributions $P(\log K_{d,n})$ were built analogously to the cumulative dwell time distribution and fitted to a normalized logistic function

$$P(K_{d,n}) = \frac{1}{1 + (K_d/K_{d,n})^p} \tag{13}$$

$$P(\log K_{d,n}) = \frac{(10^{\log K_{d,n} - \log K_d})^p}{1 + (10^{\log K_{d,n} - \log K_d})^p} \tag{14}$$

The parameter, p describes the steepness of the cumulative distribution and is thus related to the number of dwell times N in supplementary equation 11/12.

Sugar pucker pseudorotation cycle

Pseudorotation angle and pucker amplitude were calculated according to Huang and York[5] and rely on two endocyclic torsion angles v_1 and v_3. The Cartesian coordinates

$$Z_x = \frac{v_1+v_3}{2\cos(4\pi/5)} \tag{15}$$

$$Z_y = \frac{v_1-v_3}{2\sin(4\pi/5)} \tag{16}$$

can be transformed into polar coordinates to give the pucker phase P and amplitude A

$$P = \arctan\left(\frac{Z_y}{Z_x}\right) \tag{17}$$

$$A = \sqrt{Z_x^2 + Z_y^2} \tag{18}$$

Rate matrix for FRET trace simulations

In a Markov chain, state transitions are treated as homogenous processes, and the respective $J \times J$ matrix **P** with transition probabilities p_{ij} is defined by the transition rates $k_{i\neq j}$ and the camera frame rate f as follows [6]

$$\mathbf{P} = \begin{pmatrix} e^{-\sum_{j=2}^{J}\frac{k_{1j}}{f}} & (1-p_{11})\frac{k_{12}}{\sum_{j=2}^{J}k_{1j}} & \cdots & (1-p_{11})\frac{k_{1j}}{\sum_{j=2}^{J}k_{1j}} \\ (1-p_{22})\frac{k_{21}}{\sum_{j\neq 2}^{J}k_{2j}} & e^{-\sum_{j\neq 2}^{J}\frac{k_{2j}}{f}} & & \\ \vdots & & \ddots & \vdots \\ (1-p_{JJ})\frac{k_{i1}}{\sum_{i\neq j}^{J}k_{ij}} & & \cdots & e^{-\sum_{i\neq j}^{J}\frac{k_{ij}}{f}} \end{pmatrix} \tag{19}$$

where $p_{i=j}$ is the probability to stay within the same state and $p_{i\neq j}$ the probability to transit from state i to state j, respectively. Rates are assumed to adopt values ≥ 0, thus the probabilities are within $1 \geq p_{i,j} \geq 0$. With the integration time of the camera $\Delta t = f^{-1}$, the first order Taylor-series approximation $T_1\, p_{i=j}(\Delta t; 0) = p(0) + p'(0)\Delta t = 1 - \sum_{i\neq j}^{J} k_{ij}\, \Delta t$ yields the commonly used transition probability matrix for $k < f$

$$\mathbf{P} = \begin{pmatrix} 1-\sum_{j=2}^{J}k_{1j}\,\Delta t & k_{12}\Delta t & \cdots & k_{1j}\Delta t \\ k_{21}\Delta t & 1-\sum_{j\neq 2}^{J}k_{2j}\,\Delta t & & \\ \vdots & & \ddots & \vdots \\ k_{i1}\Delta t & & \cdots & 1-\sum_{i\neq j}^{J}k_{ij}\Delta t \end{pmatrix}. \tag{20}$$

The corresponding rate matrix is

$$\mathbf{K} = \begin{pmatrix} -\sum_{j=2}^{J}k_{1j} & k_{12} & \cdots & k_{1j} \\ k_{21} & -\sum_{j\neq 2}^{J}k_{2j} & & \\ \vdots & & \ddots & \vdots \\ k_{i1} & & \cdots & -\sum_{i\neq j}^{J}k_{ij} \end{pmatrix}. \tag{21}$$

Supplementary Discussion

Coupling of tertiary contact formation and Mg^{2+} binding and selection of an appropriate kinetic model

Complex formation between a receptor (EBS1*) and a ligand (IBS1* or dIBS1*) is linked to Mg^{2+} binding in a thermodynamic cycle (**Supplementary Fig. 4b**).[7] It consists of four states, where the receptor exists in either unbound (U) or native (N) form in the absence or presence of Mg^{2+}. Historically, the coupling of Mg^{2+} binding and folding has often been described by the following chemical equilibrium

$$\mathrm{U} + m\,\mathrm{Mg}^{2+} \leftrightarrow \mathrm{N}\bullet(\mathrm{Mg}^{2+})_{\mathrm{m}}$$

where m refers to the number of discrete Mg^{2+} ions associated with the native state. The associated equilibrium constant is given by

$$K_{\mathrm{eq}} = \frac{\left[\mathrm{N}\bullet(\mathrm{Mg}^{2+})_m\right]}{[\mathrm{U}][\mathrm{Mg}^{2+}]^m} \tag{22}$$

which translates into the well-known Hill equation

$$\theta = \frac{[\mathrm{Mg}^{2+}]^m}{K_{\mathrm{eq}} + [\mathrm{Mg}^{2+}]^m} \tag{23}$$

where θ is the fraction of native EBS1*/IBS1* with bound Mg^{2+}. The limitations of the Hill model in describing Mg^{2+} assisted RNA folding have been widely discussed in the literature.[7–10] The model presumes that the native state has m more Mg^{2+} ions associated than the unbound one. However, charge neutrality is not satisfied by just a few site-bound Mg^{2+}. Instead, the overwhelming majority of Mg^{2+} are delocalized and remain hydrated in the ion atmosphere. Poisson-Boltzmann theory has proven useful to describe the electrostatic interactions of the ion atmosphere with a charged polyelectrolyte, like RNA or DNA. The software package APBS Electrostatics[11] implements Poisson-Boltzmann theory and was used to compute the electrostatic surface potential of the EBS1* hairpin alone and in complex with IBS1* or dIBS1* (**Fig. 4b**). A patch of negative surface potential, representing a possible Mg^{2+} interaction site, is only exposed when IBS1* or dIBS1* bind to the hairpin (state N in the thermodynamic cycle, **Supplementary Fig. 4b**). The lack of such a pocket in the unbound hairpin makes a specific coordination of Mg^{2+} at those residues unlikely in the ligand free state (U). We thus excluded the $\mathrm{U}\bullet(\mathrm{Mg}^{2+})_{\mathrm{m}}$ in the thermodynamic cycle from our kinetic model. Thus, the transition from the free EBS1* hairpin to a stable tertiary contact involves first the binding of IBS1* or dIBS1* and secondly the coordination of a Mg^{2+} ion. Conversely, the reverse reaction implies release of the ion followed by undocking of the complement. In EBS1*/dIBS1* these two processes are likely concerted. Such biochemical knowledge about the system under study helps to find an appropriate kinetic model and can corroborate a purely statistical criterion such as the Bayesian information criterion (BIC, **Supplementary Fig. 12**). Originally, we tested different kinetic networks: two-state (0-1), cyclic three state (-0-0-1-, -0-1-1-), linear three-state (0-1-1, the one selected) as well as a cyclic four-state model: (-0-0-1-1-). In this syntax, the value 0 corresponds to the zero FRET state and the value 1 to the high FRET state. Degeneracy is implied if the same digit occurs more than once. Note that in the linear three state model the degenerate states are a priori not identical because they differ in the number of connections (**Supplementary Fig. 12**). After training the HMM, the linear three-state model was validated again by re-simulated degenerate FRET traces from the optimized rate system using MASH-FRET (see Supplementary Methods).[6] Dwell time histograms were built in the same way as in the experiment and then compared to the experimental ones (**Supplementary Fig. 13** and **Supplementary Fig. 14**). Overall, the kinetic rates, k'_{on} and $k_{\mathrm{off},1/2}$, are in very good agreement between simulated and experimental decays, thus the HMM is able to disentangle the kinetic heterogeneity induced by divalent metal ions (**Supplementary Table 5**).

The contribution of magnesium binding to contact formation, $\Delta\Delta G_{Mg}$, can be mathematically expressed as

$$\Delta\Delta G_{\mathrm{Mg}} = \Delta G_{\mathrm{Mg}}^{\mathrm{N}} - \Delta G_{\mathrm{Mg}}^{\mathrm{U}} = (\Delta G_{\mathrm{D}}^{\mathrm{N}} + \Delta G_{\mathrm{S}}^{\mathrm{N}}) - (\Delta G_{\mathrm{D}}^{\mathrm{U}} + \Delta G_{\mathrm{S}}^{\mathrm{U}}) \quad (24)$$

where the subscript D and S refer to the diffuse and site binding free energy terms to either the native (N) or unfolded (U) state.[7] Since site-binding to the loop region of the free hairpin (U) was not observed by NMR chemical shifts[12] and a discrete binding pocket is absent (see above), the term ΔG_S^U is omitted and $\Delta\Delta G_{Mg}$ reduces to

$$\Delta\Delta G_{\mathrm{Mg}} = \Delta\Delta G_{\mathrm{D}} + \Delta G_{\mathrm{S}}^{\mathrm{N}} \quad (25)$$

where $\Delta\Delta G_D$ is the difference in diffuse binding to the N and U state.

Analytical solution of a 1:1 ligand-receptor interaction

Binding of a ligand L to a single receptor site R can be described by the degree of receptor saturation θ as

$$\theta = \frac{[\mathrm{L}]}{K_{\mathrm{d}} + [\mathrm{L}]} = \frac{[\mathrm{L_{tot}}]-[\mathrm{RL}]}{K_{\mathrm{d}}+[\mathrm{L_{tot}}]-[\mathrm{RL}]} \quad (26)$$

with

$$[\mathrm{RL}] = \frac{(K_{\mathrm{d}}+[\mathrm{L_{tot}}]+[\mathrm{R_{tot}}]) \pm \sqrt{(K_{\mathrm{d}}+[\mathrm{L_{tot}}]+\mathrm{R_{tot}})^2 - 4[\mathrm{L_{tot}}][\mathrm{R_{tot}}]}}{2}\ , \ [\mathrm{RL}] > 0\ . \quad (27)$$

Hence, the receptor saturation can be expressed in terms of the total ligand concentration $[L_{tot}]$, total receptor concentration $[R_{tot}]$, and the dissociation constant K_d. Binding isotherms for different combinations of these three parameters, depending on the experiment and system under study are displayed in **Supplementary Fig. 3**. The curves illustrate common restraints imposed by the experimental setup. In the smFRET experiments the amount of fluorescently labeled and freely diffusing ligand that can be added is limited because of background issues in the acceptor channel (direct excitation of Cy5 by the green laser is on the order of 5%). Consequently, the fraction of receptor saturation is low in the case of EBS1*/dIBS1*. In the NMR experiment, on the other hand millimolar amounts of receptor and ligand are required to achieve good signal to noise ratios. These concentrations are much higher than the expected K_d of the interaction $[L_{tot}] \approx [R_{tot}] >> K_d$. The resulting binding curve shows a sharp kink where $R_{tot} = L_{tot}$ and thus a small excess of ligand is sufficient to quantitatively saturate the receptor.

Supplementary Tables

Supplementary Table 1 Kinetic parameters from mean dwell time analysis of single-molecule FRET experiments.

cognate strand	metal ion concentration	center of $\langle k_{on} \rangle$ distribution (10^5 M^{-1} s^{-1}) [a]	center of $\langle k_{off} \rangle$ distribution (s^{-1})	K_d [b] (nM)	ΔG_{bind} [b] (kJ mol^{-1})	$\langle \Delta G^{\ddagger} \rangle$ [c] (kJ mol^{-1})
IBS1*	1 M K^+ [d]	8.8	0.052	59 ± 3	-41.3 ± 0.1	39.1
	0 mM Mg^{2+}	3.4	0.047	285 ± 113	-37.3 ± 1.0	41.4
	0.5 mM Mg^{2+}	4.5	0.031	83 ± 22	-40.4 ± 0.7	40.7
	2.5 mM Mg^{2+}	4.1	0.025	117 ± 23	-39.6 ± 0.5	41.0
	5 mM Mg^{2+}	4.9	0.021	89 ± 19	-40.2 ± 0.5	40.5
	10 mM Mg^{2+}	7.4	0.021	52 ± 12	-41.4 ± 0.6	39.5
	15 mM Mg^{2+}	5.6	0.017	42 ± 11	-42.2 ± 0.6	40.2
	20 mM Mg^{2+}	7.6	0.017	29 ± 8	-43.0 ± 0.7	39.4
dIBS1*	1 M K^+ [d]	2.3	2.6	8530 ± 1930	-28.9 ± 0.1	42.4
	2.5 mM Mg^{2+}	3.6	0.67	1630 ± 130	-33.0 ± 0.2	41.3
	5 mM Mg^{2+}	4.8	0.42	1110 ± 40	-34.0 ± 0.1	40.6
	10 mM Mg^{2+}	7.8	0.32	589 ± 18	-35.5 ± 0.1	39.4
	15 mM Mg^{2+}	9.2	0.33	447 ± 17	-36.2 ± 0.1	39.0
	20 mM Mg^{2+}	12	0.32	353 ± 8	-36.8 ± 0.1	38.3

Errors are standard deviations of 100 bootstrap samples.[13] The center of the point cloud $\langle k_{on} \rangle$ versus $\langle k_{off} \rangle$ is determined by k-means clustering.

All experiments were conducted with a monovalent ionic background of 100 mM KCl unless stated otherwise.

[a] Second-order mean rate coefficients, $\langle k_{on} \rangle$, are calculated from the pseudo-first-order mean association rate coefficient and the total IBS1* concentration in solution (35 nM IBS1* or 50 nM dIBS1*). For IBS1* this is $\langle k_{on} \rangle = \langle k'_{on} \cdot c_{IBS1*}^{-1} \rangle$. The tabulated values refer to the center of the point cloud of $\langle k_{on} \rangle$ versus $\langle k_{off} \rangle$.

[b] Dissociation constants, K_d, are calculated from the logistic fits (Eqn. S13) to the cumulative distribution of $K_{d,n}$ values. Binding free energies, ΔG_{bind}, are computed from the K_d.

[c] The free energy difference between the unbound and the transition state is calculated using the center of the distribution of mean association rates $\langle k_{on} \rangle$.

Supplementary Table 2 Comparison of single-molecule kinetics (sm, from mean dwell times, Supplementary Table **1**) with surface plasmon resonance (SPR) experiments (at 25°C).[14]

cognate strand	metal ion concentration	center of $\langle k_{on} \rangle$ distribution (10^5 M^{-1} s^{-1}, sm) [a]	k_{on} (10^5 M^{-1} s^{-1}, SPR) [a]	center of $\langle k_{off} \rangle$ distribution (s^{-1}, sm)	k_{off} (s^{-1}, SPR)	K_d (nM, sm) [c]	K_d (nM, SPR) [b]
IBS1*	0 mM Mg^{2+}	3.4	1.0 ± 0.2	0.047	0.015 ± 0.010	285 ± 113	150 ± 30
	5 mM Mg^{2+}	4.9	2.1 ± 1.3	0.021	0.006 ± 0.001	89 ± 19	30 ± 10
	0 mM Mg^{2+}	–	0.5 ± 0.2	–	1.45 ± 0.35	–	29000 ± 5700
	5 mM Mg^{2+}	4.8	2.4	0.42	0.18	1110 ± 40	720

Errors of single-molecule experiments (if specified) are standard deviations of 100 bootstrap samples.[13] Errors of SPR experiments (if specified) are standard deviations of independent measurements on two sensor chips. Experiments were conducted with a monovalent ionic background of 100 mM KCl.

[a] Second-order rate coefficients, $k_{on,01}$, are calculated from the pseudo-first-order association rate coefficient and the total IBS1* concentration in solution. For IBS1* this is $k_{on,01} = k'_{on} \cdot c_{IBS1*}^{-1}$.

[b] Dissociation constants, K_d, are calculated from the logistic fits (Eqn. S13) to the cumulative distribution of $K_{d,n}$ values (single-molecule) of directly from the rates $K_d = k_{off} \cdot k_{on}^{-1}$ (SPR).

Supplementary Table 3 Kinetic parameters from cumulative dwell time histogram analysis of single-molecule FRET experiments.

cognate strand	metal ion concentration	k_{on} (10^5 M^{-1} s^{-1}) [a]	β	a_1	$k_{off,1}$ (s^{-1})	$k_{off,2}$ (s^{-1})	$K_{d,1}$ (nM)	$K_{d,2}$ (nM)	$\Delta G_{bind,1}$ (kJ mol^{-1})	$\Delta G_{bind,2}$ (kJ mol^{-1})	$\Delta G^{‡}$ (kJ mol^{-1})
IBS1*	1 M K^+ [b]	8.84 ± 0.22	0.99 ± 0.01	0.10 ± 0.02	0.054 ± 0.002	–	1090 ± 320	–	-34 ± 0.7	–	39 ± 0.1
	0 mM Mg^{2+}	3.97 ± 0.64	1.00 ± 0.01	0.38 ± 0.08	1.1 ± 0.4	0.055 ± 0.011	2960 ± 113	143 ± 39	-32 ± 1	-39 ± 0.7	41 ± 0.4
	0.5 mM Mg^{2+}	4.85 ± 0.51	0.89 ± 0.07	0.43 ± 0.06	0.49 ± 0.12	0.021 ± 0.003	1020 ± 250	44 ± 7	-34 ± 0.6	-42 ± 0.4	41 ± 0.3
	2.5 mM Mg^{2+}	4.29 ± 0.35	0.95 ± 0.05	0.57 ± 0.05	0.31 ± 0.04	0.012 ± 0.002	738 ± 112	29 ± 4	-35 ± 0.4	-43 ± 0.4	41 ± 0.2
	5 mM Mg^{2+}	5.71 ± 0.61	0.84 ± 0.04	0.55 ± 0.03	0.35 ± 0.05	0.012 ± 0.002	619 ± 104	20 ± 4	-35 ± 0.4	-44 ± 0.4	40 ± 0.3
	10 mM Mg^{2+}	9.64 ± 0.82	0.88 ± 0.04	0.64 ± 0.04	0.28 ± 0.03	0.013 ± 0.002	5291 ± 40	13 ± 2	-37 ± 0.3	-45 ± 0.4	39 ± 0.2
	15 mM Mg^{2+}	7.20 ± 0.92	0.89 ± 0.07	0.48 ± 0.08	0.36 ± 0.12	0.012 ± 0.002	502 ± 163	17 ± 3	-36 ± 0.8	-44 ± 0.5	40 ± 0.3
	20 mM Mg^{2+}	8.06 ± 0.69	0.93 ± 0.07	0.39 ± 0.06	0.43 ± 0.13	0.012 ± 0.002	541 ± 159	16 ± 3	-36 ± 0.7	-45 ± 0.4	39 ± 0.2
dIBS1*	1 M K^+ [d]	3.79 ± 1.25	0.77 ± 0.10	1.00 ± 0.00	3.48 ± 0.32	–	10^4 ± 3200	–	-29 ± 0.8	–	41 ± 0.8
	2.5 mM Mg^{2+}	8.03 ± 0.66	0.71 ± 0.02	0.95 ± 0.02	0.80 ± 0.04	0.098 ± 0.030	1004 ± 104	122 ± 39	-34 ± 0.3	-39 ± 0.8	39 ± 0.2
	5 mM Mg^{2+}	8.76 ± 0.29	0.79 ± 0.01	0.94 ± 0.01	0.65 ± 0.01	0.071 ± 0.007	747 ± 27	82 ± 8	-35 ± 0.1	-40 ± 0.3	39 ± 0.1
	10 mM Mg^{2+}	10.6 ± 0.3	0.85 ± 0.01	0.94 ± 0.01	0.57 ± 0.01	0.064 ± 0.006	540 ± 16	60 ± 6	-36 ± 0.1	-41 ± 0.3	39 ± 0.1
	15 mM Mg^{2+}	12.4 ± 0.3	0.89 ± 0.01	0.95 ± 0.01	0.48 ± 0.01	0.059 ± 0.005	389 ± 13	48 ± 5	-37 ± 0.1	-42 ± 0.2	38 ± 0.1
	20 mM Mg^{2+}	14.8 ± 0.2	0.92 ± 0.01	0.96 ± 0.01	0.48 ± 0.01	0.051 ± 0.004	325 ± 6	35 ± 3	-37 ± 0.1	-43 ± 0.2	38 ± 0.1

Errors are standard deviations of 100 bootstrap samples.[13]
All experiments were conducted with a monovalent ionic background of 100 mM KCl unless stated otherwise.
[a] Second-order rate coefficients, $k_{on,1}$, are calculated from the pseudo-first-order association rate coefficient and the total IBS1* concentration in solution (35 nM IBS1* or 50 nM dIBS1*). For IBS1* this is $k_{on,1} = k'_{on,1} \cdot c_{IBS1*}^{-1}$.
[b] 100 µM EDTA are added to chelate any traces of divalent metal ions.

Supplementary Table 4 Kinetic parameters determined with a trained global hidden Markov model from single-molecule FRET experiments.

cognate strand	metal ion concentration	$k'_{on,01}$ (10^{-2} s^{-1}) [a]	$k_{on,01}$ (10^5 M^{-1} s^{-1}) [b]	$k_{off,10}$ (s^{-1})	k_{12} (10^{-2} s^{-1})	k_{21} (10^{-2} s^{-1})	K_d (nM) [c]	ΔG_{bind} (kJ mol^{-1})	$\Delta G^{‡}$ (kJ mol^{-1})
IBS1*	1 M K^+ [d]	2.96 ± 0.04	8.45 ± 0.11	0.051 ± 0.001	–	–	59.0	-41.2	39.2 ± 0.1
	0 mM Mg^{2+}	1.69 ± 0.07	4.82 ± 0.14	0.136 ± 0.010	–	–	281	-37.4	40.6 ± 0.1
	0.5 mM Mg^{2+}	1.08 ± 0.04	3.08 ± 0.10	0.351 ± 0.016	47.1 ± 2.4	4.28 ± 0.19	81.5	-40.4	41.7 ± 0.1
	2.5 mM Mg^{2+}	1.02 ± 0.03	1.92 ± 0.08	0.177 ± 0.007	15.0 ± 0.7	1.52 ± 0.06	46.0	-41.9	41.8 ± 0.1
	5 mM Mg^{2+}	1.44 ± 0.03	4.10 ± 0.09	0.179 ± 0.006	21.4 ± 0.7	1.70 ± 0.05	28.5	-43.0	41.0 ± 0.1
	10 mM Mg^{2+}	2.25 ± 0.05	6.44 ± 0.15	0.151 ± 0.005	15.2 ± 0.6	1.23 ± 0.04	17.2	-44.3	39.8 ± 0.1
	15 mM Mg^{2+}	2.05 ± 0.07	5.87 ± 0.19	0.118 ± 0.005	20.4 ± 1.0	2.21 ± 0.09	17.5	-44.3	40.1 ± 0.1
	20 mM Mg^{2+}	2.14 ± 0.07	6.11 ± 0.20	0.146 ± 0.006	33.1 ± 1.4	2.56 ± 0.10	15.2	-44.6	40.0 ± 0.1
dIBS1*	1 M K^+ [d]	0.86 ± 0.03	1.72 ± 0.06	3.52 ± 0.11	–	–	17490	-27.2	43.1 ± 0.1
	2.5 mM Mg^{2+}	1.87 ± 0.03	3.75 ± 0.06	0.740 ± 0.013	3.83 ± 0.35	6.68 ± 0.53	1190	-33.8	41.2 ± 0.1
	5 mM Mg^{2+}	3.47 ± 0.03	6.94 ± 0.05	0.619 ± 0.005	2.78 ± 0.14	5.76 ± 0.25	582	-35.6	39.7 ± 0.1
	10 mM Mg^{2+}	5.15 ± 0.03	10.3 ± 0.1	0.585 ± 0.004	2.40 ± 0.10	2.66 ± 0.11	306	-37.2	38.7 ± 0.1
	15 mM Mg^{2+}	7.01 ± 0.05	14.0 ± 0.1	0.508 ± 0.004	3.46 ± 0.13	7.85 ± 0.24	244	-37.7	37.9 ± 0.1
	20 mM Mg^{2+}	7.07 ± 0.03	14.1 ± 0.1	0.511 ± 0.002	2.35 ± 0.07	6.33 ± 0.16	255	-37.6	37.9 ± 0.1

Errors are standard deviations computed from likelihood ratio tests as detailed in [15].
All experiments were conducted with a monovalent ionic background of 100 mM KCl unless stated otherwise.
[a] Pseudo-first-order association rate coefficients, $k'_{on,01}$, are extracted from the HMM.
[b] Second-order rate coefficients, $k_{on,01}$, are calculated from the pseudo-first-order association rate coefficient and the total IBS1* concentration in solution (35 nM IBS1* or 50 nM dIBS1*). For IBS1* this is $k_{on,01} = k'_{on} \cdot c_{IBS1*}^{-1}$.
[c] Dissociation constants are calculated from the relative state population S_{bound} and $S_{unbound}$ and the ligand concentration. For IBS1* the K_d is given by $S_{unbound} \cdot c_{IBS1*} \cdot S_{bound}^{-1}$.
[d] 100 µM EDTA are added to chelate any traces of divalent metal ions.

Supplementary Table 5 Comparison of kinetic parameters determined from dwell time fits to the experimental decays (denoted as "exp.", Supplementary Table **3**) with the dwell time fits after re-simulating time traces from the rate system determined by the global hidden Markov model (denoted as "sim.").

cognate strand	metal ion concentration	k'_{on} (10^{-2} s^{-1}, exp.) [a]	k'_{on} (10^{-2} s^{-1}, sim.) [a]	$k_{off,1}$ (s^{-1}, exp.)	$k_{off,1}$ (s^{-1}, sim.)	$k_{off,2}$ (s^{-1}, exp.)	$k_{off,2}$ (s^{-1}, sim.)	a_1 (exp.)	a_1 (sim.)
IBS1*	1 M K^+ [b]	3.1 ± 0.1	3.1 ± 0.2	0.054 ± 0.002	0.056 ± 0.003	–	–	1	1
	20 mM Mg^{2+}	2.8 ± 0.2	3.0 ± 0.4	0.43 ± 0.13	0.19 ± 0.09	0.013 ± 0.002	0.014 ± 0.002	0.39 ± 0.06	0.22 ± 0.08
dIBS1*	1 M K^+ [b]	1.9 ± 0.6	1.4 ± 0.2	3.48 ± 0.32	3.33 ± 0.33	–	–	1	1
	20 mM Mg^{2+}	7.4 ± 0.1	7.4 ± 0.2	0.48 ± 0.01	0.52 ± 0.02	0.052 ± 0.004	0.018 ± 0.004	0.96 ± 0.01	0.96 ± 0.01

Errors are standard deviations of 100 bootstrap samples.[13]

Experiments at 20 mM Mg^{2+} were conducted with a monovalent ionic background of 100 mM KCl.

[a] Pseudo-first-order association rate coefficients, $k'_{on,01}$.

[b] 100 μM EDTA are added to chelate any traces of divalent metal ions.

Supplementary Figures

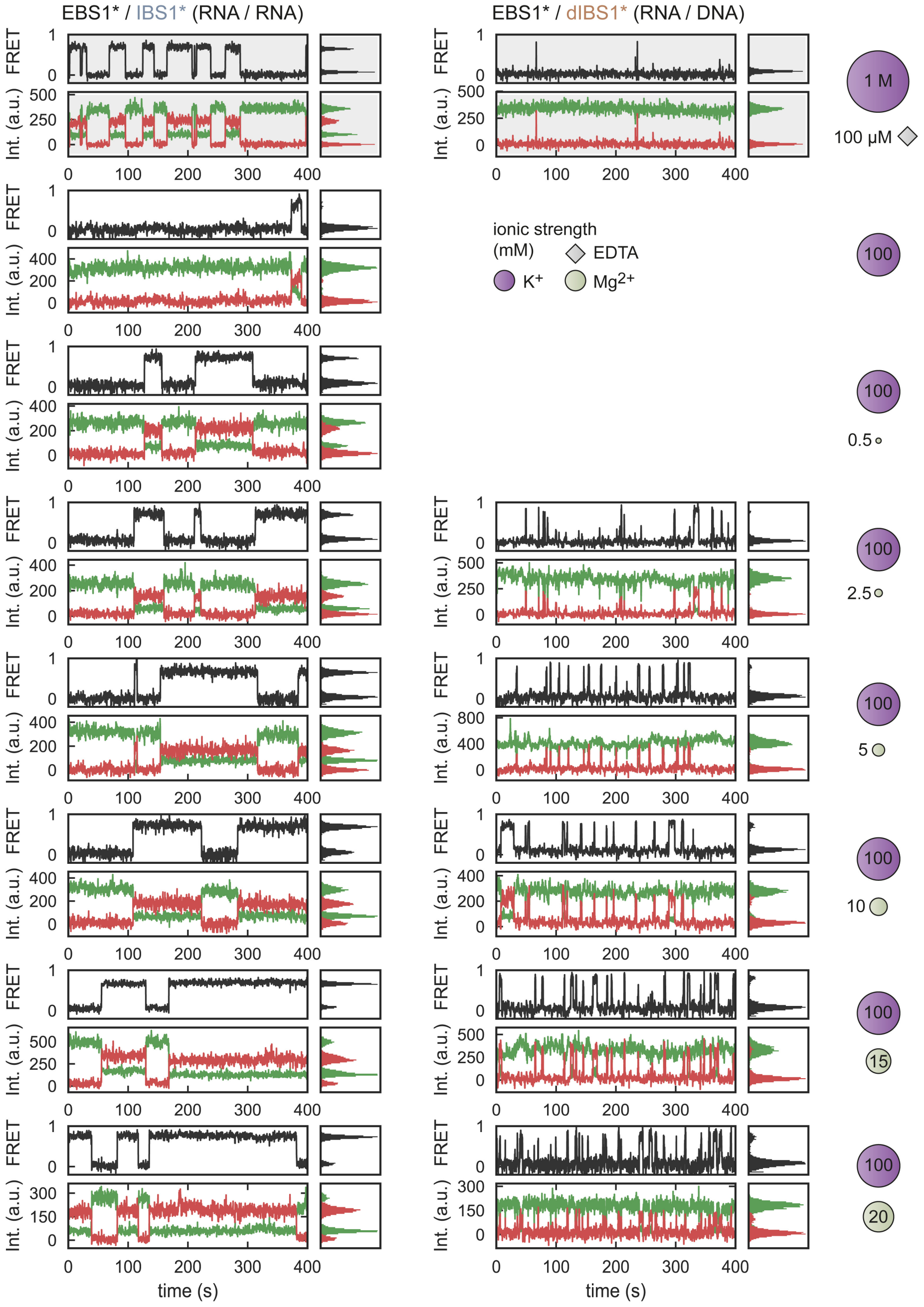

Supplementary Fig. 1 Dynamic single-molecule intensity and FRET time traces of EBS*/IBS1* (left) and EBS1/dIBS1* (right) at different Mg^{2+} concentrations. Anticorrelated Cy3 (green) and Cy5 (red) signals translate into FRET efficiencies that interconvert between a zero (unbound) and high (~0.75, bound) state. The traces are selected to reflect the thermodynamic state population in the overall histograms in Fig. 1f. Source data are provided as a Source Data file.

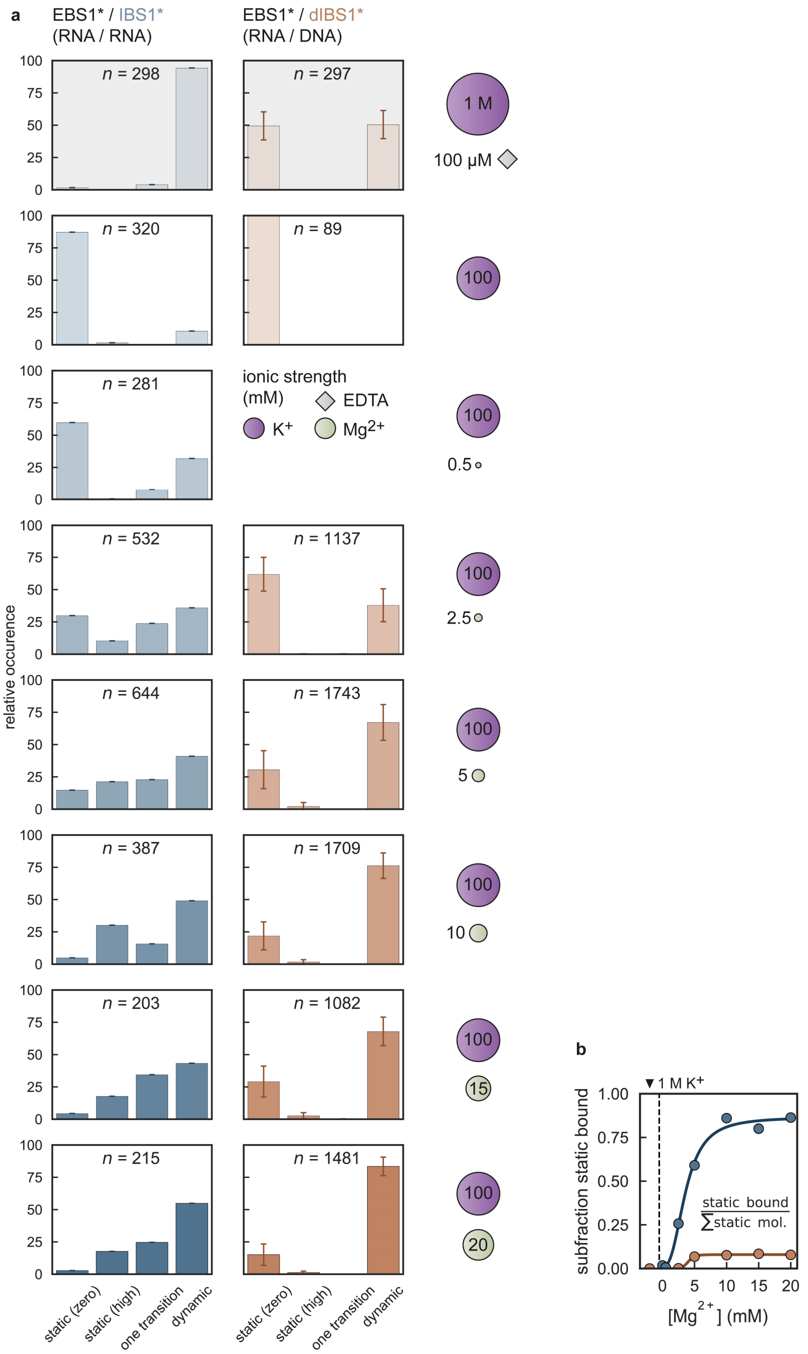

Supplementary Fig. 2 Classification of molecules according to their interconversion frequency between zero and high FRET states. (a) Bar charts depict relative abundance of static and dynamic molecules for EBS1*/IBS1* (blue) and EBS1*/dIBS1* (orange). Error bars are drawn from three independent experiments (for EBS1*/dIBS1* only) and correspond to mean ± s.d. The total number of molecules *n* is indicated for each condition. Mg^{2+} concentration is varied across the datasets (0-20 mM) under constant background of 100 mM KCl. High monovalent salt conditions (1 M K^+, 100 µM EDTA) are represented in the top row (gray background). (b) Subfraction of statically bound molecules relative to all static molecules across different Mg^{2+} concentrations. Source data are provided as a Source Data file.

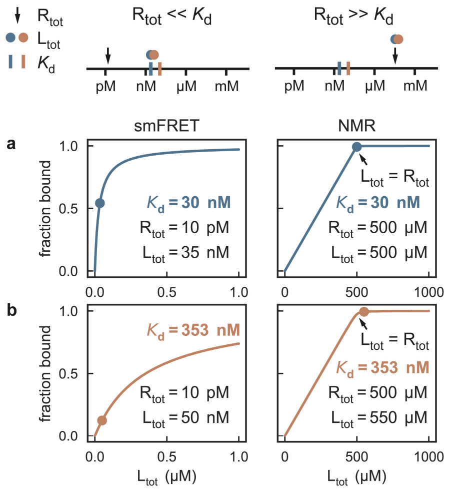

Supplementary Fig. 3 Binding isotherms for a 1:1 interaction between a receptor R and a ligand L. The curves represent the fraction of bound molecules as a function of the total ligand concentration as obtained from equation S26. The isotherm is computed for two sets of receptor concentrations: $[R_{tot}]$ = 10 pM (the concentration of immobilized EBS1* in the smFRET experiments) and $[R_{tot}]$ = 500 µM (the minimum concentration of EBS1* at which the NMR structures are solved). The dissociation constants characterizing the binding affinity of the ligand to the receptor are either (a) 30 nM (EBS1*/IBS1*, blue) or (b) 353 nM (EBS1*/dIBS1*, orange). The circles indicate the ligand concentration at which the experiment is recorded. In the single-molecule TIRF experiments the hairpin is present at concentrations far below the dissociation constant: $[R_{tot}] << K_d$. Due to background fluorescence the ligand concentration is limited to about 50 nM, which is not high enough to saturate the receptor. In the NMR tube $[R_{tot}] >> K_d$ and the isotherm shows a sharp kink where $[L_{tot}] = [R_{tot}]$ (black arrow). Here, at equimolar concentrations of ligand and receptor, EBS1* is fully saturated.

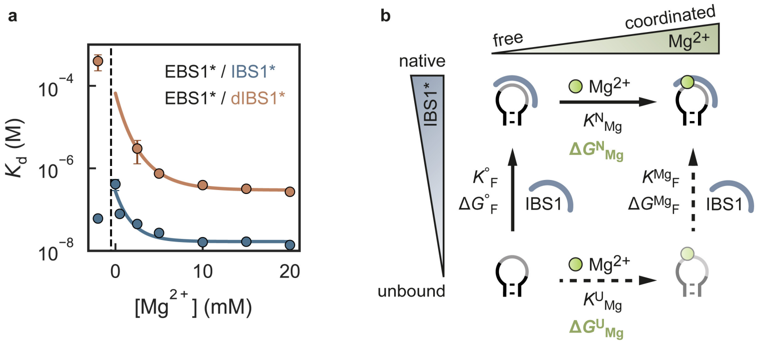

Supplementary Fig. 4 Thermodynamics of tertiary contact formation. (a) Dissociation constants of EBS1*/IBS1* (blue) and EBS1*/dIBS1* (orange) as a function of Mg^{2+} calculated from the relative population of the zero and high FRET state in the FRET histograms from dynamic molecules. The affinity of the dIBS1* towards the hairpin is more than one order of magnitude lower across all Mg^{2+} concentrations. Error bars are drawn as mean ± s.d. of 100 bootstrap samples. (b) Thermodynamic circle linking tertiary contact formation and Mg^{2+} binding. The transition from the unbound (U) to the native state (N) is described by the equilibrium constants $K^{\circ}{}_{F}$ in the absence and $K^{Mg}{}_{F}$ in the presence of Mg^{2+}. The free energy contribution of Mg^{2+} to tertiary contact formation is given $\Delta\Delta G_{Mg} = \Delta G^{N}{}_{Mg} - \Delta G^{U}{}_{Mg} = \Delta G^{Mg}{}_{F} - \Delta G^{\circ}{}_{F}$.[7] Binding of Mg^{2+} to the unbound state (U•Mg^{2+}, grayed out) is unlikely to be observed since a discrete binding pocket is only formed when IBS1* has bound to the EBS1* hairpin (Fig. 3b). This state and its connections are thus omitted in the HMM model and the thermodynamic cycle is transformed into a linear three-state model.

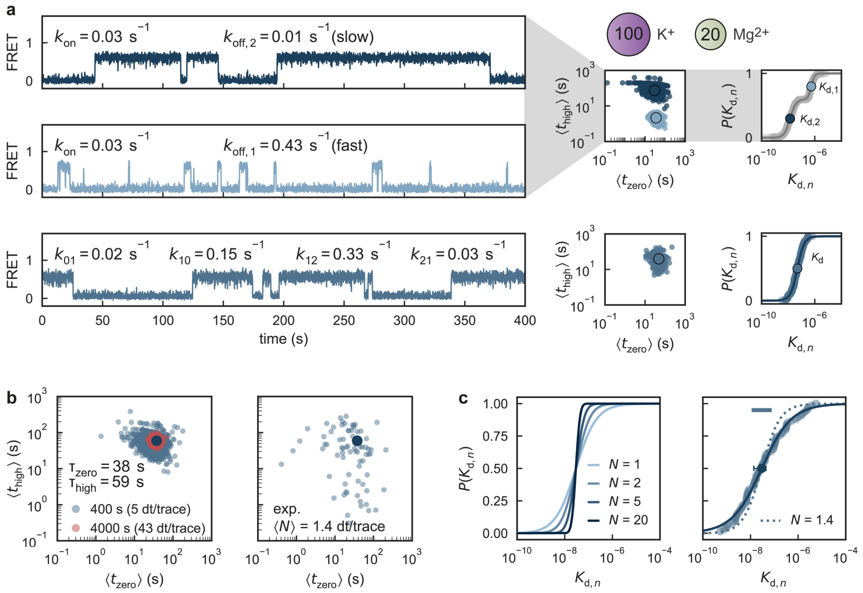

Supplementary Fig. 5 Simulation of slow exchange between kinetic regimes leading to inter-molecule heterogeneity. (a) Simulated homogenous two-state systems with a slow ($k_{off,2}$, top) or fast ($k_{off,2}$, middle) dissociation rate as obtained from dwell time analysis (Supplementary Table **3** and Supplementary Fig. **9**) of single-molecule FRET experiments at 100 mM KCl and 20 mM $MgCl_2$. Upon merging both simulations, the mean dwell-time plot split into two well separated clusters due to the separation of timescales of the simulated rates and because, by design, the two species do not interconvert. Thus, the cumulative distribution of all $K_{d,\underline{n}}$ shows two transitions one for each dissociation constant, $K_{d,1}$ and $K_{d,2}$. Conversely, if the rate system allows interconversion (k_{12} and k_{21}, bottom) between the kinetic regimes (see HMM model, Supplementary Table **4** and Supplementary Fig. **11**) the mean dwell time is averaged and only a single, stretched cluster remains. Here, short and long binding events occur in the same trace and the dissociation constant, K_d, is calculated from a single transition. (b) Effect of the average number of dwell times $\langle N \rangle$ per trace on the spread of the mean dwell time distribution. The number of dwell times N for an individual trace is computed as (i_{max} + j_{max})/2. A two-state system with the experimentally determined dwell time parameters τ_{zero} = 38 s and τ_{high} = 59 s is simulated with trace lengths of 400 s (blue, experimental observation time) and 4000 s (red) resulting in 5 or 43 dwell times per trace on average. For comparison, the experimental mean dwell time scatter plot is shown for EBS1*/IBS1 at 20 mM Mg^{2+}. (c) The average number of dwell times per trace affects the steepness of the $K_{d,n}$ distribution. $P(K_{d,n})$ is calculated as the numerical integral over equation S11 (i.e. the cumulative sum over the ratio of two gamma distributions) for different N=i_{max}=j_{max}. The experimental distribution is compared to the numerical integral for N=1.4.

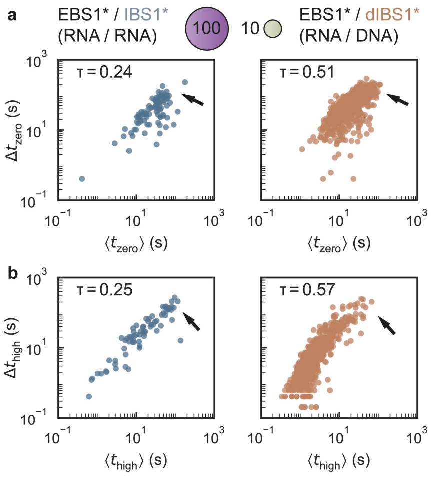

Supplementary Fig. 6 Correlation of mean dwell time $\langle t_{\text{high}} \rangle$ or $\langle t_{\text{zero}} \rangle$ with the difference between the shortest and longest dwell time Δt_{high} or Δt_{zero} for EBS1*/IBS1* (blue) and EBS1*/dIBS1* (orange). Kendall's tau coefficient is indicated for each plot as a measure for the correlation. The arrow points to the region of the most heterogeneous molecules (large Δt).

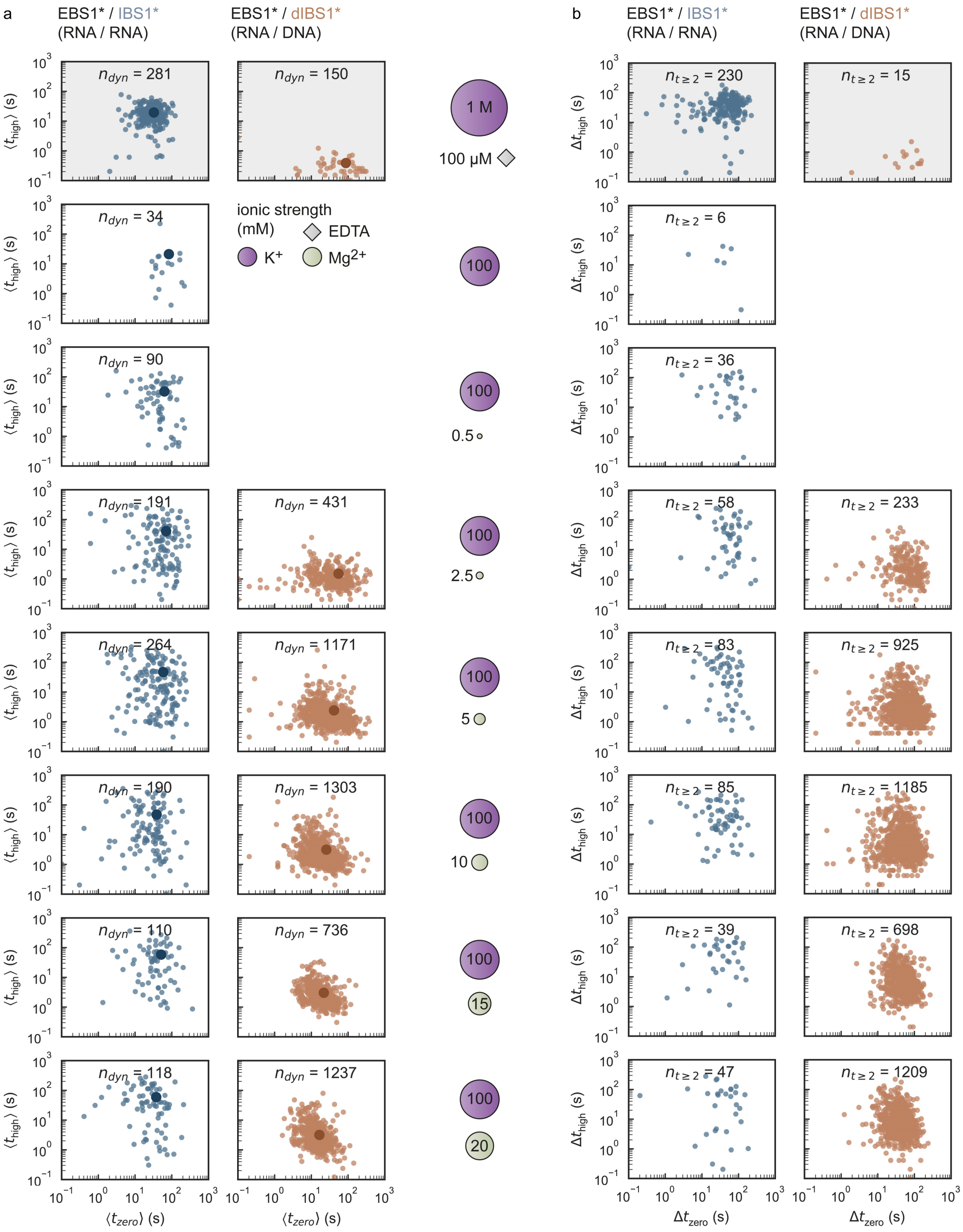

Supplementary Fig. 7 Experimentally characterized molecule-to-molecule variations in EBS1*/IBS1* (blue) and EBS1*/dIBS1* (orange). (a) Mean dwell time correlation plots of the bound, $\langle t_{\text{high}} \rangle$, and unbound state, $\langle t_{\text{zero}} \rangle$ across different Mg^{2+} concentrations. High monovalent salt conditions (1 M K^+, 100 µM EDTA) are represented in the top row (gray background). The cluster center drawn as a circle and the number of dynamic molecules n_{dyn} is indicated for each plot. (b) Difference between the shortest and longest dwell time within a trace for the bound Δ_{high} and unbound Δ_{zero} state. The number of dynamic molecules with at least two dwell times $n_{t\geq 2}$ are indicated for each plot. Source data are provided as a Source Data file.

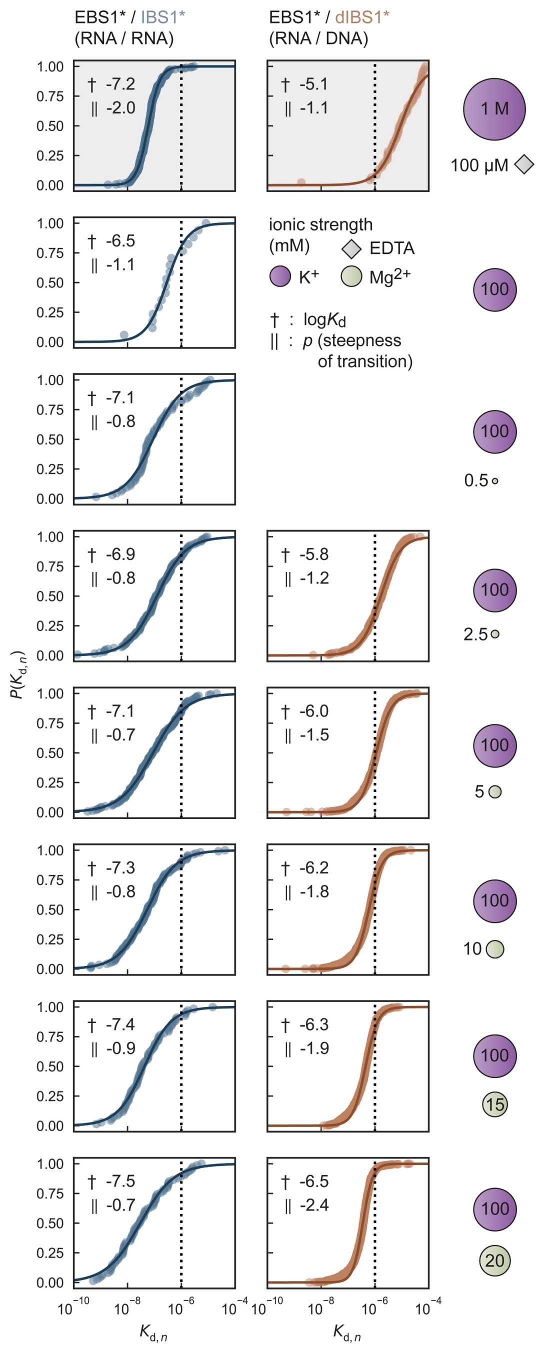

Supplementary Fig. 8 Normalized cumulative $K_{d,n}$ histograms for EBS1*/IBS1* (blue) and EBS1*/dIBS1* (orange) across different Mg^{2+} concentrations. The top row refers to a high monovalent ionic atmosphere in the absence of divalent metal ions (1 M K^+, 100 µM EDTA). A dotted vertical line is drawn at $K_d = 10^{-6}$ M as a guide for the eye. A homogeneous logistic model (Eqn. S13) is fitted to the cumulative histograms. The sigmoid is characterized by two parameters, the midpoint K_d and the steepness of the curve p, which are indicated alongside each plot.

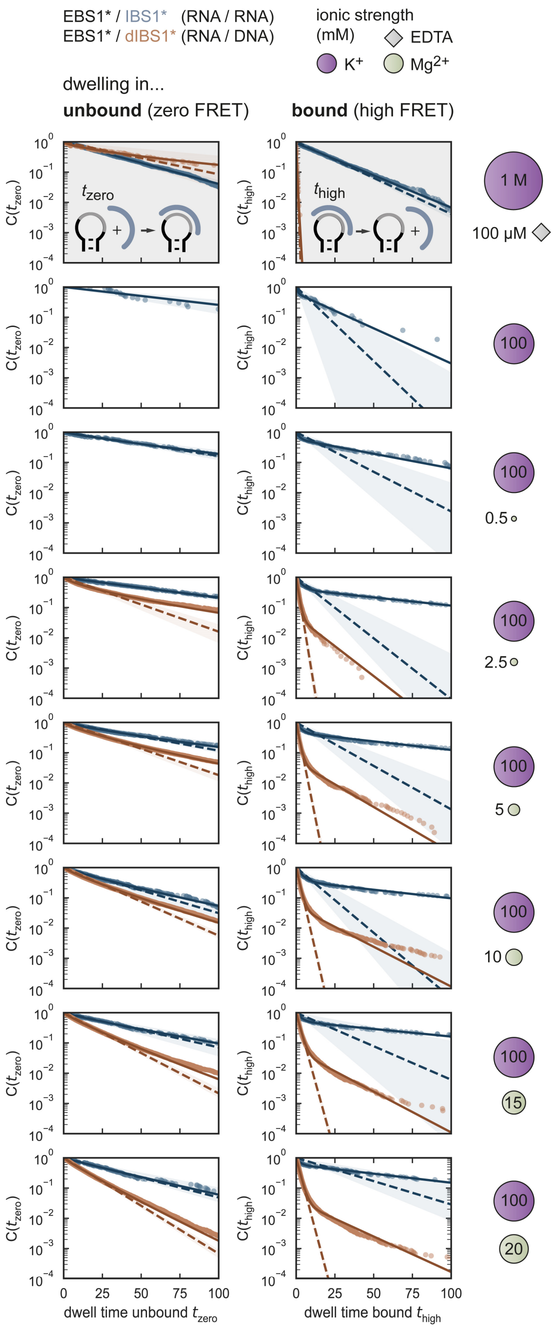

Supplementary Fig. 9 Complementary cumulative dwell time distributions of the unbound (left) and bound (right) state for EBS1*/IBS1* (blue) and EBS1/dIBS1* (orange) across different Mg^{2+} concentrations. The complementary cumulative distribution $C(t_{zero})$ and and $C(t_{high})$ represent the probability of a molecule to survive beyond a time t_{zero} or t_{high} in the given state. The characteristic dwell time decay constants τ_{zero} and τ_{high} are obtained by fitting equation S2 (for τ_{high}) or equation S3 (for τ_{zero}) to the distributions. Single-exponential fits (homogeneous binding/unbinding kinetics) are drawn as dashed lines. They don't account for the heterogeneous unbinding kinetics in either of the two systems. The 0.99 bootstrap confidence interval is indicated by shading. The logarithmic scaling emphasizes longer dwell times also if they are low-abundant. Importantly, some heterogeneity persists even in the RNA/DNA hybrids, yet to a much lower extent that in the RNA/RNA contact. The top row refers to a high monovalent ionic atmosphere in the absence of divalent metal ions (1 M K^+, 100 µM EDTA). Source data are provided as a Source Data file.

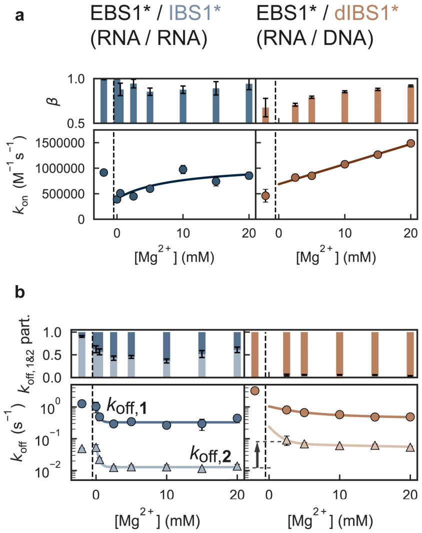

Supplementary Fig. 10 Binding and unbinding rates from complementary cumulative dwell time histograms for EBS1*/IBS1* (blue) and EBS1*/dIBS1* (orange) across different Mg^{2+} concentrations. (a) Second-order binding rates, k_{on}, are obtained from fitting a stretched single-exponential function (Eqn. S3) to the complementary cumulative unbound dwell time histograms in Supplementary Fig. **9** and taking into account the total IBS1* or dIBS1* concentration. The stretching factor β is indicated in a bar chart and represents slight inhomogeneities in the binding rates. (b) Unbinding rates $k_{off,1}$ (dark blue/orange) and $k_{off,2}$ (light blue/orange), are plotted as circles or triangles respectively. The partition coefficients (weighting factors) of the two off-rates are drawn as stacked bars. Errors are given as mean ± s.d. of 100 bootstrap samples.

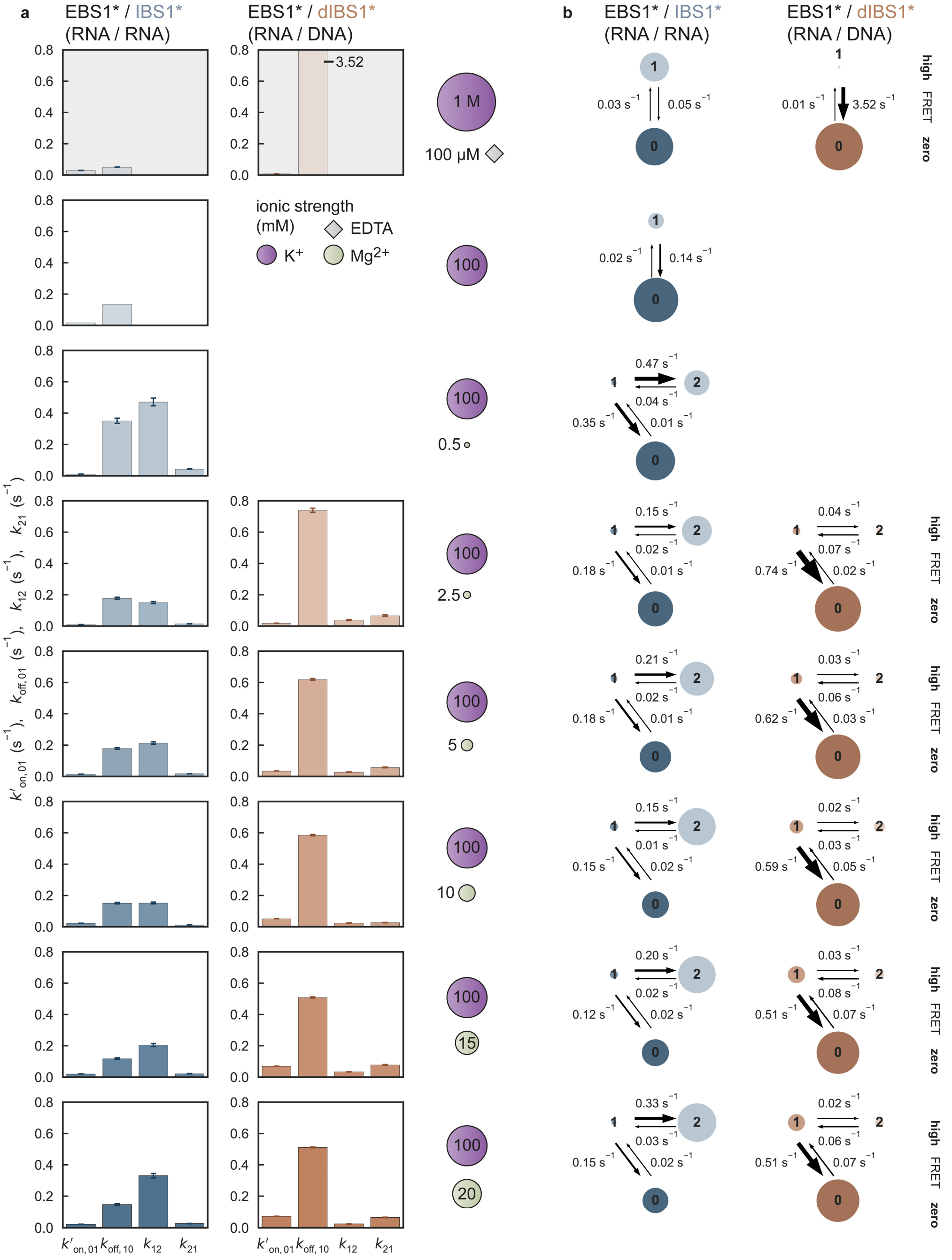

Supplementary Fig. 11 Binding and unbinding rates from a trained global hidden Markov model (HMM) for EBS1*/IBS1* (blue) and EBS1*/dIBS1* (orange) across different Mg^{2+} concentrations. (a) Bar chart of the interconversion rates $k'_{on,01}$, $k_{off,10}$, k_{12} and k_{21} obtained from training an HMM on all dynamic FRET traces. (b) Two-state (top row, 1 M K^+, 100 µM EDTA) and three-state models at different Mg^{2+} concentrations. The relative state population is proportional to the area of the circle and the magnitude of the (pseudo)first order rate coefficients is linked to the arrow width. In the three-state models the high FRET state is two-fold degenerate, that is state 1 (exon bound, Mg^{2+} unbound) and 2(exon bound, Mg^{2+} bound) have the same FRET efficiency and can only be differentiate kinetically. Errors are standard deviations calculated from likelihood ratio tests as detailed in [15]. Source data is provided in Supplementary Table 4.

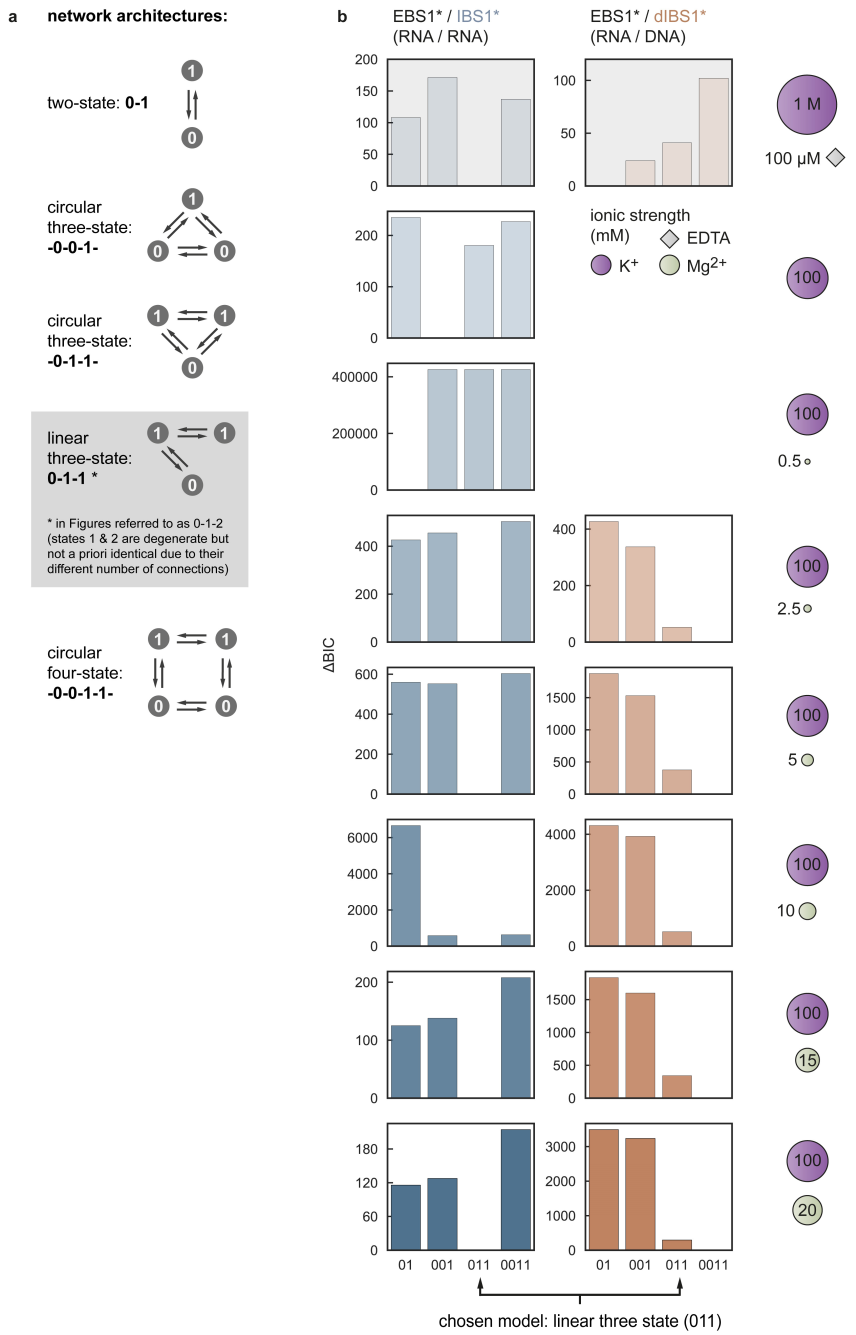

Supplementary Fig. 12 Bayesian Information criterion (BIC) of trained hidden Markov models. (a) Different network architectures of the tested HMMs. (b) Bar plots show ΔBIC values with respect to the best model where the absolute BIC value is minimized. Since the number of states is determinant for the criterion, the circular and linear three state model 011 have the same BIC value. The BIC is used in combination with biochemical knowledge about the system to select the most appropriate model (linear three-state: 0-1-1) which is shaded in gray.

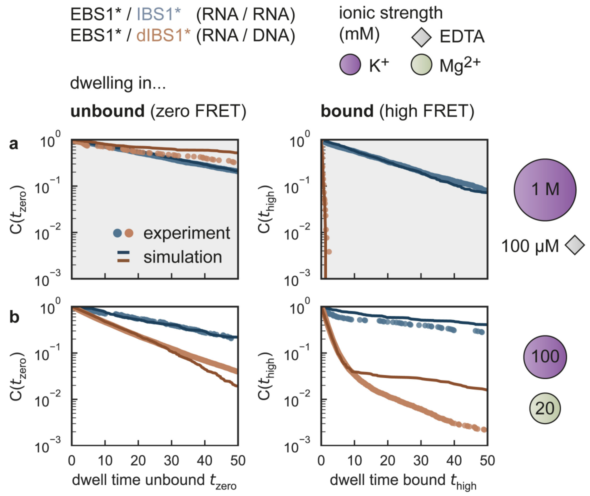

Supplementary Fig. 13 Dwell time distributions reproduced from simulation of the HMM derived rate system (linear three-state model, Supplementary Fig. **11**). Simulated (lines) and experimental (dots) decays are overlaid for (a) 1 M K^+ and 100 μM EDTA and (b) 100 mM K^+ and 20 mM Mg^{2+}. Experimental and simulated distributions are in good agreement, thus supporting the chosen kinetic model.

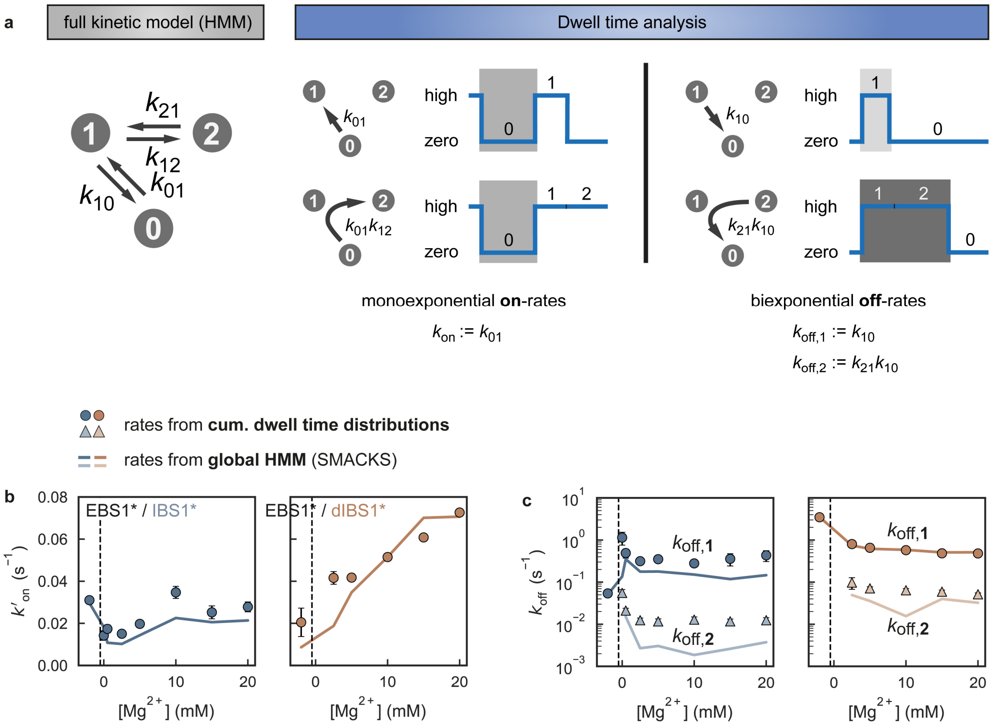

Supplementary Fig. 14 Comparison of dwell time analysis with hidden Markov modeling (HMM). (a) The HMM includes interconversion rates k_{12} and k_{21} between the degenerated kinetic states 1 and 2. Dwell time analysis of the FRET states (bound/unbound) is blind to this interconversion. Hence, the observed on-rate is mono-exponential. Dissociation from the bound state can occur from either kinetic state 1 or 2 and therefore lead to a bi-exponential decay where $k_{off,1}$ is equal to k_{10} and $k_{off,2}$ is the product of k_{21} and k_{10}. (b/c) Comparison of kinetic rates determined from fits to cumulative dwell time distributions (markers) and extracted from the trained global hidden Markov model (lines) as a function of the Mg^{2+} concentration.

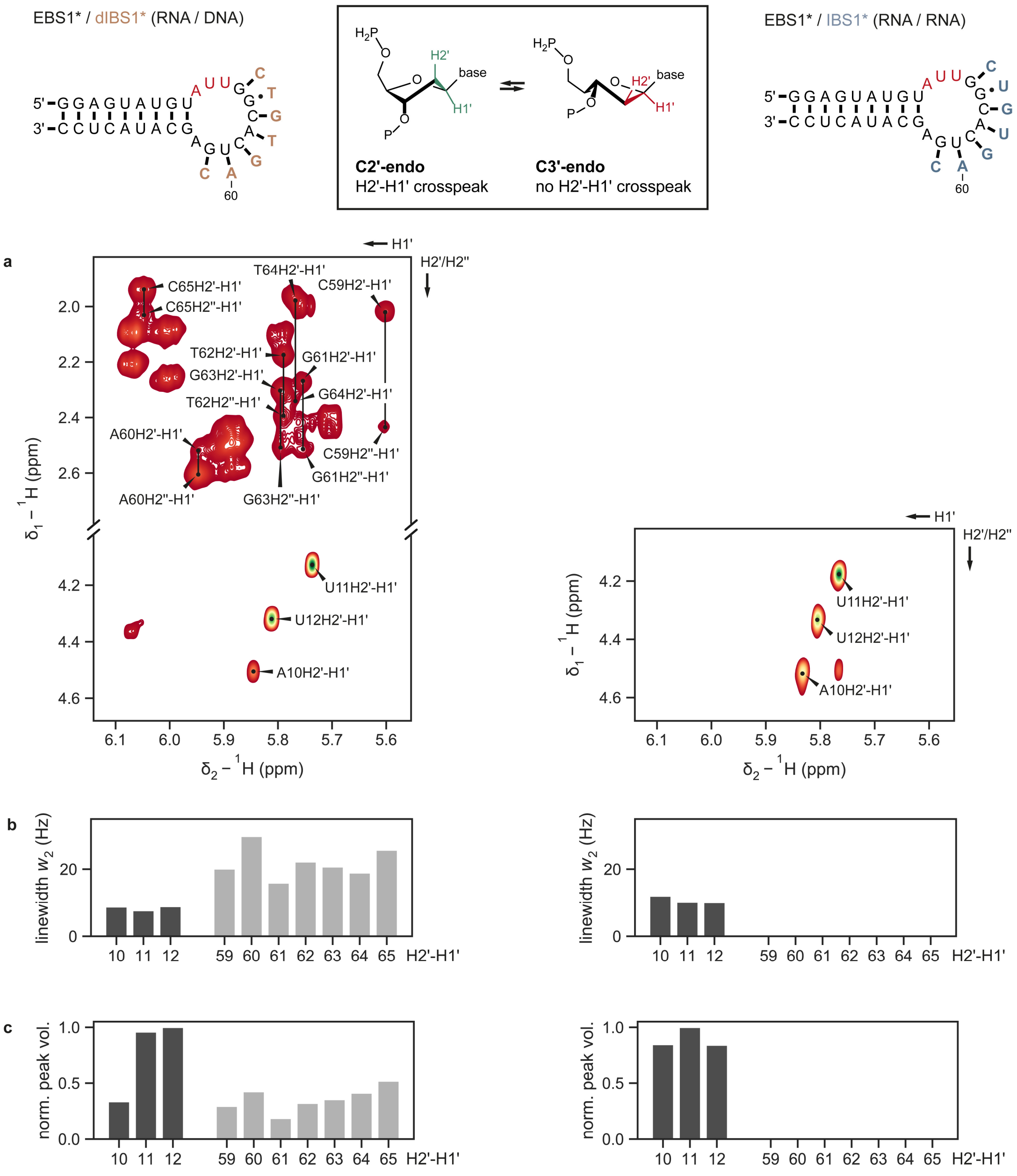

Supplementary Fig. 15 Weak H2'-H1' coupling and broad linewidths suggest exchange between sugar pucker conformations in dIBS1*. (a) [^{1}H,^{1}H]-TOCSY spectrum of EBS1*/dIBS11* and EBS1*/IBS1*. In a sugar C2'-endo conformation, coupling between H2' and H1' gives rise to a strong cross-peak as seen for nucleotides U11 and U12 (marked in red for both EBS1*/dIBS11* and EBS1*/IBS11*). In a C3'-endo sugar the H2' and H1' protons are nearly perpendicular to each other, which weakens the ^3J-coupling constant and no cross peak is observed (EBS1*/IBS1*). (b) Linewidths and (c) peaks volumes are calculated by 2D-Gaussian fits. Broad line shapes and intermediary peak volumes for H2'-H1' correlations of all residues in dIBS1*, suggesting fast interconversion between sugar puckers (spectrum of EBS1*/IBS1* adapted from ref [14]). On the contrary, IBS1* shows no H2'-H1' correlations (H2' chemical shifts in RNA usually lie in the range of 4-5 ppm), which points to a stable C3'-endo conformation. Spectra were recorded at 298 K in D_2O in the presence of 110 mM KCl. Source data are provided as a Source Data file.

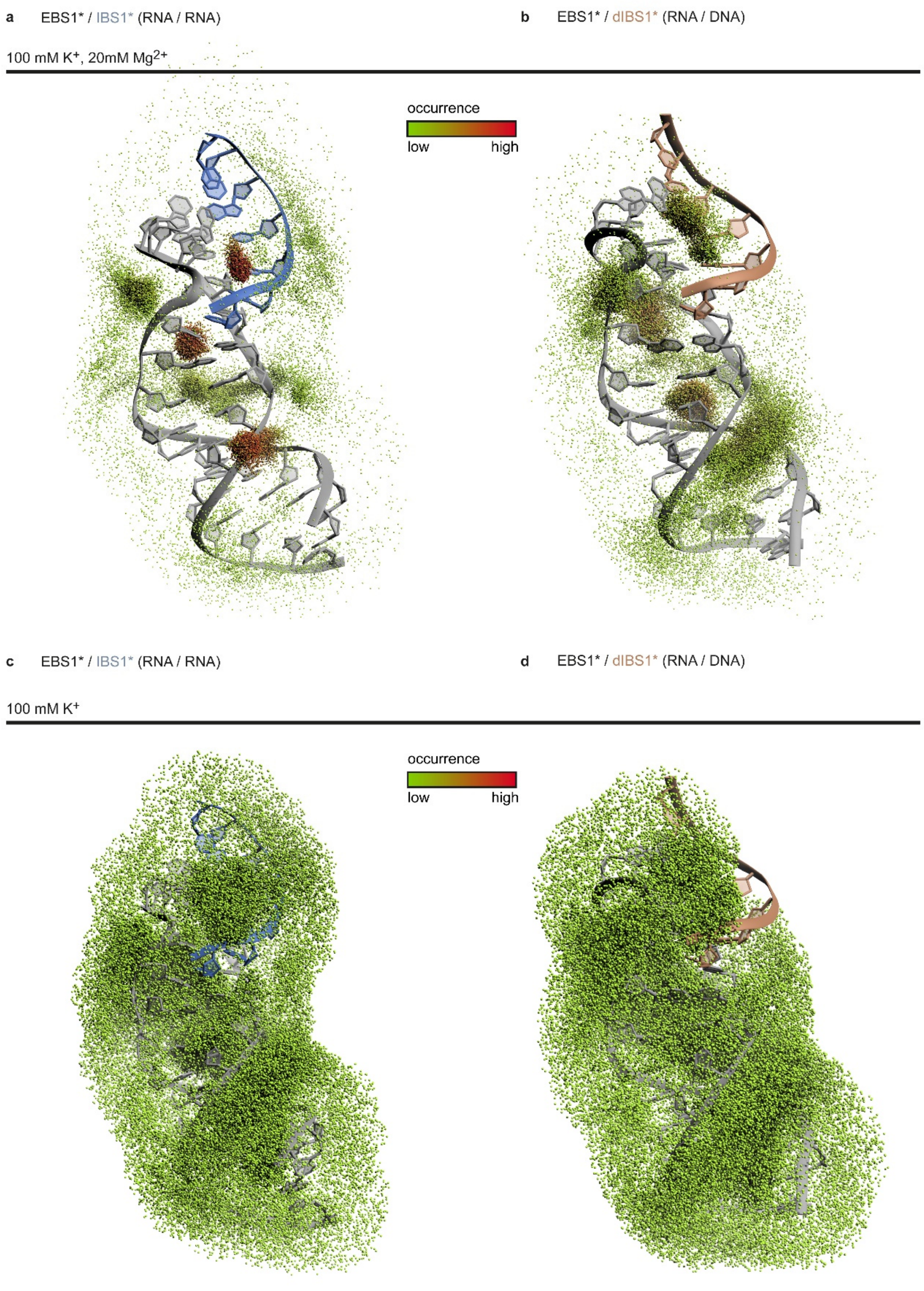

Supplementary Fig. 16 Spatial distribution of K^+ and Mg^{2+} binding from MD simulations. All ion positions (Mg^{2+} for a/b; K^+ for c/d) within 6 Å of the nucleic acids are color-coded based on their occurrence (highest: red, lowest: green). The colors are scaled to the same probability density across all four simulations. (a/b) Mg^{2+} binding based on experimentally observed coordination sites (NOEs to $[Co(NH_3)_6]^{3+}$, Mg^{2+} induced chemical shifts) used as starting positions for the MD simulations. (c/d) K^+ binds much more uniformly to EBS1*/IBS1* and EBS1*/dIBS1*. The point cloud encompasses loosely associated, outer-sphere and inner-sphere bound Mg^{2+} ions.

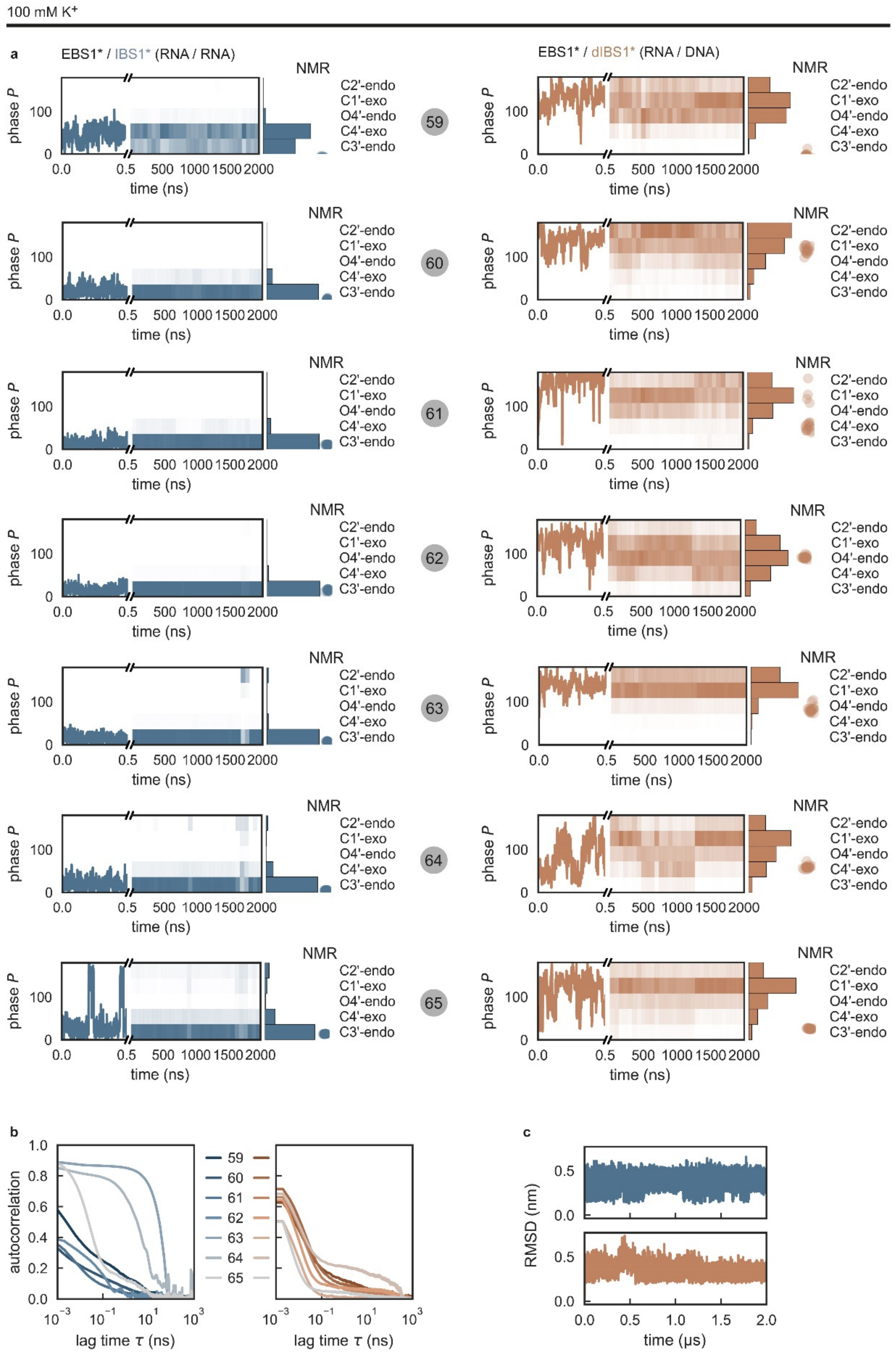

Supplementary Fig. 17 Sugar pucker dynamics of IBS1* and dIBS1* from MD simulations with 100 mM K^+ only. (a) Time evolution of the pseudorotation (phase) angle for all residues in IBS1* (blue) or dIBS1* (orange). (b) Autocorrelation of the pseudorotation angle for each residue of IBS1* or dIBS1*. (c) RMSD of the RNA dynamics along the trajectory. Source data are provided as a Source Data file.

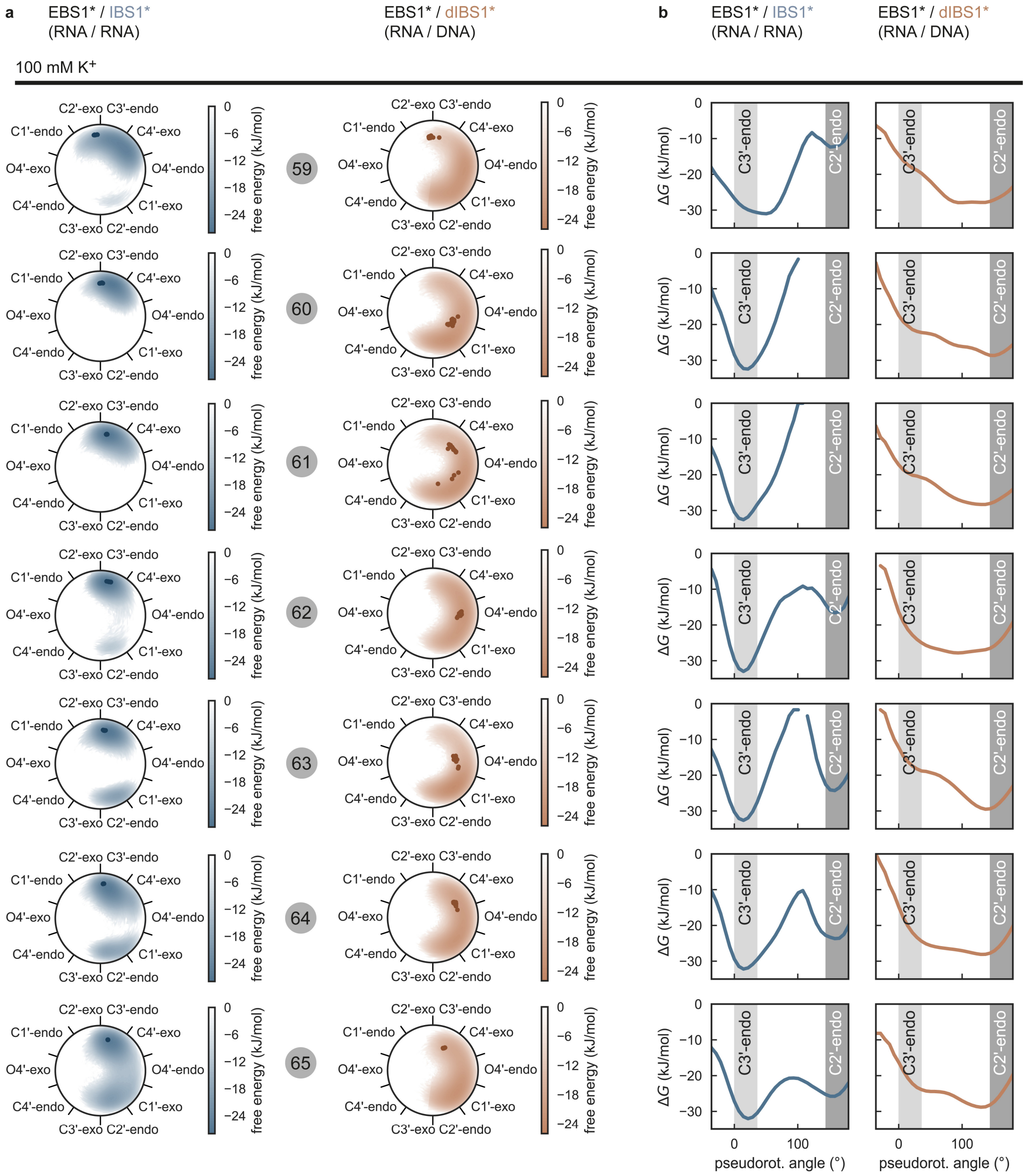

Supplementary Fig. 18 Sugar pucker conformations of IBS1* and dIBS1* from MD simulations in the absence of Mg^{2+} but with 100 mM K^+ only. (a) Pseudorotation cycles for all residues of IBS1* or dIBS1* (59-65). Distributions of pucker phase and amplitude are color coded by their free energy. The puckers of the 18 (EBS1*/IBS1*, blue) or 20 (EBS1*/dIBS1*, orange) lowest energy NMR structures are indicated by dots. (b) Free energy profiles of the pseudorotation angle (pucker phase) for all residues of IBS1* or dIBS1*. Source data are provided as a Source Data file.

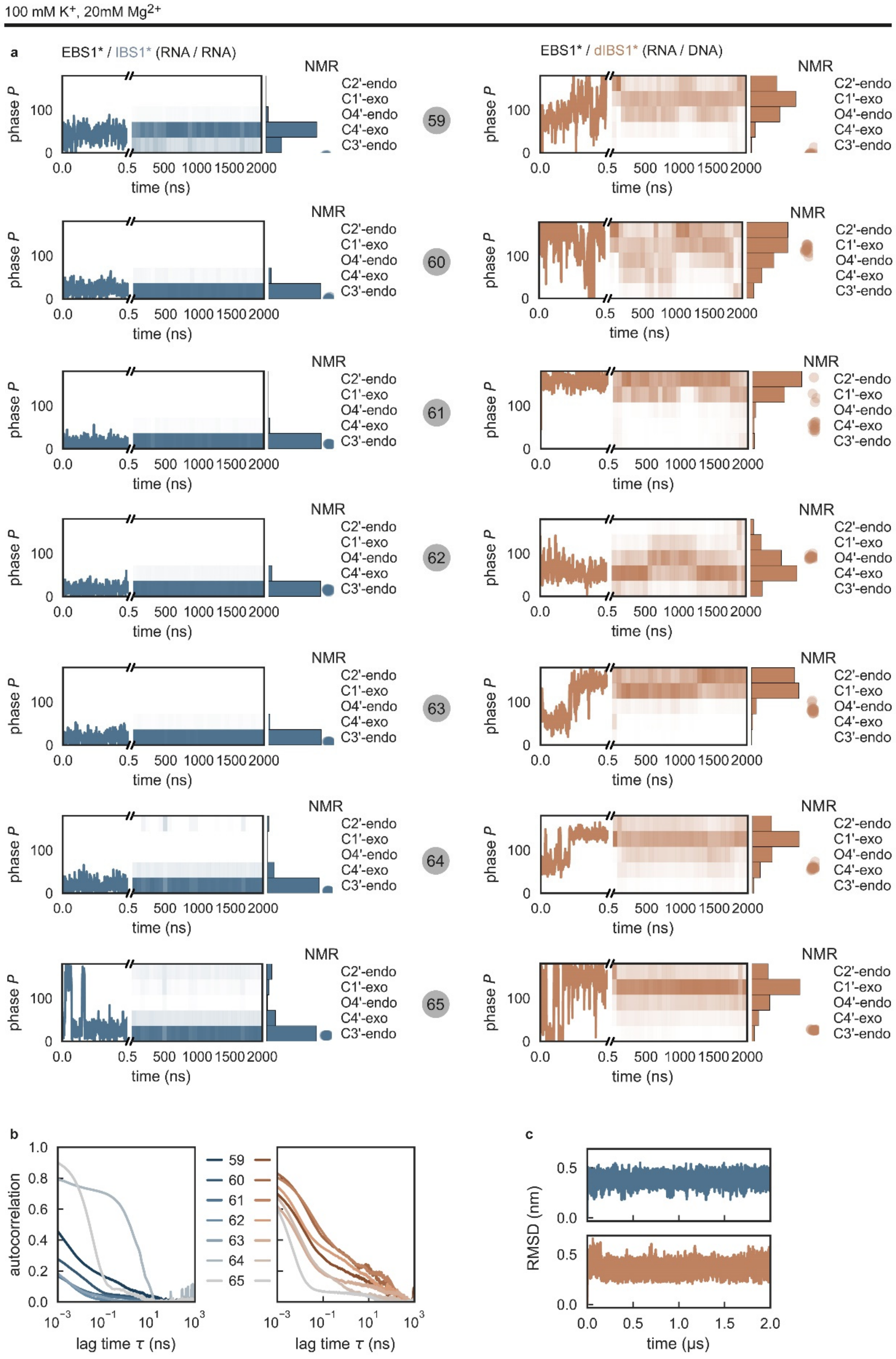

Supplementary Fig. 19 Sugar pucker dynamics of IBS1* and dIBS1* from MD simulations in the presence of 100 mM K^+ and 20 mM Mg^{2+}. (a) Time evolution of the pseudorotation (phase) angle for all residues in IBS1* (blue) or dIBS1* (orange). (b) Autocorrelation of the pseudorotation angle for each residue of IBS1* or dIBS1*. (c) RMSD of the RNA dynamics along the trajectory. Source data are provided as a Source Data file.

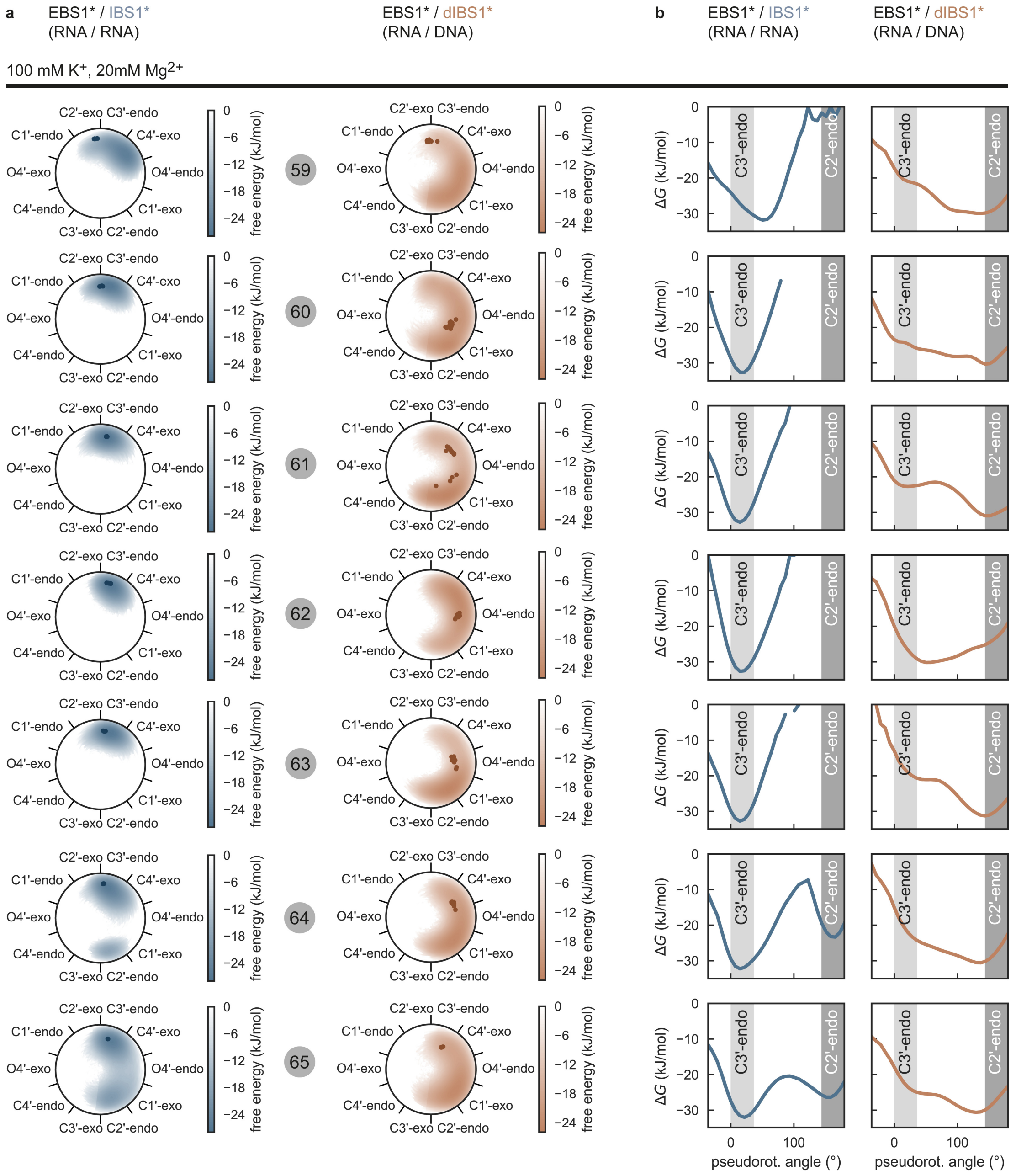

Supplementary Fig. 20 Sugar pucker conformations of IBS1* and dIBS1* from MD simulations with 100 mM K^+ and 20 mM Mg^{2+}. (a) Pseudorotation cycles for all residues of IBS1* or dIBS1* (59-65). Distributions of pucker phase and amplitude are color coded by their free energy. The puckers of the 18 (EBS1*/IBS1*, blue) or 20 (EBS1*/dIBS1*, orange) lowest energy NMR structures are indicated by dots. (b) Free energy profiles of the pseudorotation angle (pucker phase) for all residues of IBS1* or dIBS1*. Source data are provided as a Source Data file.

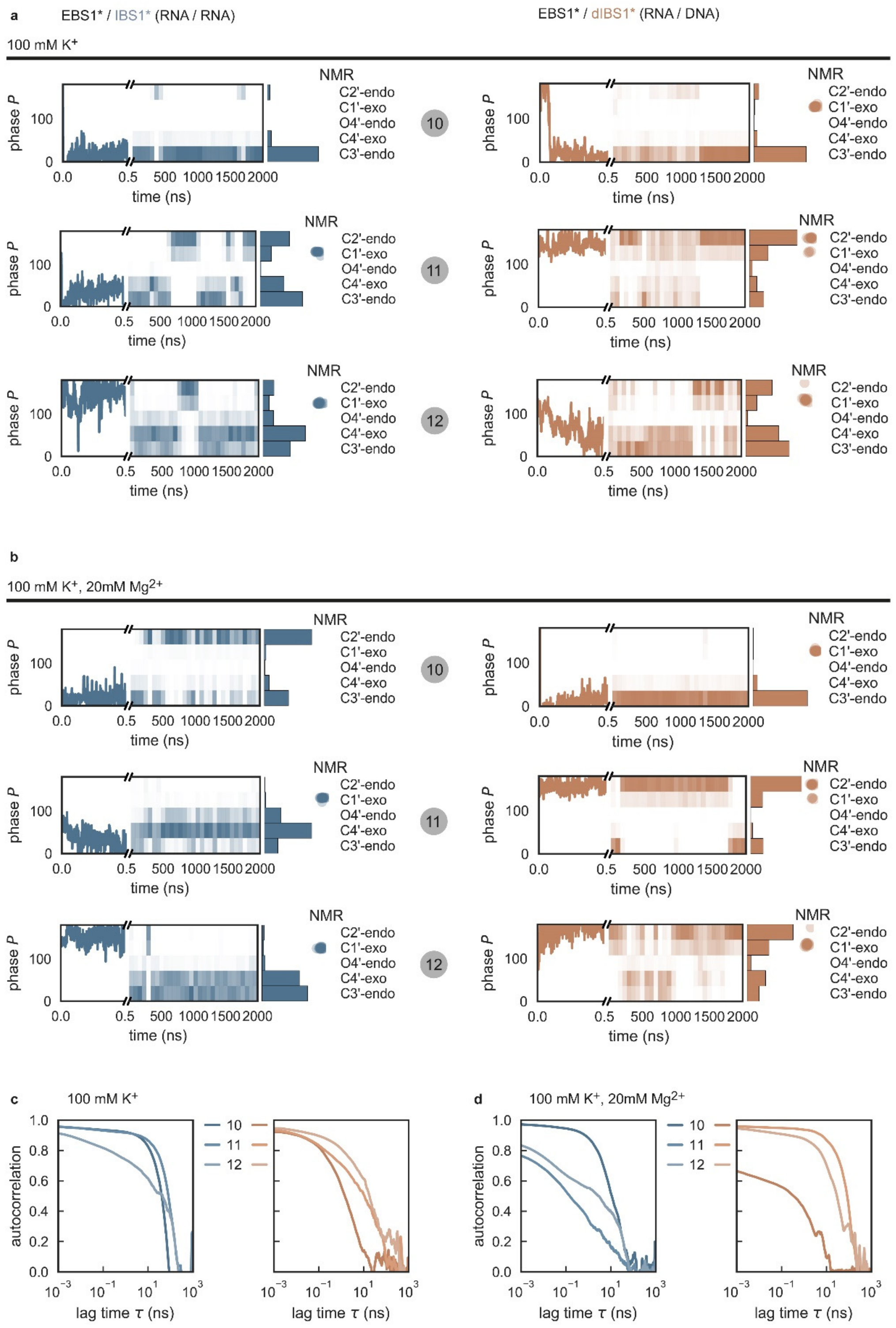

Supplementary Fig. 21 Sugar pucker dynamics of EBS1* residues A10-U12 from MD simulations in the presence and absence of Mg^{2+}. (a/b) Time evolution of the pseudorotation (phase) angle for the hairpin residues A10, U11 and U12 in the RNA-RNA (blue) or RNA-DNA contact (orange) in the presence and absence of Mg^{2+}. (c/d) Autocorrelation of the pseudorotation angle. Source data are provided as a Source Data file.

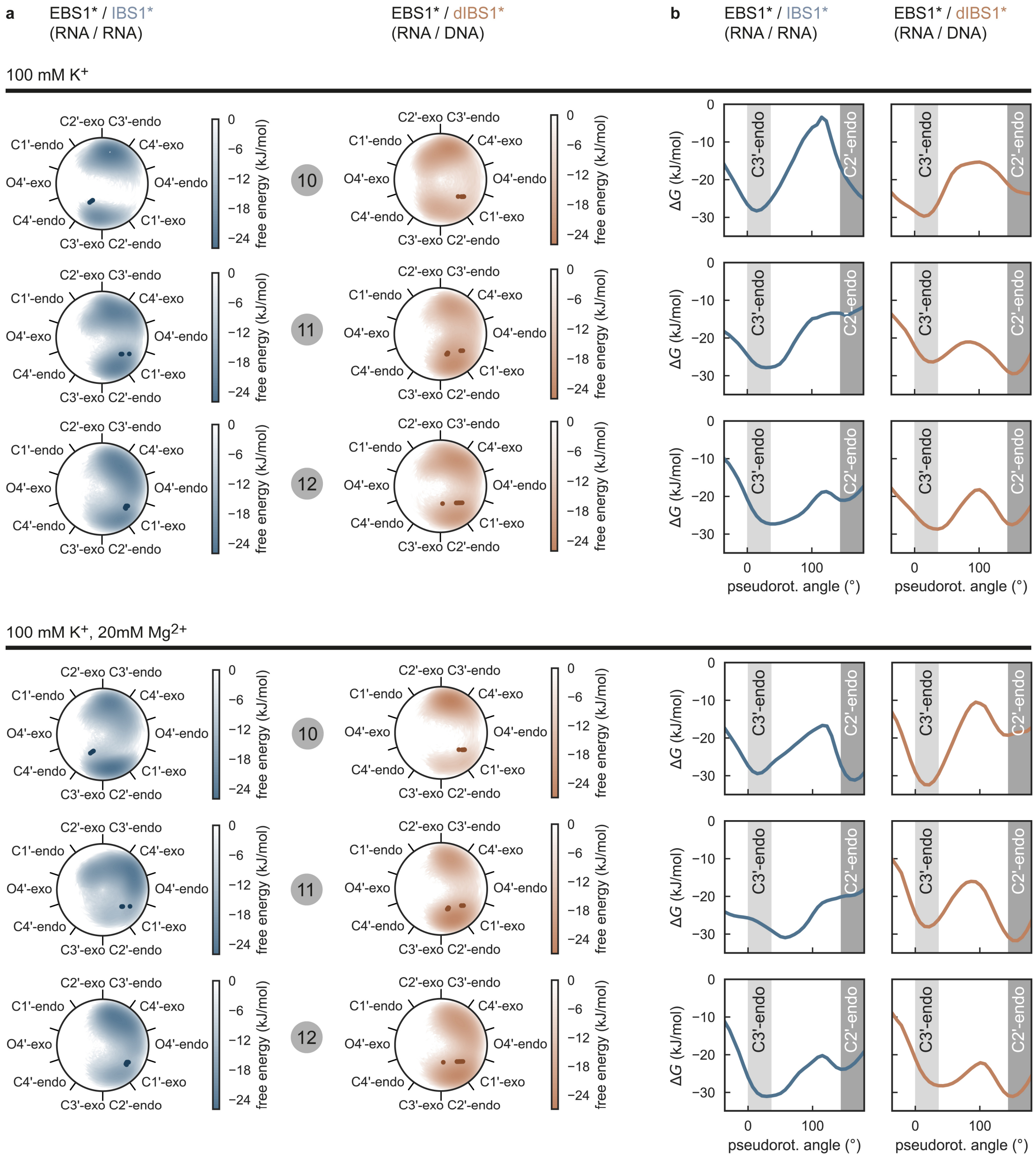

Supplementary Fig. 22 Sugar pucker conformations of EBS1* residues 10-12 from MD simulations in the presence and absence of Mg^{2+}. (a) Pseudorotation cycles for A10, U11 and U12. Distributions of pucker phase and amplitude are color coded by their free energy. The puckers of the 18 (EBS1*/IBS1*, blue) or 20 (EBS1*/dIBS1*, orange) lowest energy NMR structures are indicated by dots. (b) Free energy profiles of the pseudorotation angle (pucker phase) for all residues of IBS or dIBS. Source data are provided as a Source Data file.

Suppementary References

1. Kruschel, D. & Sigel, R. K. O. Divalent metal ions promote the formation of the 5'-splice site recognition complex in a self-splicing group II intron. *J. Inorg. Biochem.* **102,** 2147–2154 (2008).
2. Kowerko, D. *et al.* Cation-induced kinetic heterogeneity of the intron-exon recognition in single group II introns. *Proc. Natl. Acad. Sci. U. S. A.* **112,** 3403–3408 (2015).
3. Floyd, D. L., Harrison, S. C. & van Oijen, A. M. Analysis of kinetic intermediates in single-particle dwell-time distributions. *Biophys. J.* **99,** 360–366 (2010).
4. Coelho, C. A., Mexia, T. & Mexia, J. T. On the Distribution of the Product and Ratio of Independent Generalized Gamma-Ratio Random Variables. *Sankhya* **69,** 221–255 (2007).
5. Huang, M., Giese, T. J., Lee, T.-S. & York, D. M. Improvement of DNA and RNA Sugar Pucker Profiles from Semiempirical Quantum Methods. *J. Chem. Theory Comput.* **10,** 1538–1545 (2014).
6. Börner, R. *et al.* Simulations of camera-based single-molecule fluorescence experiments. *PLoS One* **13,** e0195277 (2018).
7. Misra, V. K. & Draper, D. E. The linkage between magnesium binding and RNA folding. *J. Mol. Biol.* **317,** 507–521 (2002).
8. Draper, D. E. A guide to ions and RNA structure. *RNA* **10,** 335–343 (2004).
9. Lipfert, J., Doniach, S., Das, R. & Herschlag, D. Understanding nucleic acid-ion interactions. *Annu. Rev. Biochem.* **83,** 813–841 (2014).
10. Chu, V. B., Bai, Y., Lipfert, J., Herschlag, D. & Doniach, S. A repulsive field: advances in the electrostatics of the ion atmosphere. *Curr. Opin. Chem. Biol.* **12,** 619–625 (2008).
11. Jurrus, E. *et al.* Improvements to the APBS biomolecular solvation software suite. *Protein Sci.* **27,** 112–128 (2018).
12. Kruschel, D., Skilandat, M. & Sigel, R. K. O. NMR structure of the 5' splice site in the group IIB intron Sc.ai5γ – conformational requirements for exon-intron recognition. *RNA* **20,** 295–307 (2014).
13. König, S. L. B. *et al.* BOBA FRET: bootstrap-based analysis of single-molecule FRET data. *PLoS One* **8,** e84157 (2013).
14. Skilandat, M. & Sigel, R. K. O. The role of Mg(II) in DNA cleavage site recognition in group II intron ribozymes: solution structure and metal ion binding sites of the RNA-DNA complex. *J. Biol. Chem.* **289,** 20650–20663 (2014).
15. Schmid, S. & Hugel, T. Efficient use of single molecule time traces to resolve kinetic rates, models and uncertainties. *J. Chem. Phys.* **148,** 123312 (2018).
