# Supplementary material for: Metal ions and sugar puckering balance single-molecule kinetic heterogeneity in RNA and DNA tertiary contacts
Source: Nat Commun. 2020 Jan 8;11:104. doi: 10.1038/s41467-019-13683-4 (PMC6949254; doi:10.1038/s41467-019-13683-4)
Supplement: Supplementary file 3 — Reporting Summary [file 41467_2019_13683_MOESM3_ESM.pdf]

nature research

Corresponding author(s): Roland K.O. Sigel, Richard Börner

Last updated by author(s): Nov 14, 2019

Reporting Summary

Nature Research wishes to improve the reproducibility of the work that we publish. This form provides structure for consistency and transparency in reporting. For further information on Nature Research policies, see Authors & Referees and the Editorial Policy Checklist.

Statistics

For all statistical analyses, confirm that the following items are present in the figure legend, table legend, main text, or Methods section.

n/a	Confirmed	
☐	☒	The exact sample size (*n*) for each experimental group/condition, given as a discrete number and unit of measurement
☐	☒	A statement on whether measurements were taken from distinct samples or whether the same sample was measured repeatedly
☐	☒	The statistical test(s) used AND whether they are one- or two-sided<br>*Only common tests should be described solely by name; describe more complex techniques in the Methods section.*
☐	☒	A description of all covariates tested
☐	☒	A description of any assumptions or corrections, such as tests of normality and adjustment for multiple comparisons
☐	☒	A full description of the statistical parameters including central tendency (e.g. means) or other basic estimates (e.g. regression coefficient) AND variation (e.g. standard deviation) or associated estimates of uncertainty (e.g. confidence intervals)
☒	☐	For null hypothesis testing, the test statistic (e.g. *F*, *t*, *r*) with confidence intervals, effect sizes, degrees of freedom and *P* value noted<br>*Give P values as exact values whenever suitable.*
☐	☒	For Bayesian analysis, information on the choice of priors and Markov chain Monte Carlo settings
☒	☐	For hierarchical and complex designs, identification of the appropriate level for tests and full reporting of outcomes
☒	☐	Estimates of effect sizes (e.g. Cohen's *d*, Pearson's *r*), indicating how they were calculated

Our web collection on statistics for biologists contains articles on many of the points above.

Software and code

Policy information about availability of computer code

Data collection	Andor Solis (camera video aquisition software for recording single-molecule videos), GROMACS 5.1 (Molecular Dynamics simulations)
Data analysis	Matlab: MASH-FRET v1.1 (analysis of single-molecule video data, available on github https://rna-fretools.github.io/MASH-FRET/ with documentation at https://rna-fretools.github.io/MASH-FRET/), Igor Pro: SMACKS v1.0 (ensemble-HMM for state transition detection of degenerated states or heterogenous kinetics, available online https://www.singlemolecule.uni-freiburg.de/software/smacks), Python for data plotting and visualization.

For manuscripts utilizing custom algorithms or software that are central to the research but not yet described in published literature, software must be made available to editors/reviewers. We strongly encourage code deposition in a community repository (e.g. GitHub). See the Nature Research guidelines for submitting code & software for further information.

Data

Policy information about availability of data

All manuscripts must include a data availability statement. This statement should provide the following information, where applicable:

- Accession codes, unique identifiers, or web links for publicly available datasets
- A list of figures that have associated raw data
- A description of any restrictions on data availability

Data supporting the findings of this manuscript are available from the corresponding authors upon reasonable request. The source data underlying Figures 1d-g, 2a-g, 3a/b, 4c, 5b/c and Supplementary Figures S1, S2, S7, S9, S15, S17-22 is provided as a Source Data file.

Field-specific reporting

Please select the one below that is the best fit for your research. If you are not sure, read the appropriate sections before making your selection.

☒ Life sciences ☐ Behavioural & social sciences ☐ Ecological, evolutionary & environmental sciences

For a reference copy of the document with all sections, see nature.com/documents/nr-reporting-summary-flat.pdf

Life sciences study design

All studies must disclose on these points even when the disclosure is negative.

Sample size	To ensure statistical robustness, each sample and condition includes at least 100 single-molecule trajectories. Usually, hundreds of single-molecule trajectories were inspected and 100-1500 molecules were selected.
Data exclusions	Single-molecule spots in the raw video data were detected with a spot-finding algorithm (in-series screening with a threshold criterion and 3 px minimum edge distance, 3 px minimum spot-to-spot distance). Spots not fullfilling these criteria were excluded. After trace generation individual single-molecule fluorescence trajectories were sorted with regard to state transitions (dynamic, static and zero). Trajectories showing photophysical artefacts (fluorescence blinking, multiple photobleaching steps) were excluded from further data analysis.
Replication	All experiments were carried out at least three times. Single-molecule data from individual videos of different experiments but identical conditions were summarized in one set of trajectories. Thus, sample variation/variability is included. Bootstrapping was performed to calculate error estimates on thermodynamic and kinetic parameters. Ensemble hidden Markov models were evaluated on the basis of a Bayesian information criterion (BIC) and confidence intervals were computed by systematic variation of the transitions rates and evaluated by likelihood ratio tests.
Randomization	Single-molecule data is randomized per se. All single-molecule trajectories which fullfill the above mentioned pre-selection criteria build up a randomized sample of at least 100 individuals.
Blinding	Experiments were not blinded.

Reporting for specific materials, systems and methods

We require information from authors about some types of materials, experimental systems and methods used in many studies. Here, indicate whether each material, system or method listed is relevant to your study. If you are not sure if a list item applies to your research, read the appropriate section before selecting a response.

Materials & experimental systems

n/a	Involved in the study
☒	☐ Antibodies
☒	☐ Eukaryotic cell lines
☒	☐ Palaeontology
☒	☐ Animals and other organisms
☒	☐ Human research participants
☒	☐ Clinical data

Methods

n/a	Involved in the study
☒	☐ ChIP-seq
☒	☐ Flow cytometry
☒	☐ MRI-based neuroimaging
