# Supplementary material for: Metal ions and sugar puckering balance single-molecule kinetic heterogeneity in RNA and DNA tertiary contacts
Source: Nat Commun. 2020 Jan 8;11:104. doi: 10.1038/s41467-019-13683-4 (PMC6949254; doi:10.1038/s41467-019-13683-4)
Supplement: Supplementary file 2 — Peer Review File [file 41467_2019_13683_MOESM2_ESM.pdf]

Reviewers' comments:

Reviewer #1 (Remarks to the Author):

This thorough and scholarly study provides clear evidence for differences in the dynamics of RNA/RNA and RNA/DNA helices that arise when Mg2+ ions bind the RNA duplexes. Although I wouldn't have thought there was much to learn on this topic, in fact, this study convinces me otherwise. Given that elemental RNA interactions are important for dissecting the overall behavior of larger RNA molecules, the take-home points regarding the effects of ion binding and sugar puckering will be valuable for other RNAs. The general applicability of their analysis should make this study of interest to many groups working on non-coding RNA or nucleic acid structure in general.

The strength of this study is the careful and exceptionally thorough analysis of the smFRET data and the effort to explain the findings with MD simulations.

The weakness of the study is that the specific conclusions about sugar puckering rest almost entirely on the MD simulations. Of course, it is well known that ribose and deoxyribose sugars have different preferred conformations, which is also indicated in previous NMR data. However, the authors specifically claim that puckering accounts for the different dynamics they observe. Without some experimental information about the ribose conformational dynamics, or, at least, benchmarking of the simulated sugar puckers, I think the authors have to back down on this claim (and change the title of the paper). I do realize that force fields for RNA simulations have improved, but they still have many shortcomings, especially when it comes to reproducing Mg2+ ion binding effects. I think the simulations can indicate a plausible explanation but are not sufficient to prove a point.

Another limitation of the study is that the authors only study the EBS/IBS interaction in isolation and not in the context of the full group II intron. It seems it should have been possible to also study 5 exon binding to the whole intron – did the authors consider this, and does the remainder of the intron increase or diminish the heterogeneity of the 5 exon binding lifetimes?

The figures in this paper are beautifully crafted and color coordinated, although they are also very intricate. I found the text much less clear; I suspect the authors are not writing what they mean, but even if they do mean what they write, a number of points need to be cleaned up.

Specific comments:

1. The authors introduce their study in terms of intrinsic effects of interactions between nucleic acids and metal ions, but it seems they miss an opportunity to explain the biological implications of their findings. For example, they could point out the consequences of losing a grip on the 5' exon before the second step of splicing takes place. In addition, they might point out that group II introns can also recognize DNA during retrohoming.

2. The transition to the experimental system is too brief on pg. 3. After noting that tertiary interactions within the group II intron stabilize the EBS/IBS interaction, they offer no justification for studying this interaction in isolation. As it happens, the isolated interaction is still quite interesting, but they still need to put it in context and justify this choice of system.

3. The analysis and importance of inter-molecule heterogeneity was very hard to understand and should be presented more clearly. First, it is not clear to me that heterogeneity between molecules is important for the story, whereas interstate heterogeneity clearly IS important. To what degree is the heterogeneity between molecules greater than the distribution of lifetimes observed for each molecule? It is difficult to tell from the traces and from Fig. 2 how much this is true. First, the authors reasonably show only the average lifetimes for each molecule in Fig. S4, but one would also want to know how the spread for each molecule compares to the differences between their averages. Second,

if an individual tethered IBS hairpin shows persistent stable or unstable binding over many minutes, what is the origin of this effect, and does it correlate with the distribution of short and long lifetimes of the bound state (interstate heterogeneity)? It is hard to imagine that this has to do with transient states such as sugar puckering or Mg2+ binding.

Minor:
4. Pg. 2, bottom, the sentence “A classic example of a heterogeneous system…” sounds odd to me – why is an RNA hairpin any more heterogeneous than other RNA structures, such as pseudoknots or helix junctions or K-turns? Of, by “classic”, do the authors mean that RNA hairpins have been the most studied, and therefore their heterogeneity has been documented?

5. In the Methods (supplement), please correct missing references to equations.

6. Fig. 1, this figure is laid out beautifully (notwithstanding the unrealistic depiction of the RNA double helix relative to streptavidin in 1c!). However, panel f would be more intuitive if the labels RNA/RNA and RNA/DNA were put at the top of each column of plots, rather than in an empty space.

7. In Fig. 1g, I don’t understand what the authors mean by “fraction of intact tertiary contacts”. Is this referring to stable high FRET complexes? If so, “stable binding” or “fraction bound” would be much more intuitive.

8. In Fig. 2b, removing the violet arrows showing K+ data, which are represented by the squares and easy to see, would help declutter the figure.

9. In Fig. 2b-d, it is very difficult to evaluate the spread in lifetimes from the figure – the blue and orange dots overlap too much. It may be necessary to plot the data on separate pairs of axes as in Fig. S4; the key can be moved or eliminated to create some additional space.

10. On pg. 5, bottom, the authors write that the equivalence between 1 M K+ and mM Mg2+ is “intriguing”. This is not at all intriguing; it is the expected result and has been observed by many authors. This arises from the different tendencies of monovalent and divalent ions to condense around nucleic acids.

11. On pg. 5, bottom, the authors state that Mg2+ binding is site-specific, but they haven’t presented us with any evidence yet that this is true, so this statement should be revised accordingly.

12. Fig. 4, the legend refers to crosses that represent the lowest energy configurations from NMR structures, but these are not visible in the figure.

13. Fig. 4a, I suggest coloring the metal ion spheres something other than gray so they stand out. Also, please make it more clear in the figure legend or in the main text how these metal ions were assigned in the NMR experiment – is this from modeling of the electrostatic potential of the RNA structure, or from direct spectroscopic probes for RNA-metal ion interactions?

14. Fig. S8 – please check the figure legend; shouldn’t the RNA/RNA complex show more heterogeneous kinetics than the RNA/DNA hybrid?

Reviewer #2 (Remarks to the Author):

Steffen et al investigate the thermodynamics and kinetics of exon recognition by a group II intron. They use a combination of MD simulations and single-molecule FRET experiments to characterize and directly compare RNA/RNA vs RNA/DNA hybrid contacts. The authors identify transient metal ion

binding as a major source of kinetic heterogeneity, which itself is then linked to sugar puckers.
The experiments are very well performed, with very good statistics and signal to noise. The kinetic data analysis is done extremely well and convincingly. Comparing thermodynamics and kinetics (e.g. of K+ and Mg2+ binding) the authors reveal important stabilization mechanisms. Finally, MD simulations show that puckering dynamics are the reason for the lower affinity of the intron towards DNA exons.
The interplay between puckering and metal ion coordination could be a general way to fine-tune binding affinities of RNA and DNA interactions. In my opinion, the presented findings are an important step towards a better understanding of the interplay between thermodynamic and kinetic regulation. It should therefore influence thinking in this field.
Altogether I support publication of the manuscript in Nature Communications after the following points have been addressed:

1. I found the title a little confusing / misleading, in particular as the sugar puckering is mainly based on the MD simulations and previous results, while the major part of this manuscript is single-molecule experiments and their kinetic data analysis.

2. Page 5, last sentence before the paragraph "RNA-DNA interaction...": The authors state that the stability of the tertiary contacts is dictated by the off-rates. Why is this not trivial? How would a structured transition state contribute to the stability of the tertiary contacts?

3. What are the open squares in Fig. 2b, bottom?

4. Delta_G in Fig. 5 is calculated by the Eyring model. How is this justified? Might a diffusion-based approach (e.g. Kramers) be better suited here?

5. Why are two kinetic analysis presented, the cumulative dwell time and the HMM analysis. Is the latter (in combination with the BIC) used to select the model and to double check the results from the former, or is there more to this?

6. Why is the second off rate of the HMM calculated as the product of the interconversion rate and the first off rate? Please indicate these rates (koff,2;k21; koff,1) in Fig. 3 or S10.

7. The frequent puckerings with correlation times in the mid picosecond range have only been seen in MD simulations. Is there any experimental evidence for that?

8. A logarithmic scale might be advantageous in Fig. S11.

9. Some links in the supplement are not resolved (e.g. "Error! Reference source not found" on page 3).

Reviewer #3 (Remarks to the Author):

Although the work presents some interesting data, I think that its overall significance does not justify publication in Nature Communications.

Understandably, the authors have tried to write the text in a way expected for high-profile journals. Unfortunately, this has often lead to overstatement and formulations that are difficult to understand. For example, in the introduction the text states: “While transient Mg2+ association has been identified as a primary cause of heterogeneity in RNA interactions, the feasibility of reversing the effect by consolidating degenerate states and reducing dissociation to a single off-rate has hardly been

investigated. Here, the sugar pucker is of special interest as a structural feature that sets RNA and DNA apart and due to its major influence on backbone dynamics."
It is not clear what the authors mean here. While I agree that transient Mg2+ association can affect RNA interactions' heterogeneity, numerous other factors are also certainly influential. Regarding the sugar pucker, it strongly affects shapes of canonical B-DNA and A-RNA helices, but generally the statement is not correct. The ability of the 2'-hydroxyl group of ribose to establish a rich spectrum of H-bonding interactions in RNA determines the difference between RNA and DNA in folded forms, and probably single-stranded ensembles.

I also have doubts about the FRET technique's capability to resolve effects of transient Mg2+ binding, due to its inherently low resolution. Key references in this section are auto-citations to works 12 and 13. Moreover, although the FRET measurements appear to have been done correctly, it is very difficult to separate the direct experimental findings (i.e., the primary data) from the author's interpretations and even speculations. This is the main weakness of the work. Generally, the authors seem to draw conclusions from FRET data about phenomena that are simply too small-scale for the technique to resolve. They also over-extrapolate their findings obtained for a model system when discussing the splicing mechanism. In a quite authoritative fashion they assume very simple few-state behavior of the system, drawing insufficiently justified structural models from the low-resolution FRET data, in my opinion. There could be structured substates in the hairpin part as well as the single-strand, which may be sensitive to the presence of divalent ions. The hairpin seems long enough to be structured in the unbound state, and formation of the complex could involve partially bound states, etc. I am not stating that this happens, but the possibility cannot be excluded.

I also have doubts about the second part of the work, the explicit-solvent MD simulations. This research looks quite weak, and lacks clear connection with the rest of the study, despite multiple opposite verbal claims in the paper. The total simulation time of 800 ns is marginal in contemporary literature. Moreover, the paper only addresses the sugar pucker dynamics although there could also be other important system descriptors. Further, there are methodological concerns. The paper states that the simulations were done with the "AMBER99sb" force field, without providing any reference. However, AMBER99sb is a protein force field and cannot be used for nucleic acid simulations. There is another "AMBER" force field, AMBER99, which can be used for nucleic acids. However, nowadays it is widely considered as obsolete and should no longer be used; see, for example, http://ambermd.org/doc12/Amber18.pdf p. 33, for current recommendations regarding use of force fields in the original AMBER package for biomolecular simulations. The authors used some other program for their simulations, but as they employed AMBER force fields they should clearly have chosen the right version and provided unambiguous references for reproducibility. I would definitely not regard AMBER99 as a suitable choice for quantitative modelling. In addition, sugar puckering is an anomeric effect that classical force fields are unlikely to describe with quantitative accuracy. The authors provide no justification that their MD simulations are stable and correctly model the studied molecules, although there are many possible causes of inaccuracies.

I do not understand a key sentence in the presumably MD part of the Results: "This local rigidity is enforced by coordination of Mg2+, packing together first shell RNA ligands." Is that an MD result? Or a speculative suggestion? The MD methodology part does NOT mention any Mg2+ ions, and specifically states that K+ ions were used. The whole MD part appears superficial and unsophisticated, with (as mentioned) unclear connection to the rest of the paper. The authors draw far-reaching conclusions based on minimal computer-modelling, and I do not grasp how they could be derived from the presented MD data. Essentially, those data merely indicate that the bound RNA exon strand is dominantly in the C3'-endo pucker while the bound equivalent DNA strand is mostly in the C2'-endo pucker with some spread (Figure 4c). This is entirely consistent with common expectations, and I think does not require any justifying MD simulations. The helix is probably in an overall A-form conformation, dictated by the RNA hairpin strand which explains some "excursions" of the DNA strand into noncanonical pucker regions. DNA is known to readily convert from B-form to A-form helices, and it can sample B-A intermediates, which have been detected multiple times by X-ray crystallography.

However, I do not understand how one could derive conclusions about thermodynamic stability from such observations.

The title and Abstract also make exaggerated claims. For example, the Abstract states that “Molecular dynamics simulations reveal an unexpected structural link between heterogeneity and the sugar puckers at the exon-intron binding interface. While inter-nucleotide strain, induced by Mg2+ ions, locks the exon in place, sugar puckering alleviates this structural rigidity and promotes exon release.” I do not understand how this could be inferred from the reported research. What is the nature of the link? What kind of inter-nucleotide strain? This appears to be simply a chain of impressive but vague statements.

My feeling is that the authors are trying to draw big conclusions, many of which cannot be directly derived from the presented research

Reviewer #4 (Remarks to the Author):

In this manuscript, the authors examine the kinetics of the exon/intron site binding in a self-splicing intron using single-molecule FRET and molecular dynamics. Dwell-times and transition rates for RNA/RNA as well as RNA/DNA hybrids (as would be the target in retrohoming) are examined as a function of K+ and Mg++ salts . Using Markov models trained on the FRET traces, it is revealed that unbinding rate is the major difference when it comes to the Mg++ dependent off-rates of the RNA/DNA hybrid. Furthermore, long molecular dynamics simulations are used to show that this could arise from fluctuations in sugar puckering between the RNA/RNA vs RNA/DNA hybrids, driven by strongly bound Mg++ ions at the binding interface.

Overall, this is a very strong and compelling work. The single-molecule analysis is particularly thorough and clearly demonstrates how varying the monovalent vs divalent salt concentration affects the thermodynamics and kinetics of the long-range interaction differently for RNA/DNA vs RNA/RNA complexes. This in of itself is a fascinating result.

The one major point that needs to be addressed is that it is unclear to what extent (if any) Mg++ is included in the molecular dynamics simulations. The authors go to great lengths to motivate that the observed sugar-puckering interconversion is putatively enforced by inner-sphere coordination of Mg++ at an interfacial location identified via CobaltHexamine binding in NMR. However, I can find no mention anywhere of how this was handled in the simulation (i.e. how many Mg++'s were simulated, where they were placed, what parameters were used, etc). If there are Mg++ present, a spatial location and dwell time analysis would seem appropriate to see if it matches the pucker interconversion frequency. If there are no Mg++ in the simulation then this must be throughly justified, as it would make it harder to support the authors conclusions that pucker interconversions are faciliated by bound Mg++. This point must be satisfactorily addressed for the story to be complete.

There are also some minor typographical issues. In Figure 3A, state 1 is assigned 63% population and state 0 37%, yet the state 0 is given the larger circle area. In the SI, pages 2 and 3 there is a bold "Error! Reference source not found"

University of Zurich[UZH]

Department of Chemistry

University of Zurich
Winterthurerstrasse 190
CH-8057 Zurich
Phone +41 44 635 46 54/80
Fax +41 44 635 68 02
http://www.chem.uzh.ch/rna

Editorial & Production Office
Nature Communications
The Macmillan Campus
4 Crinan Street
London N1 9XW, UK

Prof Dr. Roland K.O. Sigel
Biological Inorganic Chemistry
roland.sigel@chem.uzh.ch

Dr. Richard Börner
Scientific coworker and groupleader smFRET
richard.boerner@chem.uzh.ch

Zurich, October 08, 2019

Response to reviewers for Manuscript ID NCOMMS-19-16101A:

Metal ions and sugar puckering balance single-molecule kinetic heterogeneity in RNA and DNA tertiary contacts

by F. D. Steffen*, Mokrane Khier*, Danny Kowerko, R. A. Cunha, R. Börner[#] & R. K. O. Sigel[#]

Reviewer #1 (Remarks to the Author):

This thorough and scholarly study provides clear evidence for differences in the dynamics of RNA/RNA and RNA/DNA helices that arise when Mg^{2+} ions bind the RNA duplexes. Although I wouldn't have thought there was much to learn on this topic, in fact, this study convinces me otherwise. Given that elemental RNA interactions are important for dissecting the overall behavior of larger RNA molecules, the take-home points regarding the effects of ion binding and sugar puckering will be valuable for other RNAs. The general applicability of their analysis should make this study of interest to many groups working on non-coding RNA or nucleic acid structure in general.

The strength of this study is the careful and exceptionally thorough analysis of the smFRET data and the effort to explain the findings with MD simulations.

The weakness of the study is that the specific conclusions about sugar puckering rest almost entirely on the MD simulations. Of course, it is well known that ribose and deoxyribose sugars have different preferred conformations, which is also indicated in previous NMR data. However, the authors specifically claim that puckering accounts for the different dynamics they observe. Without some experimental information about the ribose conformational dynamics, or, at least, benchmarking of the simulated sugar puckers, I think the authors have to back down on this claim (and change the title of the paper). I do realize that force fields for RNA simulations have improved, but they still have many shortcomings, especially when it comes to reproducing Mg^{2+} ion binding effects. I think the simulations can indicate a plausible explanation but are not sufficient to prove a point.

Response: We thank the reviewer for this critical feedback. We very much appreciate the positive assessment of our single-molecule FRET analysis and we acknowledge the concerns raised on the MD simulations and the limits of current force fields in accurately reproducing RNA conformational dynamics, including sugar puckering, and Mg^{2+} binding. We agree with

the reviewer that there are two main problems related to biomolecular simulations: force fields accuracy and sampling. In our revised manuscript we have repeated our simulations of EBS1*/IBS1* and EBS1*/dIBS1* with the latest corrections for backbone dihedrals (ff99 + bsc0 + χOL3) implemented in the Amber-ff14 force field. We have run simulations with K^+ only with updated ion parameters and another set with Mg^{2+} placed at positions suggested by NMR chemical shifts, NOEs and line broadenings. The new simulations are fully in line with the original ones. They show better statistics as before because we have extended the simulation time to 2 μs for each system and ionic environment (Fig. S16-20). This timescale is much longer than the sugar pucker correlations times which provides good evidence that the simulations are converged with respect to sugar puckering. Furthermore, we have performed a linewidth and peak volume analysis of experimental NMR TOCSY spectra (as suggested) which provide experimental indications that the sugar puckers are in dynamic equilibrium between C2'-endo and C3'-endo conformations as suggested by the MD simulations (Fig. S15).
We are fully aware that an exact thermodynamic description of all Mg^{2+} binding positions on a tertiary contact like EBS1/IBS1 requires enhanced sampling techniques, such as temperature replica exchange or metadynamics. Implementation of these methods to accurately predict Mg^{2+} binding to RNA tertiary contacts is subject of current research in the lab and goes beyond the scope of this manuscript. We have slightly adapted the title of the paper to highlight the interplay of metal ion binding and sugar puckering in shaping kinetic heterogeneity of RNA and DNA tertiary contacts.

Another limitation of the study is that the authors only study the EBS/IBS interaction in isolation and not in the context of the full group II intron. It seems it should have been possible to also study 5 exon binding to the whole intron – did the authors consider this, and does the remainder of the intron increase or diminish the heterogeneity of the 5 exon binding lifetimes?

Response: We understand the concern of the reviewer about the limitations of studying the EBS1/IBS1 interaction in isolation. The major advantage of the engineered EBS1*/(d)IBS1* system is the straightforward fluorescence labeling and the reduction to a well-defined observable which is IBS1* binding. FRET is a suitable reaction coordinate to describe tertiary contact formation in time (dwell times) and space (transfer efficiency) and is underlined by structural data of the bound and unbound states. In this way, we could outline a detailed kinetic model of degenerated FRET states which serves as a basis for a more complex system, like the group II intron as a whole. In the context of the whole group II intron it would have been impossible to distinguish the specific effect of the here considered metal ion binding from effects of the scaffolding full group II intron, making it impossible to address the key issue discussed in this paper in such detail (see also our answer to specific comment 2 below).
To probe the same reaction coordinate in the entire intron, a fluorophore will need to be introduced site-specifically on the 5'-end of EBS1. EBS1 of *S.c.ai5γ* is located around nucleotide position 330 counting from the 5'-end of the intron with another 500-600 nucleotides coming after. Enzymatic ligation of chemically synthesized fragment, which is still the gold standard in the field, has not been routinely used for RNA of more than 200-300

nucleotides as it requires tedious optimization of the ligation sites. Longer RNAs have often only been labeled close to or at their termini. Recent advances in fluorescence labeling (e.g. Zhao et al. NAR 2018) now allow to overcome this size threshold and we are actively developing strategies to be able to measure such challenging FRET coordinates in the future. Finally, we hypothesize that kinetic and structural heterogeneity will increase in the presence of the full intron and particularly when additional maturase proteins come into play. Cryo-EM structures (Qu, Nat. Struct. Mol Biol. 2016, Haack, Cell, 2019) indicate that these proteins stabilize the exon-intron interaction and they would therefore most likely increase the number of long-lived states which we show to be the main contributor to kinetic heterogeneity.

The figures in this paper are beautifully crafted and color coordinated, although they are also very intricate. I found the text much less clear; I suspect the authors are not writing what they mean, but even if they do mean what they write, a number of points need to be cleaned up.

Response: We have revised large parts of our manuscript to convey the key messages in a more concise and more easily understandable way. We have modified parts of the Figures in the main text and included a few additional Figures in the supplementary information to better illustrate certain concepts related to heterogeneity and to corroborate the MD simulations.

Specific comments:

1. The authors introduce their study in terms of intrinsic effects of interactions between nucleic acids and metal ions, but it seems they miss an opportunity to explain the biological implications of their findings. For example, they could point out the consequences of losing a grip on the 5' exon before the second step of splicing takes place. In addition, they might point out that group II introns can also recognize DNA during retrohoming.

Response: We thank the reviewer for pointing this out. We have adapted the introduction and included a scenario of what might happen if the interaction between the exon and intron is either too weak or too strong, particularly in the case of an intron invasion event.

2. The transition to the experimental system is too brief on pg. 3. After noting that tertiary interactions within the group II intron stabilize the EBS/IBS interaction, they offer no justification for studying this interaction in isolation. As it happens, the isolated interaction is still quite interesting, but they still need to put it in context and justify this choice of system.

Response: We have reorganized the introduction to better justify why we chose to work with the isolated EBS1*/(d)IBS1* interaction. An outlook to study exon recognition in the context of the entire RNA/RNP is included in the discussion.

3. The analysis and importance of inter-molecule heterogeneity was very hard to understand and should be presented more clearly. First, it is not clear to me that heterogeneity between

molecules is important for the story, whereas interstate heterogeneity clearly IS important. To what degree is the heterogeneity between molecules greater than the distribution of lifetimes observed for each molecule? It is difficult to tell from the traces and from Fig. 2 how much this is true. First, the authors reasonably show only the average lifetimes for each molecule in Fig. S4, but one would also want to know how the spread for each molecule compares to the differences between their averages. Second, if an individual tethered IBS hairpin shows persistent stable or unstable binding over many minutes, what is the origin of this effect, and does it correlate with the distribution of short and long lifetimes of the bound state (interstate heterogeneity)? It is hard to imagine that this has to do with transient states such as sugar puckering or Mg2+ binding.

Response: We thank the reviewer for this comment. Inter-molecule heterogeneity appears if there are kinetic subpopulations of molecules, i.e. subspecies with different kinetic rates that do not interconvert. In a mean dwell time scatter plot, such subspecies would segregate into individual clusters. On the other hand, if the subpopulations interconvert rapidly within the observation time, their dwell times will average, resulting in a single but stretched cluster. We have included an additional Figure in the SI (Fig. S5) to illustrate both cases using simulations. In the single molecule experiments we see both short and long dwell times coexisting within the same trace (molecule). As suggested by the reviewer, we extended Fig. S7 to include not only the mean dwell times $\langle t_{zero} \rangle$ and $\langle t_{high} \rangle$ but also the difference between the shortest and longest dwell time, Δt_{zero} and Δt_{high}, as a measure for the spread of each trace. By correlating the mean dwell time against the time spread, we conclude that heterogeneity is induced mainly by the presence of long dwell times (Fig. S6). These long-lived states are more frequently observed the more Mg^{2+} is available. The same applies to the limiting case where an exon-bound hairpin turns into a static high FRET molecule which are higher populated the more Mg^{2+} is available. Fig. S2b shows an increasing fraction of static bound molecules over the Mg^{2+} concentration which points to a stable, and in agreement with NMR, probably inner-sphere coordination of Mg^{2+} in the tunnel shaped binding pocket at the exon-intron interface. Dynamic repuckering in EBS1*/dIBS1* likely weakens metal binding as suggested by the shift in the midpoint of the binding curve in Fig. 1g.
Overall, the heterogeneity in EBS1*/IBS1* originates mostly from the different bound state dwell times (inter-state heterogeneity). Because there is exchange between the kinetic states, we see no clear separation of timescales between the average dwell times of different molecules, thus the inter-molecule heterogeneity is comparatively low. We have revised this section of the manuscript and parts of Fig. 2 to introduce the concepts of heterogeneity more clearly.

Minor:

4. Pg. 2, bottom, the sentence "A classic example of a heterogeneous system…" sounds odd to me – why is an RNA hairpin any more heterogeneous than other RNA structures, such as pseudoknots or helix junctions or K-turns? Or, by "classic", do the authors mean that RNA hairpins have been the most studied, and therefore their heterogeneity has been documented?

Response: We used the term "classic" because an interaction between a hairpin and a complementary stretch of nucleotides is one of the simplest yet most abundant tertiary structure motif in RNA. As H-type pseudoknots this type of interaction occurs in many riboswitches and ribozymes where it brings individual secondary structure elements into a tertiary structure context. If such a motif is additionally stabilized by divalent ions, like Mg^{2+}, it is likely to show multi-exponential kinetics which is commonly referred to as kinetic heterogeneity. We understand the reviewer's concern and have therefore adapted the corresponding paragraph in the introduction.

5. In the Methods (supplement), please correct missing references to equations.

Response: The missing references have been corrected.

6. Fig. 1, this figure is laid out beautifully (notwithstanding the unrealistic depiction of the RNA double helix relative to streptavidin in 1c!). However, panel f would be more intuitive if the labels RNA/RNA and RNA/DNA were put at the top of each column of plots, rather than in an empty space.

Response: We thank the reviewer for this comment. We have adjusted the position of the labels and added a note in the Figure caption that the immobilization scheme is not drawn to scale.

7. In Fig. 1g, I don't understand what the authors mean by "fraction of intact tertiary contacts". Is this referring to stable high FRET complexes? If so, "stable binding" or "fraction bound" would be much more intuitive.

Response: The original phrase "fraction of intact tertiary contacts" refers to the fraction of intron hairpins which are base paired to the exon, or in other words the relative abundance of high FRET states among all dynamic molecules. As suggested by the reviewer we changed the axis label to "fraction of bound hairpins" and adapted the caption accordingly.

8. In Fig. 2b, removing the violet arrows showing K^+ data, which are represented by the squares and easy to see, would help declutter the figure.

Response: We have removed the arrows as suggested by the reviewer.

9. In Fig. 2b-d, it is very difficult to evaluate the spread in lifetimes from the figure – the blue and orange dots overlap too much. It may be necessary to plot the data on separate pairs of axes as in Fig. S4; the key can be moved or eliminated to create some additional space.

Response: As suggested by the reviewer, we have separated the subfigure into two individual plots and adjusted the position of the legend.

10. On pg. 5, bottom, the authors write that the equivalence between 1 M K^+ and mM Mg^{2+} is "intriguing". This is not at all intriguing; it is the expected result and has been observed by

many authors. This arises from the different tendencies of monovalent and divalent ions to condense around nucleic acids.

Response: We agree with the reviewer that this equivalence is indeed expected from the different charge densities of K^+ and Mg^{2+}. Mg^{2+} binding is entropically favored over K^+ because fewer ions are constrained to the RNA. We have adapted the paragraph and instead put the emphasis on the kinetic stabilization of the bound state by Mg^{2+}, i.e. prolongation of the dwell times in the high FRET state, which does not occur with K^+ only.

11. On pg. 5, bottom, the authors state that Mg^{2+} binding is site-specific, but they haven't presented us with any evidence yet that this is true, so this statement should be revised accordingly.

Response: Mg^{2+} binding to the EBS1*/(d)IBS1* hairpin has both a diffuse and site-specific component. While the thermodynamic contribution of diffuse ion binding is larger, there is evidence for a site-specific coordination of Mg^{2+} at the exon intron interface coming from NMR chemical shifts with Mn^{2+} and $[Co(NH_3)_6]^{3+}$. Mg^{2+} induced line broadening as well as the appearance of negatively charged binding pocket upon docking of (d)IBS1* is indicative of a strong electrostatic interaction. The effect of Mg^{2+} binding is most prominently seen on the kinetic level. Unlike molar amounts of K^+, Mg^{2+} increases the exon residence time and is strictly required for the formation of stable binding events (static bound molecules) in the concentration ranges of single-molecule imaging. We have adjusted the text accordingly.

12. Fig. 4, the legend refers to crosses that represent the lowest energy configurations from NMR structures, but these are not visible in the figure.

Response: The sugar puckers of the NMR ensemble all cluster together and therefore the crosses were not easy to distinguish. We have replaced the crosses in the insets with dots to avoid any confusion.

13. Fig. 4a, I suggest coloring the metal ion spheres something other than gray so they stand out. Also, please make it more clear in the figure legend or in the main text how these metal ions were assigned in the NMR experiment – is this from modeling of the electrostatic potential of the RNA structure, or from direct spectroscopic probes for RNA-metal ion interactions?

Response: We thank the reviewer for this comment. The outer-sphere Mg^{2+} ions in Fig. 4a are assigned based on NOE distance restraints from the RNA and DNA to the protons of the NH_3 ligands of cobalt hexamine. Because all amine protons resonate at the same frequency, a loose restraint of 3-7 A was applied from the Co^{3+} center to each proton that showed a crosspeak to the ligand (i.a. H3 and CH_3 of T62/64 and H5/6 of C65). The proposed inner-sphere binding mode is modeled based on the electrostatic surface potential. As suggested by the reviewer, we have increased the saturation of the outer-sphere Mg^{2+} to make them more visible.

14. Fig. S8 – please check the figure legend; shouldn't the RNA/RNA complex show more heterogeneous kinetics than the RNA/DNA hybrid?

Response: The RNA/RNA interaction is more heterogeneous than the RNA/DNA, the reviewer is right on that. As the logarithmic scale emphasizes low-abundant dwell times, it appears as if the RNA/DNA was more heterogeneous. We kept the mono-exponential fits in the figure (now Fig. S9) to illustrate that they don't capture the full kinetics. We used the logarithmic scale in the SI in contrast to Fig. 2f because we wanted to highlight that heterogeneity persists, although to a low percentage, even in the RNA/DNA interaction. We added a note in the Figure caption to avoid any misinterpretations.

Reviewer #2 (Remarks to the Author):

Steffen et al investigate the thermodynamics and kinetics of exon recognition by a group II intron. They use a combination of MD simulations and single-molecule FRET experiments to characterize and directly compare RNA/RNA vs RNA/DNA hybrid contacts. The authors identify transient metal ion binding as a major source of kinetic heterogeneity, which itself is then linked to sugar puckers.

The experiments are very well performed, with very good statistics and signal to noise. The kinetic data analysis is done extremely well and convincingly. Comparing thermodynamics and kinetics (e.g. of K^+ and Mg^{2+} binding) the authors reveal important stabilization mechanisms. Finally, MD simulations show that puckering dynamics are the reason for the lower affinity of the intron towards DNA exons.

The interplay between puckering and metal ion coordination could be a general way to fine-tune binding affinities of RNA and DNA interactions. In my opinion, the presented findings are an important step towards a better understanding of the interplay between thermodynamic and kinetic regulation. It should therefore influence thinking in this field.

Altogether I support publication of the manuscript in Nature Communications after the following points have been addressed:

Response: We thank the reviewer for his appreciation of the single-molecule experiments and their kinetic analysis. In the following we will address all concerns raised by the reviewer.

Minor:

1. I found the title a little confusing / misleading, in particular as the sugar puckering is mainly based on the MD simulations and previous results, while the major part of this manuscript is single-molecule experiments and their kinetic data analysis.

Response: We thank the reviewer for this critical remark. We have adapted the title to put more emphasis on the single-molecule kinetics.

2. Page 5, last sentence before the paragraph "RNA-DNA interaction...": The authors state that the stability of the tertiary contacts is dictated by the off-rates. Why is this not trivial? How would a structured transition state contribute to the stability of the tertiary contacts?

Response: We thank the reviewer for this comment. Early transition states resemble the reactant and are still mostly unstructured in the transition state (i.e. intron and exon are in proximity but base pairs are not yet fully formed). In this case, the stability of the interaction correlates with k_{off}. These types of transition states have been increasingly observed in different RNAs and are becoming a hallmark of RNA folding. In contrast, the stability of intrinsically disordered proteins is often a function of k_{on}, resulting in a transition state that is

more similar to the product and occurs late on the reaction coordinate. In this case, the energy of the transition state is sensitive to mutations that perturb the product-like structure. We have rewritten parts of the section "Off-rates determine tertiary contact stability" to describe the notion of early transition states more concisely.

3. What are the open squares in Fig. 2b, bottom?

Response: The open squares were to highlight the condition (10 mM Mg^{2+}) which is shown in the inset. We have now omitted the squares for clarity and instead highlight the data point corresponding to 10 mM Mg^{2+} with a black edge.

4. Delta_G in Fig. 5 is calculated by the Eyring model. How is this justified? Might a diffusion-based approach (e.g. Kramers) be better suited here?

Response: We have use transition state theory with a transmission coefficient κ=1 (i.e. Eyring model) to get a lower limit estimate of the energy barrier separating the unbound and bound state. A Kramer's theory approach might indeed give slightly more accurate values of the absolute barrier heights if the reconfiguration time of the unbound state can be measured. However, our global ϕ-analysis focuses on the relative change in $\Delta G^{\ddagger}$ when the exon is changed from RNA to DNA which is independent of the pre-exponential factor because the unbound hairpin is the same in both cases.

5. Why are two kinetic analysis presented, the cumulative dwell time and the HMM analysis. Is the latter (in combination with the BIC) used to select the model and to double check the results from the former, or is there more to this?

Response: Cumulative dwell-time plots is the *de facto* standard in the field to analyze single-molecule kinetic data. Recently, hidden Markov models are getting more attention to quantify interconversion rates between all resolvable states of a molecule.
In cumulative dwell time analysis, heterogeneity is characterized by a multi-exponential decay from a FRET state but the analysis gives no information about state connectivity. The hidden Markov model on the other hand partitions this so-called degenerate FRET state into several hidden microstates, which are interconnected by transition probabilities and emit a sequence of FRET observations. More recently, ensemble HMM approaches not only connect the transition probabilities of states observed within single trajectories, but over a whole set of trajectories. As such the trained HMM is a full representation of the kinetic system and the states can be interpreted in terms of conformational states of a biomolecule.
The reviewer is right that we use the Bayesian information criterion (BIC) to judge the ensemble HMM results in combination with prior biochemical knowledge of the system under study (i.e. one of the four microstates – the unbound hairpin coordinating Mg^{2+} – is probably inexistent). In this way, we reduce the model parameters and come up with a sensible model that is in agreement with single-molecule FRET data sets.
We then mapped the HMM back onto the dwell time decays by simulating a set of single-molecule traces based on the trained transition rates. From theses we computed cumulative

dwell time decays and compared them to the experimental distributions (Fig. S13) to validate the HMM, as noted correctly by the reviewer.

6. Why is the second off rate of the HMM calculated as the product of the interconversion rate and the first off rate? Please indicate these rates ($k_{off,2}$; k_{21}; $k_{off,1}$) in Fig. 3 or S10.

Response: Comparison of the dwell time decays and the HMM is not straightforward since it depends on the chosen model. We have tried to illustrate the concept in an additional subpanel of Fig. S14 and in Fig. 5a. Dissociation from the bound FRET state can start from either kinetic state 1 or 2. Decay from kinetic state 1 is fast and involves only one transition k_{01} which is visible by a change in FRET and is thus equal to $k_{off,1}$ in the dwell time distribution plot. Because the kinetic model has no direct link between kinetic state 2 and 0, exon unbinding from kinetic state 2 involves first a state transition which is undetectable by FRET, k_{21}, and only afterwards the transition, k_{10} visible by a change in FRET. Since the two events are sequential and not parallel, the two state transition rates are multiplied. We have summarized this in Fig. 5a where we also indicate the unbinding rates.

7. The frequent puckerings with correlation times in the mid picosecond range have only been seen in MD simulations. Is there any experimental evidence for that?

Response: We have quantified linewidths and peak integrals between H1' and H2' protons of dIBS1* in TOCSY experiments (Fig. S17). The crosspeaks show intermediate peak volumes which are significantly smaller than those expected for a C2'-endo conformation (as seen for residues U11 and U12). The peaks also appear broader which further suggests a dynamic equilibrium between sugar pucker conformations which is fast on the NMR timescale. More accurate estimates of the timescales of repuckering could be obtained by relaxation dispersion experiments (Clay, Nucleic Acids Res. 2017) which are, however, beyond the scope of this manuscript.

8. A logarithmic scale might be advantageous in Fig. S11.

Response: We thank the reviewer for this suggestion. We have tried both linear and logarithmic scales for this graph. Even on a logarithmic representation, the largely different ΔBIC values still require an individual scaling for each ionic condition and we have therefore decided to keep the linear scale.

9. Some links in the supplement are not resolved (e.g. "Error! Reference source not found" on page 3).

Response: We have corrected the missing links.

Reviewer #3 (Remarks to the Author):

Although the work presents some interesting data, I think that its overall significance does not justify publication in Nature Communications.

Understandably, the authors have tried to write the text in a way expected for high-profile journals. Unfortunately, this has often lead to overstatement and formulations that are difficult to understand. For example, in the introduction the text states: "While transient Mg2+ association has been identified as a primary cause of heterogeneity in RNA interactions, the feasibility of reversing the effect by consolidating degenerate states and reducing dissociation to a single off-rate has hardly been investigated. Here, the sugar pucker is of special interest as a structural feature that sets RNA and DNA apart and due to its major influence on backbone dynamics."

Response: We thank the reviewer for the critical assessment of our manuscript. In our revised manuscript we have rewritten large parts of the introduction to convey more precisely how kinetic heterogeneity in nucleic acids comes about and what effects it might have in the context of group II intron catalysis. We have restructured the Results section and made several alterations and additions to the Figures in the main text and the SI to illustrate certain concepts of heterogeneity more clearly.

It is not clear what the authors mean here. While I agree that transient Mg2+ association can affect RNA interactions' heterogeneity, numerous other factors are also certainly influential. Regarding the sugar pucker, it strongly affects shapes of canonical B-DNA and A-RNA helices, but generally the statement is not correct. The ability of the 2'-hydroxyl group of ribose to establish a rich spectrum of H-bonding interactions in RNA determines the difference between RNA and DNA in folded forms, and probably single-stranded ensembles. I also have doubts about the FRET technique's capability to resolve effects of transient Mg2+ binding, due to its inherently low resolution. Key references in this section are auto-citations to works 12 and 13. Moreover, although the FRET measurements appear to have been done correctly, it is very difficult to separate the direct experimental findings (i.e., the primary data) from the author's interpretations and even speculations. This is the main weakness of the work. Generally, the authors seem to draw conclusions from FRET data about phenomena that are simply too small-scale for the technique to resolve. They also over-extrapolate their findings obtained for a model system when discussing the splicing mechanism. In a quite authoritative fashion they assume very simple few-state behavior of the system, drawing insufficiently justified structural models from the low-resolution FRET data, in my opinion. There could be structured substates in the hairpin part as well as the single-strand, which may be sensitive to the presence of divalent ions. The hairpin seems long enough to be structured in the unbound state, and formation of the complex could involve partially bound states, etc. I am not stating that this happens, but the possibility cannot be excluded.

Response: We agree with the reviewer that as a spectroscopic distance ruler FRET can indeed often not resolve differences between Mg^{2+} deficient and Mg^{2+} bound tertiary contacts. This

is why Mg^{2+} bound and unbound states are degenerate with respect to a change in FRET. Because of the very broad dynamic range of fluorescence (ps to hours) these hidden states can still be resolved as long as their lifetimes are different and occur on the timescale of TIRF imaging (ms to seconds). In other words, Mg^{2+} unbinding is inferred from the dissociation of the exon by using an ensemble-based hidden Markov model. Thus, an apparent two-state system (in terms of FRET distance) is composed of three kinetics states (time dimension). It is entirely possible and likely that in the context of the full intron additional conformational states confound the exon-intron binding interaction. The kinetic framework presented herein, should serve as a basis to understand and evaluate the kinetics of the surely more complex intron RNA/RNP. We have included a short outlook on this topic in the discussion.

I also have doubts about the second part of the work, the explicit-solvent MD simulations. This research looks quite weak, and lacks clear connection with the rest of the study, despite multiple opposite verbal claims in the paper. The total simulation time of 800 ns is marginal in contemporary literature. Moreover, the paper only addresses the sugar pucker dynamics although there could also be other important system descriptors. Further, there are methodological concerns. The paper states that the simulations were done with the "AMBER99sb" force field, without providing any reference. However, AMBER99sb is a protein force field and cannot be used for nucleic acid simulations. There is another "AMBER" force field, AMBER99, which can be used for nucleic acids. However, nowadays it is widely considered as obsolete and should no longer be used; see, for example, http://ambermd.org/doc12/Amber18.pdf p. 33, for current recommendations regarding use of force fields in the original AMBER package for biomolecular simulations. The authors used some other program for their simulations, but as they employed AMBER force fields they should clearly have chosen the right version and provided unambiguous references for reproducibility. I would definitely not regard AMBER99 as a suitable choice for quantitative modelling. In addition, sugar puckering is an anomeric effect that classical force fields are unlikely to describe with quantitative accuracy. The authors provide no justification that their MD simulations are stable and correctly model the studied molecules, although there are many possible causes of inaccuracies.

Response: We agree with the reviewer that it is an outstanding challenge to bridge biomolecular simulations and experiments in terms of length and time scales. In our case, we face the problem that exon binding and unbinding is captured by FRET while the molecular details of the process remain hidden. MD simulation, on the other hand, provide an atomistic picture but never see an exon unbinding event.
As suggested by the reviewer we have repeated our simulations with the latest corrections to the Amber force field family (ff99 + bsc0 + χOL3, which corresponds to Amber-ff14) as recommended by the Amber guidelines. Additionally, we included another set of simulations where we placed Mg^{2+} at the binding sites suggested by NMR chemical shifts, NOE restraints to $[Co(NH_3)_6]^{3+}$ as well as Mn^{2+} induced line broadening. We have included a detailed description of all settings and references for reproducibility. Furthermore, we have added RMSD plots and time binned histograms of the phase angles of all residues in (d)IBS1* to Figs. S17/S19 in order to better assess convergence with respect to the RNA

dynamics and sugar puckering. The new set of simulations are now 2 μs for both systems and ionic conditions, which is significantly longer than the calculated (auto)correlation times of the sugar puckers. Importantly, the new calculations are fully in line with our previous findings and conclusions from the originally submitted manuscript.

I do not understand a key sentence in the presumably MD part of the Results: "This local rigidity is enforced by coordination of Mg2+, packing together first shell RNA ligands." Is that an MD result? Or a speculative suggestion? The MD methodology part does NOT mention any Mg2+ ions, and specifically states that K+ ions were used. The whole MD part appears superficial and unsophisticated, with (as mentioned) unclear connection to the rest of the paper. The authors draw far-reaching conclusions based on minimal computer-modelling, and I do not grasp how they could be derived from the presented MD data. Essentially, those data merely indicate that the bound RNA exon strand is dominantly in the C3'-endo pucker while the bound equivalent DNA strand is mostly in the C2'-endo pucker with some spread (Figure 4c). This is entirely consistent with common expectations, and I think does not require any justifying MD simulations. The helix is probably in an overall A-form conformation, dictated by the RNA hairpin strand which explains some "excursions" of the DNA strand into noncanonical pucker regions. DNA is known to readily convert from B-form to A-form helices, and it can sample B-A intermediates, which have been detected multiple times by X-ray crystallography. However, I do not understand how one could derive conclusions about thermodynamic stability from such observations.

Response: We have revised the MD section and describe how linewidths and peak integrals in NMR TOCSY experiments support multiple sugar pucker conformations in the DNA exon even though they are averaged out on the NMR timescale. The MD simulations show that the exchange between these pucker states happens on a picosecond to nanosecond timescale which is much shorter than the overall simulation length.

The title and Abstract also make exaggerated claims. For example, the Abstract states that "Molecular dynamics simulations reveal an unexpected structural link between heterogeneity and the sugar puckers at the exon-intron binding interface. While inter-nucleotide strain, induced by Mg2+ ions, locks the exon in place, sugar puckering alleviates this structural rigidity and promotes exon release." I do not understand how this could be inferred from the reported research. What is the nature of the link? What kind of inter-nucleotide strain? This appears to be simply a chain of impressive but vague statements.
My feeling is that the authors are trying to draw big conclusions, many of which cannot be directly derived from the presented research

Response: We thank the reviewer for this critical remark. We have adapted the title and the abstract accordingly.

Reviewer #4 (Remarks to the Author):

In this manuscript, the authors examine the kinetics of the exon/intron site binding in a self-splicing intron using single-molecule FRET and molecular dynamics. Dwell-times and transition rates for RNA/RNA as well as RNA/DNA hybrids (as would be the target in retrohoming) are examined as a function of K+ and Mg++ salts. Using Markov models trained on the FRET traces, it is revealed that unbinding rate is the major difference when it comes to the Mg++ dependent off-rates of the RNA/DNA hybrid. Furthermore, long molecular dynamics simulations are used to show that this could arise from fluctuations in sugar puckering between the RNA/RNA vs RNA/DNA hybrids, driven by strongly bound Mg++ ions at the binding interface.

Overall, this is a very strong and compelling work. The single-molecule analysis is particularly thorough and clearly demonstrates how varying the monovalent vs divalent salt concentration affects the thermodynamics and kinetics of the long-range interaction differently for RNA/DNA vs RNA/RNA complexes. This in of itself is a fascinating result.

The one major point that needs to be addressed is that it is unclear to what extent (if any) Mg++ is included in the molecular dynamics simulations. The authors go to great lengths to motivate that the observed sugar-puckering interconversion is putatively enforced by inner-sphere coordination of Mg++ at an interfacial location identified via CobaltHexamine binding in NMR. However, I can find no mention anywhere of how this was handled in the simulation (i.e. how many Mg++'s were simulated, where they were placed, what parameters were used, etc). If there are Mg++ present, a spatial location and dwell time analysis would seem appropriate to see if it matches the pucker interconversion frequency. If there are no Mg++ in the simulation then this must be throughly justified, as it would make it harder to support the authors conclusions that pucker interconversions are faciliated by bound Mg++. This point must be satisfactorily addressed for the story to be complete.

Response: We thank the reviewer for his appreciation of our single-molecule analysis. We agree with the reviewer that the parameters used in the MD simulations, including the type of ions, were not described with enough detail. We have now extended the corresponding Methods section with a description of all the settings.
In our previous MD simulations, we have deliberately refrained from including any Mg^{2+} ions in order to avoid sampling issues related to Mg^{2+} binding. These simulations have been carried out with the Amber-ff99 force field but without the latest backbone dihedral corrections and ion parameters. We have therefore repeated these simulations for both constructs and extended the simulation time to 2 µs each. The simulations are fully consistent with the original ones and with overall better statistics due to the longer simulation time.
We understand the reviewer's argument that Mg^{2+} ions would be needed to bridge the simulations and the experiments. Due to the significantly longer residence times on nucleic acids compared to K^+, an accurate description of all possible binding sites would require either unreasonably long simulations (usually > ms depending on the coordination type) or enhanced sampling techniques. Implementations of such methods for predicting and understanding Mg^{2+} binding to RNA tertiary contacts are actively developed in our lab.

Because there are already a number of binding positions proposed in EBS1*/(d)IBS1* by NMR chemical shifts and NOE restraints, we have now used these positions as starting points for a second set of MD simulations. We have visualized the spatial distribution of Mg^{2+} over the course of the simulation (Fig. S16). As expected from experimentally observed Mg^{2+} residence times on RNA, the metal ions do not unbind within the 2 μs of the simulation but fluctuate within the binding pocket. For a kinetic analysis of Mg^{2+} dwell times, as suggested by the reviewer, sampling of multiple binding and unbinding events would be required but is unfortunately impractical with currently available computation power even on supercomputers like Piz Daint (CSCS, Swiss National supercomputing center) where we run our simulations.

There are also some minor typographical issues. In Figure 3A, state 1 is assigned 63% population and state 0 37%, yet the state 0 is given the larger circle area. In the SI, pages 2 and 3 there is a bold "Error! Reference source not found"

Response: We thank the reviewer for spotting these typographical mistakes which we have corrected in the revised version of our manuscript.

REVIEWERS' COMMENTS:

Reviewer #1 (Remarks to the Author):

Steffen et al. have thoroughly addressed my comments on the original manuscript. They better explain how they distinguish between heterogeneity between molecules and between conformational states (that may be sampled by any single RNA). They also introduce how the sugar pucker states are inferred from NMR TOCSY experiments. The most important improvement is that they redid the MD simulations using up to date force field parameters for RNA and for longer times, so that the results are more convincing. The combined experimental data and simulations support the conclusion that site-binding of Mg2+ ions to the RNA-RNA complex results in longer lived RNA complexes relative to RNA-DNA complexes, and that this correlates convincingly with changes in sugar pucker conformation and dynamics.

Reviewer #2 (Remarks to the Author):

The authors have addressed all my concerns. In particular:
- I appreciate the change in title, which now focusses on the main result, namely that ion binding and sugar puckering are two interacting processes that shape kinetic heterogeneity.
- The model and the key messages are much clearer now and better supported by literature.
- The additional simulations, analysis of NMR spectra and Supplemental Figures further support the results.

Therefore, I do now fully support publication in Nature Communications.

The authors might want to consider the following two minor points:
- In my opinion the new discussion on pseudo-knots is not necessary.
- Line 296: "... a spectroscopic ruler, FRET fails to resolve these kinetic states" - Here I believe that not "FRET" fails to resolve these states, but the "FRET-efficiency".

Reviewer #3 (Remarks to the Author):

The paper has been considerably revised. I think the authors have made all specific revisions that were doable. The tone of the paper has been moderated, the experiments are better explained and the MD simulation part appears significantly improved. Still, the link between the FRET experiments and MD remains not fully convincing. I agree with the Reviewer #1, the simulations can indicate plausible explanation but are not sufficient to prove it. The kinetic model in Fig. 3 could be still an oversimplification due to overlapping processes; HMM is not a panacea. However, these uncertainties also reflect the genuine limits of the used experimental and theoretical techniques within the context of complexity of the studied process. In other words, I do not think the authors could do anything better with the presently available methods. The primary experimental results are definitely interesting and stimulating. Thus, I have no specific criticism or suggestion for further revisions, except of replacing the word “reveal” by “suggest” in the abstract and adding the missing Journal title to ref. 37 (should be Biochemistry).

Reviewer #4 (Remarks to the Author):

In their response to reviewer comments, the authors have provided a much more thorough explanation of the detailed simulation protocols, including new simulations including Mg++, a spatial

distribution analysis of the ions, and longer simulations.

Most significantly, the revised manuscript includes additional NMR data directly reporting on the difference in sugar pucker behavior between the RNA/RNA and DNA/RNA systems. This enabled the authors to make their point almost entirely from the experimental data alone which is a significant improvement because they no longer have to rely on simulation predictions for establishing sugar pucker interconversion which are considered weak in modern force-fields. In the current revision, the simulations merely serve to guide interpretation of the experimental data while the experimental evidence now stand on their own. In my opinion this is now a much stronger manuscript and I see no reason why it should not be published.

University of Zurich[UZH]

Department of Chemistry

University of Zurich
Winterthurerstrasse 190
CH-8057 Zurich
Phone +41 44 635 46 54/80
Fax +41 44 635 68 02
http://www.chem.uzh.ch/rna

Reviewer Comments

Reviewer #1

Steffen et al. have thoroughly addressed my comments on the original manuscript. They better explain how they distinguish between heterogeneity between molecules and between conformational states (that may be sampled by any single RNA). They also introduce how the sugar pucker states are inferred from NMR TOCSY experiments. The most important improvement is that they redid the MD simulations using up to date force field parameters for RNA and for longer times, so that the results are more convincing. The combined experimental data and simulations support the conclusion that site-binding of Mg2+ ions to the RNA-RNA complex results in longer lived RNA complexes relative to RNA-DNA complexes, and that this correlates convincingly with changes in sugar pucker conformation and dynamics.

Response: We thank the reviewer for the positive feedback on our revised manuscript.

Reviewer #2

The authors have addressed all my concerns. In particular:
- I appreciate the change in title, which now focusses on the main result, namely that ion binding and sugar puckering are two interacting processes that shape kinetic heterogeneity.
- The model and the key messages are much clearer now and better supported by literature.
- The additional simulations, analysis of NMR spectra and Supplemental Figures further support the results.

Therefore, I do now fully support publication in Nature Communications.

The authors might want to consider the following two minor points:
- In my opinion the new discussion on pseudo-knots is not necessary.
- Line 296: "... a spectroscopic ruler, FRET fails to resolve these kinetic states" - Here I believe that not "FRET" fails to resolve these states, but the "FRET-efficiency".

Response: We thank the reviewer for the support in publishing our revised manuscript. We have introduced the brief discussion on pseudoknots in the light of a reviewer comment suggesting to us to elaborate on the origin of the hairpin-exon construct. In group II introns the EBS1/(d)IBS1 recognition element acts in a very similar way to other small ribozymes or riboswitches in which a pseudoknot interaction forms the catalytic core or the ligand binding aptamer domain. We kept but slightly adapted the short introduction to make this clearer. We have changed the phrasing on line 296 as suggested by the reviewer.

Reviewer #3

The paper has been considerably revised. I think the authors have made all specific revisions that were doable. The tone of the paper has been moderated, the experiments are better explained and the MD simulation part appears significantly improved. Still, the link between the FRET experiments and MD remains not fully convincing. I agree with the Reviewer #1, the simulations can indicate plausible explanation but are not sufficient to prove it. The kinetic model in Fig. 3 could be still an oversimplification due to overlapping processes; HMM is not a panacea. However, these uncertainties also reflect the genuine limits of the used experimental and theoretical techniques within the context of complexity of the studied process. In other words, I do not think the authors could do anything better with the presently available methods. The primary experimental results are definitely interesting and stimulating. Thus, I have no specific criticism or suggestion for further revisions, except of replacing the word “reveal” by “suggest” in the abstract and adding the missing Journal title to ref. 37 (should be Biochemistry).

Response: We thank the reviewer for the positive feedback on our revised manuscript. We have replaced the wording in the abstract and included the missing journal title as indicated by the reviewer.

Reviewer #4

In their response to reviewer comments, the authors have provided a much more thorough explanation of the detailed simulation protocols, including new simulations including Mg++, a spatial distribution analysis of the ions, and longer simulations.

Most significantly, the revised manuscript includes additional NMR data directly reporting on the difference in sugar pucker behavior between the RNA/RNA and DNA/RNA systems. This enabled the authors to make their point almost entirely from the experimental data alone which is a significant improvement because they no longer have to rely on simulation predictions for establishing sugar pucker interconversion which are considered weak in modern force-fields. In the current revision, the simulations merely serve to guide interpretation of the experimental data while the experimental evidence now stand on their own. In my opinion this is now a much stronger manuscript and I see no reason why it should not be published.

Response: We thank the reviewer for the positive assessment of our revised manuscript.
